# Supramolecular strategy for compartment pathogen clearance and immuno-metabolic homeostasis to treat periodontitis

**DOI:** 10.1038/s41467-026-74544-5

**Published:** 2026-06-18

**Authors:** Bingfeng Wu, Liu Liu, Junheng Zhu, Dongzhe Song, Zhenming Wang, Dingming Huang, Ling Ye

**Affiliations:** 1 State Key Laboratory of Oral Diseases & National Center for Stomatology & National Clinical Research Center for Oral Diseases, West China Hospital of Stomatology, Sichuan University, Chengdu, Sichuan China; 2 Department of Conservative Dentistry and Endodontics, West China Hospital of Stomatology, Sichuan University, Chengdu, Sichuan China

**Keywords:** Periodontitis, Immune evasion, Infection

## Abstract

Periodontitis is an inflammatory disease driven by bacterial infection and immune dysfunction. Immune-subversive bacteria in the periodontitis microenvironment, such as *Porphyromonas gingivalis*, can evade conventional therapies by invading cells and inducing lysosomal dysfunction. Here we develop an injectable supramolecular hydrogel through the co-assembly of recombinant human type I collagen (COL), poly-ε-lysine (PL), and puerarin (PUE). Supramolecular amorphization improves PUE’s solubility and permeability, enabling a dual-compartment antibacterial strategy via effective trans-barrier delivery. Extracellularly, PL and PUE synergistically disrupt bacterial membranes and metabolism, while concurrently mitigating bacterial toxin induced pro-inflammatory macrophage polarization. Intracellularly, the supramolecular complexes facilitate PUE accumulation in phagolysosomes. By counteracting local oxidative stress, the internalized PUE restores lysosomal acidification and alleviates bacteria-induced immuno-metabolic dysregulation. In vivo, the hydrogel manipulates the local inflammatory microenvironment and facilitates periodontal tissue repair. This study provides a clinically translatable supramolecular strategy for treating intracellular infections and restoring tissue homeostasis.

## Abstract (web summary)

Intracellular bacteria can evade immune response by triggering lysosomal dysfunction. Here, the authors report a supramolecular strategy using the coassembly of collagen I, poly-ε-lysine, and puerarin, for extra- and intracellular pathogen clearance, restoring lysosomal function and immuno-metabolic homeostasis.

## Introduction

Periodontitis is a highly prevalent chronic bacterial infection and the leading cause of tooth loss^1^. It is strongly associated with severe systemic diseases, including Alzheimer’s disease and cardiovascular disease, and periodontal therapy has been shown to improve outcomes in these conditions^2,3^. Periodontitis is now understood not merely as a bacterial infection, but as a complex inflammatory disease driven by host immune dysfunction. It is initiated by oral microbial dysbiosis, and further manifests as persistent immune cell-mediated inflammatory responses, which ultimately results in progressive destruction of the tooth-supporting tissues^4^.

Biomaterials for periodontitis treatment have shown promise in modulating the immune microenvironment and eliminating both planktonic bacteria and bacterial biofilms. Nevertheless, the chronic and destructive inflammation of periodontitis remains difficult to reverse^5^. Recently, it has been revealed that immune-subversive bacteria in the periodontitis microenvironment, such as *Porphyromonas gingivalis* (*P. gingivalis*), can selectively evade immune clearance without suppressing inflammation^6,7^. As an immune evasion strategy, *P. gingivalis* persists intracellularly in phagocytes by blocking phagocytosis pathways, inducing lysosomal dysfunction and promoting pro-inflammatory responses^8–10^. Since the maintenance of lysosomal acidification via proton transport mechanisms is a central approach for lysosomal homeostasis, its disruption by bacteria triggers an immuno-metabolic dysfunction that impairs the bactericidal capacity of phagocytes, allowing further intracellular bacterial persistence^11^. Meanwhile, excessive release of inflammatory mediators exacerbates chronic inflammation and tissue damage, establishing a vicious cycle that further compromises pathogen clearance and promotes local tissue destruction^12^. Therefore, effective biomaterials for treating periodontitis should not only eliminate extracellular bacteria but also eradicate persistent bacteria within phagocytes, particularly macrophages, which serve as the first line of defense against invading pathogens^12,13^.

To address this challenge, conventional therapies rely on long-term, high-dose antibiotic administration to achieve sufficient intracellular concentrations^14^. However, treatment failure and recurrence are common, largely due to poor membrane permeability, limited intracellular accumulation, and reduced antibiotic efficacy in the harsh phagolysosomal environment^15^. Nanomaterial-based delivery systems have shown potential for integrating multiple strategies to eliminate intracellular bacteria, but their clinical translation is hindered by biotoxicity concerns, complex and costly fabrication processes, and difficulties in scalable production^16,17^. Consequently, there is an urgent need for a safe, easily fabricated, and low-cost delivery system capable of immuno-metabolic remodeling following macrophage uptake, thereby reversing immune-subversive macrophage states and enabling clearance of persistent intracellular bacteria.

Here, we propose a materials design strategy based on supramolecular functional integration, in which multiple synergistic components were assembled through supramolecular interactions into an integrated system. As a proof of concept, poly-ε-lysine (PL), puerarin (PUE) and recombinant human type I collagen (COL) were selected to construct a ternary supramolecular system (PL/PUE/COL). To address pathogen clearance, PL, an FDA-approved cationic antimicrobial polypeptide, was incorporated as the primary antibacterial component. Its cationic nature allows PL to adhere to the negatively charged bacterial surfaces, stripping away the outer membrane and leading to direct bacterial death^18–20^. In addition, the cationic chains of PL promote strong electrostatic adhesion to negatively charged mucosal surfaces and enhance epithelial permeability, ensuring efficient delivery into deep periodontal tissues^21,22^. However, extracellular bacterial elimination alone is insufficient to eradicate pathogens such as *P. gingivalis*, which evade immune clearance and lysosomal digestion by invading host cells.

To enhance intracellular bacterial clearance and resolve chronic inflammation, PUE, a unique C-glycoside isoflavone (8-C-glucopyranosyl daidzein), was incorporated as an immuno-metabolic regulatory component^23–32^. Unlike common flavonoids whose O-glycoside structures are prone to rapid hydrolytic degradation in acidic and enzymatic microenvironment, the stable C-C glycosidic bond of PUE confers exceptional resistance against acid and enzymatic degradation^33,34^. This stability is critical for maintaining bioactivity within the acidic phagolysosomal microenvironment. Through its potent reactive oxygen species (ROS) scavenging capacity and immuno-metabolic regulatory effects, PUE may help preserve lysosomal homeostasis under bacterial challenge^23–32,35^. Nevertheless, intracellular application of PUE is limited by poor solubility and restricted tissue permeability arising from its C-glycoside isoflavone structure^36,37^. Unlike ideal amphiphilic drugs that possess a balanced lipophilicity to facilitate passive diffusion, PUE exhibits a polar-biased amphiphilicity^38^. This creates a dual barrier: the hydrophilic moiety restricts passive transmembrane diffusion by lowering lipid partitioning, while the hydrophobic backbone promotes crystalline aggregation, preventing stable molecular dispersion in aqueous media.

To overcome these limitations, COL and PL were employed to collaboratively modulate the physicochemical state of PUE through supramolecular co-assembly. As the primary matrix component of periodontal tissues, COL plays a pivotal role in tissue homeostasis and repair^4,39–41^. More importantly in this system, it provides abundant hydrophobic regions that serve as molecular pockets to accommodate the hydrophobic isoflavone backbone of PUE, thereby preventing its crystalline aggregation^42,43^. Meanwhile, the cationic polypeptide PL is integrated into the matrix via non-covalent interactions. Such co-assembly of PL/PUE/COL achieves supramolecular functional integration, enhancing PUE amorphization, transmembrane diffusion, and phagolysosomal accumulation through cellular endocytosis of the supramolecular complexes^44^.

**Fig. 1:**
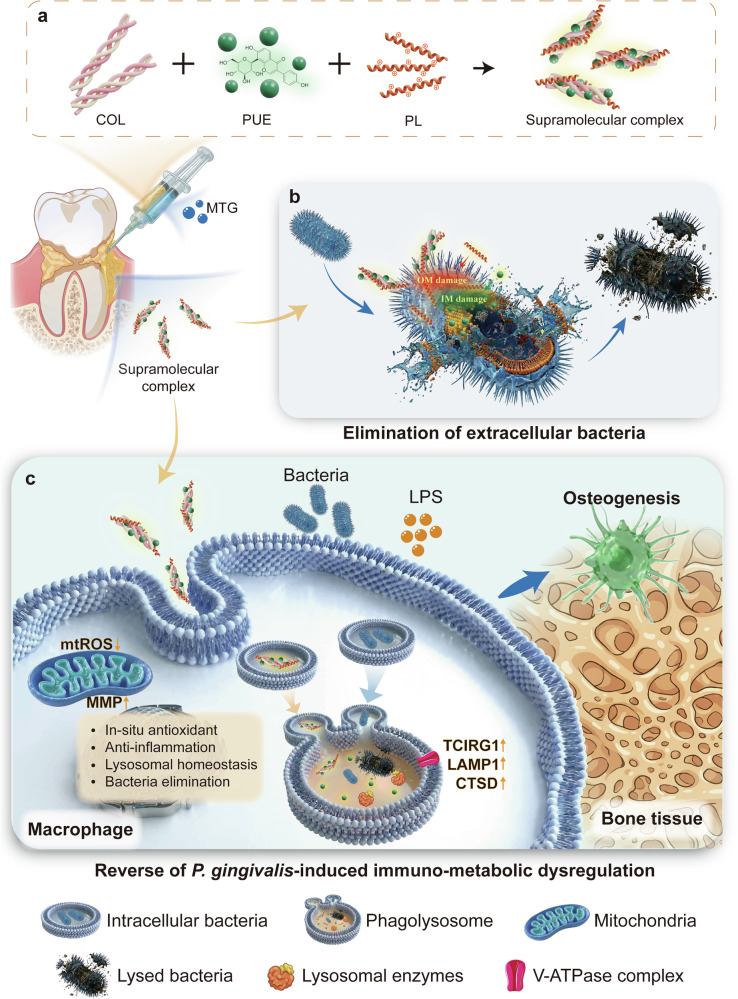
Schematic diagram of PL/PUE/COL supramolecular hydrogel. **a** Preparation of PL/PUE/COL supramolecular system. Mechanisms of **b** extracellular bacteria elimination and **c** reversion of bacteria-induced immuno-metabolic dysregulation. PL poly-ε-lysine, PUE puerarin, COL recombinant human type I collagen, MTG microbial transglutaminase, OM outer membrane, IM inner membrane, LPS lipopolysaccharide, mtROS mitochondrial reactive oxygen species, MMP mitochondrial membrane potential, TCIRG1 T-cell immune regulator 1, LAMP1 lysosomal-associated membrane protein 1, CTSD cathepsin D.

To ensure sustained retention and stable therapeutic delivery within the narrow, fluid-flushed periodontal environment, the supramolecular complex was further integrated into a macroscopic crosslinked hydrogel network. The crosslinked PL/PUE/COL supramolecular hydrogel can release soluble supramolecular fragments within the periodontitis microenvironment, enabling a dual-compartment antibacterial strategy. Extracellularly, these fragments exerted synergistic antibacterial effects, while intracellularly, they acted as “Trojan horses” to eliminate intracellular bacteria. These PUE-containing supramolecular fragments were efficiently endocytosed by macrophages and transported to lysosomes, which effectively bypassed the poor transmembrane diffusion ability of PUE. The supramolecular functional integration reversed the *P. gingivalis*-induced immuno-metabolic dysregulation and disrupted the “chronic inflammation-immune evasion” vicious cycle in periodontitis, providing a safe, easily fabricated, low-cost, and highly effective biomaterials approach for treating periodontitis and other intracellular bacterial infections (Fig. 1).

## Results

### Supramolecular co-assembly and enhanced trans-barrier delivery

**Fig. 2:**
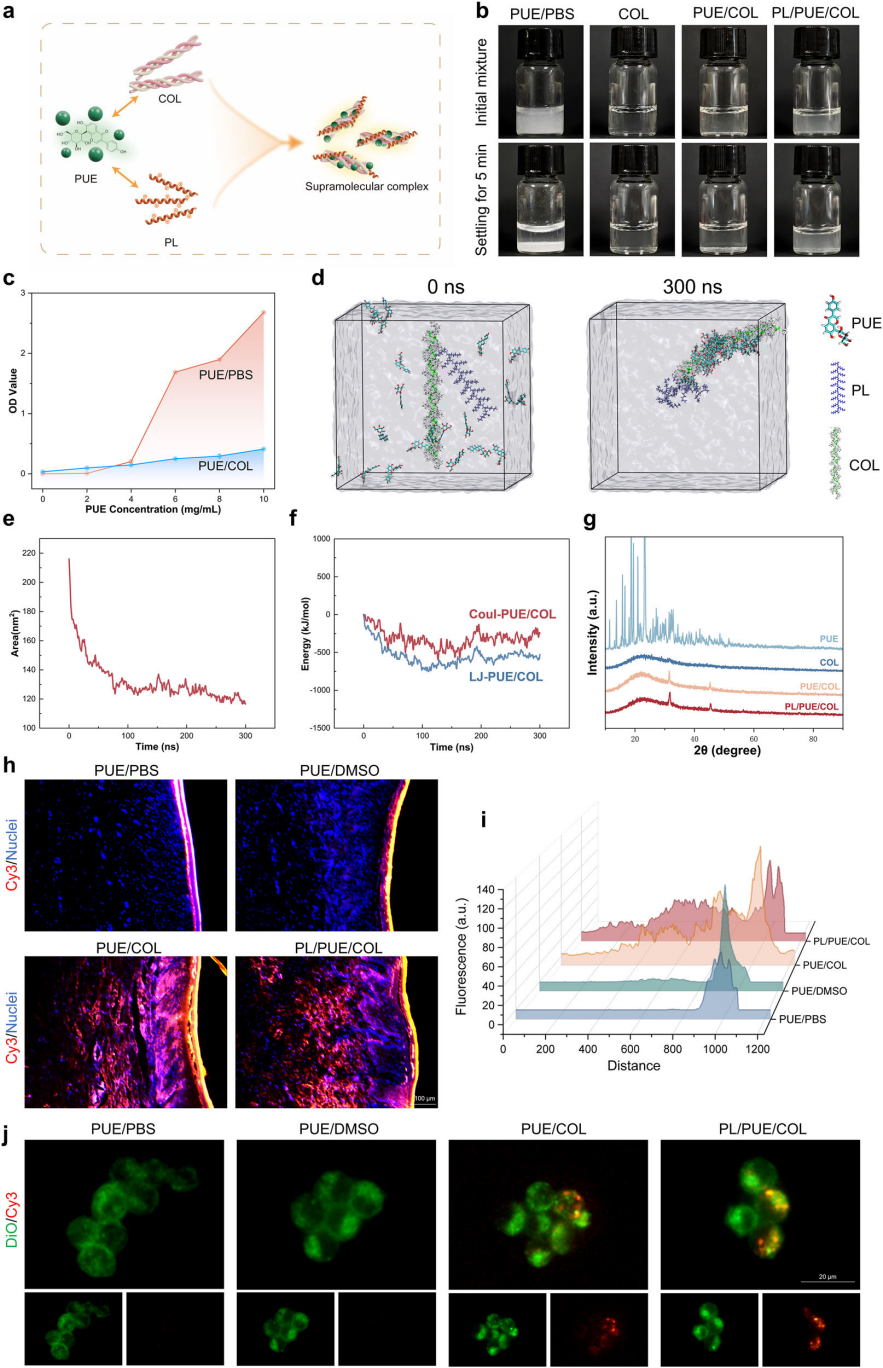
Synthesis and characterization of supramolecular co-assembly system. **a** Schematic illustration of the synthetic procedures for the PL/PUE/COL supramolecular system. **b** Optical images comparing the solubility and sedimentation of PUE in PBS, COL solution, and PL/COL solution. **c** The OD values of PUE with gradient concentrations (0, 2, 4, 6, 8, 10 mg/mL) dispersed in PBS and COL solution, respectively (*n* = 3). **d** Representative snapshot taken from MD simulation displaying the interaction of PUE, PL and COL in water during 300 ns simulation. **e** SASA curves in the PL/PUE/COL system over time. **f** Interaction energy between PUE and COL over time. **g** XRD results for PUE, COL, PUE/COL, and PL/PUE/COL. **h** Trans-epithelial delivery of Cy3-labeled PUE (red) from PUE/PBS, PUE/DMSO, PUE/COL, and PL/PUE/COL in porcine gingival mucosa. The images were representative of three independently repeated experiments from each group. **i** Spatial distribution profiles of Cy3 fluorescence signals in Fig. 2h. **j** Cellular internalization of Cy3-labeled PUE (red) from PUE/PBS, PUE/DMSO, PUE/COL, and PL/PUE/COL in macrophages. The images were representative of three independently repeated experiments from each group. PL poly-ε-lysine, PUE puerarin, COL recombinant human type I collagen, OD optical density, PBS phosphate-buffered saline, MD molecular dynamics, SASA solvent-accessible surface area, Coul Coulomb interactions, LJ Lennard-Jones potentials, XRD X-ray diffraction, DMSO dimethyl sulfoxide. Source data are provided as a Source Data file.

To construct the PL/PUE/COL supramolecular system, we employed a co-assembly strategy via a facile one-pot method (Fig. 2a). Macroscopically, the PUE/COL and PL/PUE/COL solution were transparent and homogeneous, similar to the pure COL solution. In contrast, the PUE/PBS solution showed a turbid suspension with rapid sedimentation, attributed to self-aggregation driven by the strong hydrophobic interaction as previously reported^36^ (Fig. 2b). We used varying concentrations of PUE to further validate the solubilization capacity of the supramolecular system. As the PUE concentration elevated from 0 to 10 mg/mL, the turbidity of PUE/PBS solution increased in a concentration-dependent manner, followed by significant sedimentation after 5 min (Supplementary Fig. 1a). In contrast, the PUE/COL supramolecular system remained clear without turbidity or sedimentation at a high concentration of 10 mg/mL (Supplementary Fig. 1b). The transparency was maintained at 37 °C for over 5 months, demonstrating exceptional macroscopic stability (Supplementary Fig. 1c). Quantitatively, the PUE/PBS group exhibited a sharp increase in optical density (OD) values with increasing PUE concentration, indicative of intense light scattering caused by the formation of large, insoluble aggregates (Fig. 2c). However, the PL/PUE/COL system maintained low scattering signals close to the pure COL, confirming that the supramolecular co-assembly effectively suppressed PUE aggregation and realized the effective dispersion of PUE within supramolecular matrix. Considering the application of the supramolecular complex in the oral environment, we further investigated its assembly stability in artificial saliva (AS). While the direct addition of PUE into AS led to macroscopic turbidity and precipitation, the PUE/COL/AS supramolecular system maintained a stable condition without visible sedimentation, exhibiting a slight opalescence (Supplementary Fig. 2). It confirms that the supramolecular co-assembly can be effectively achieved and maintained in the complex environment of saliva.

To elucidate the underlying molecular mechanisms driving this macroscopic solubilization, we performed molecular dynamics (MD) simulations. As depicted in the simulation snapshots, the initially dispersed PUE, PL, and COL molecules rapidly aggregated and spontaneously reorganized into a highly compact ternary cluster (Fig. 2d). As evidenced by the root mean square deviation (RMSD) profiles, the entire supramolecular cluster rapidly converged to a stable equilibrium following initial structural fluctuations, indicating the formation of a dynamically stable aggregate (Supplementary Fig. 3a). Importantly, the RMSD of the COL backbone remained consistently low throughout the assembly process, indicating that the structural integrity of the COL was not compromised (Supplementary Fig. 3b). The structural compaction was quantitatively corroborated by a significant reduction of over 40% in the solvent-accessible surface area (SASA), revealing the burial of hydrophobic isoflavone backbone of PUE to form a dense co-assembly (Fig. 2e). As shown in Supplementary Fig. 3c, the contacts between PUE and COL increased sharply within the first 50 ns and subsequently reached a stable plateau, which indicates a high binding affinity. Following this rapid adsorption, the system underwent continuous spatial adjustments to minimize the total energy, corresponding to the gradual decrease in SASA (Fig. 2e).

Furthermore, quantitative evaluation of Coulomb interactions and Lennard-Jones potentials demonstrated that the interaction energy between PUE and the COL scaffold was predominantly governed by Lennard-Jones potentials (Fig. 2f). Structurally, the proline-rich triple-helical conformation of COL provides abundant hydrophobic regions that effectively accommodate the hydrophobic isoflavone core of PUE^43,45^. The hydrophobic packing was further stabilized by a dense intermolecular hydrogen-bonding network between the abundant amide/hydroxyl groups of COL and the phenolic hydroxyls of PUE (Supplementary Fig. 3d). Specific hydrogen bond anchoring sites were clearly identified in the simulated structural details (Supplementary Fig. 4). Interestingly, the interaction energy between PL and PUE was nearly negligible, while the interaction between the PL and COL was driven by Coulomb interactions (Supplementary Fig. 5). This strong attraction is attributed to the high-affinity anchoring of PL’s abundant amino groups to COL. Significantly, the assembly mode corroborates the successful supramolecular functional integration, which allows the system to effectively stabilize the amorphous PUE molecules without PUE interfering with or masking the intrinsic cationic antibacterial sites of PL.

The assembly mechanisms predicted by MD simulations were further validated through experimental characterizations. X-ray diffraction (XRD) analysis showed that pure PUE exhibited sharp diffraction peaks characteristic of a crystalline lattice. However, these crystalline peaks were largely suppressed in both PUE/COL and PL/PUE/COL systems. The amorphization indicates that the intermolecular interactions within the supramolecular matrix disrupted the long-range ordered arrangement of PUE, facilitating its dispersion (Fig. 2g). To confirm the specific non-covalent interactions within the supramolecular system, ^1^H nuclear magnetic resonance (NMR) spectroscopy was performed (Supplementary Fig. 6a). In the locally enlarged spectra (δ 9.0–10.5 ppm), the characteristic sharp peak of PUE’s phenolic hydroxyl protons at approximately δ 9.6 ppm was suppressed in the PUE/COL co-assembly system (Supplementary Fig. 6b). This signal attenuation indicates the interactions between the phenolic hydroxyl groups of PUE and the COL matrix. The molecular-level integration was also supported by Fourier transform infrared (FTIR) and differential scanning calorimetry (DSC) analyses. The FTIR spectra of the PUE/COL and PL/PUE/COL supramolecular complexes exhibited the suppression of PUE’s characteristic peaks (1000–1650 cm^−^¹) (Supplementary Fig. 7a). The characteristic peaks were associated with its aromatic rings and glycosidic structures, suggesting that PUE was effectively encapsulated and its molecular vibrations were restricted by the supramolecular environment. DSC analysis further evaluated the thermodynamic state of the supramolecular system. As shown in Supplementary Fig. 7b, COL and PUE exhibited endothermic peaks at 58.64 and 58.56 °C, respectively. Upon co-assembly, the characteristic peak of PUE/COL supramolecular complex shifted significantly to a higher temperature at 66.22 °C, representing an elevation of ~7.6 °C. Mechanistically, the intermolecular hydrogen bonding and hydrophobic interactions restrict the segmental mobility of the COL and PL polypeptide chains. As a result, higher thermal energy is required to disrupt these reinforced molecular networks, confirming the formation of a highly compatible supramolecular system. While FTIR and DSC analyses also detected weak interactions in the binary PL/PUE mixture, these are negligible in the ternary system, where the significantly stronger PUE-COL and PL-COL interactions dominate the assembly process (Supplementary Fig. 8).

Crucially, the supramolecular co-assembly did not compromise the intrinsic bioactivity of PUE. The antioxidant capacity was assessed via the 2,2-diphenyl-1-picrylhydrazyl (DPPH) radical scavenging assay (Supplementary Fig. 9). The radical clearance rate of the PUE/COL groups increased in a concentration-dependent trend, even approaching nearly 100% at the PUE concentrations of 8 and 10 mg/mL. Furthermore, the PL/PUE_10_/COL supramolecular complex also exhibited an excellent scavenging efficacy comparable to that of PUE_10_/COL, significantly beyond the PL/COL group and COL group. It demonstrates that PUE retains its antioxidant capability when integrated into the supramolecular complex. More importantly, by overcoming the inherent aqueous insolubility which practically impedes the use of free PUE in physiological fluids, the supramolecular strategy provides an effective formulation for physiological administration, laying the foundation for modulating the local oxidative stress microenvironment.

Given that efficient mucosal permeation is a prerequisite for local periodontitis therapy, we investigated the trans-barrier delivery capacity of the supramolecular complex. The penetration behavior within periodontal tissues was assessed using an ex vivo porcine gingiva model. After PUE was labeled with Cy3, PUE suspension in PBS, PUE solution in dimethyl sulfoxide (DMSO), PUE/COL, and PL/PUE/COL were applied to the surface of the gingival tissues, respectively (Fig. 2h). In the PUE/PBS group, fluorescence signals were predominantly confined to the superficial surface of the gingiva, indicating negligible penetration into the epithelium. It was consistent with the rapid crystallization of PUE in aqueous environments, which created large aggregates that are physically blocked by the dense mucosal barrier. Interestingly, the PUE/DMSO group, where PUE is fully dissolved in organic solvent, exhibited limited infiltration within the epithelial layer. This suggested that although solubility was a prerequisite, the polar-biased amphiphilicity and lack of tissue retention still restricted the penetration ability of PUE. In contrast, the PUE/COL and PL/PUE/COL supramolecular complexes demonstrated deep tissue penetration, where PUE molecules not only permeated the epithelium but also were distributed in the lamina propria. Quantitative analysis of the fluorescence spatial distribution further corroborated the significantly enhanced tissue penetration capability of PUE-based supramolecular complexes (Fig. 2i). Furthermore, we investigated the supramolecular-mediated intracellular uptake of PUE using fluorescent dye Cy3-labeled PUE. Macrophages were incubated with PUE/PBS, PUE/DMSO, PUE/COL, and PL/PUE/COL, respectively. As shown in Fig. 2j, the PUE/PBS and PUE/DMSO groups exhibited negligible intracellular red fluorescence signals. In comparison, the PUE/COL and PL/PUE/COL groups displayed pronounced fluorescence, indicating substantial cellular internalization of PUE. It confirmed that the specific molecular structure of PUE hinders the passive diffusion across the cell membrane, even when fully dissolved in DMSO.

We attributed this superior performance to a synergistic mechanism involving the amorphization of PUE via supramolecular co-assembly and the permeation-enhancing effects of the cationic PL. Specifically, the PL facilitated strong electrostatic interactions with the anionic mucosa and enhanced epithelial permeability, which subsequently propelled gradient-mediated transport into the underlying tissues^22^. The uptake enhancement via supramolecular complexes indicated a shift in the cellular entry pathway. Unlike free PUE molecules which struggled to cross the lipid bilayer, the supramolecular complexes were recognized and actively internalized by macrophages via endocytosis. Consequently, the PL/PUE/COL system successfully overcomes both the mucosal tissue barrier and the cellular membrane barrier, achieving efficient intracellular accumulation.

### Fabrication and characterization of PL/PUE/COL supramolecular hydrogel

**Fig. 3:**
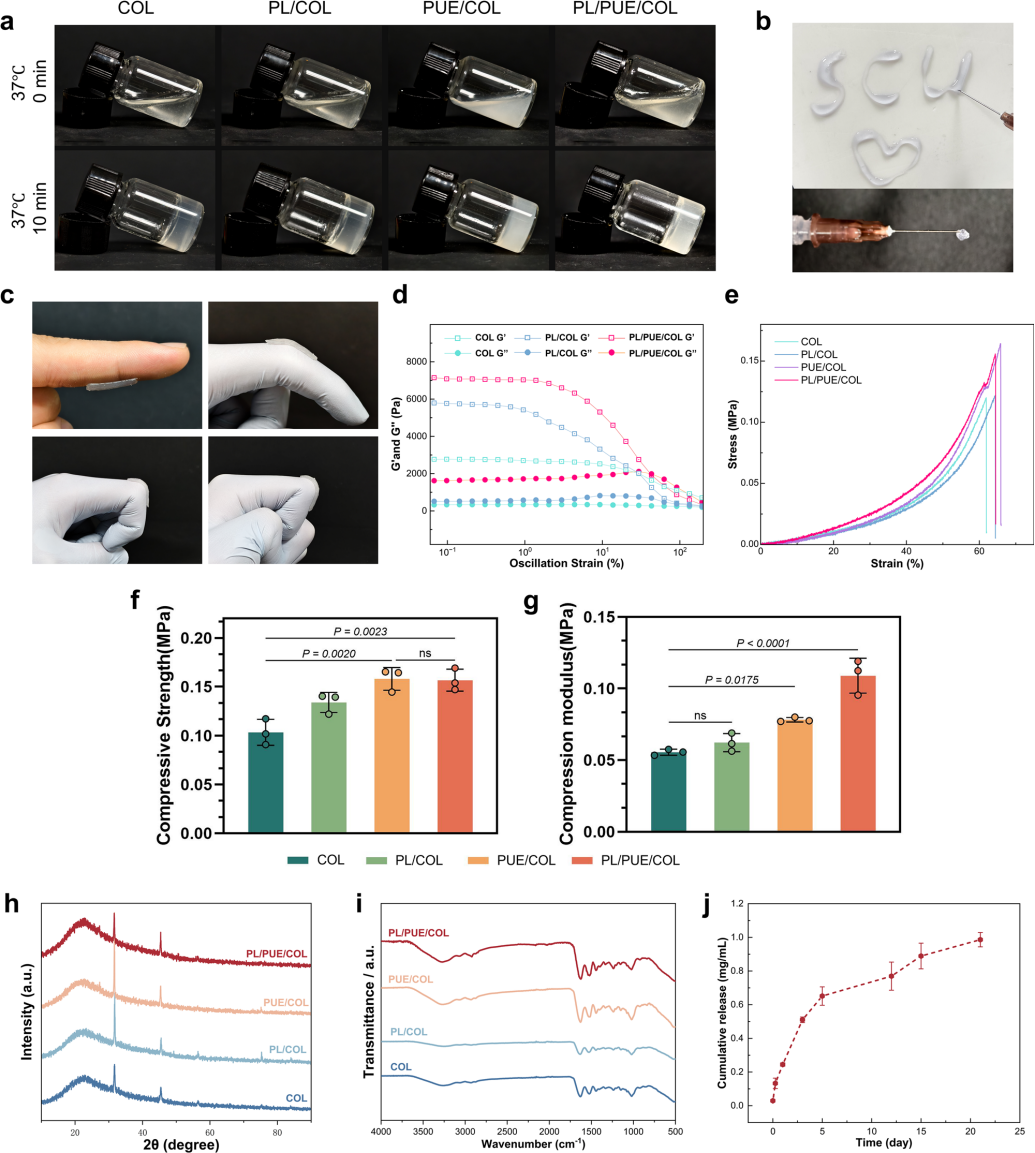
Fabrication and characterization of PL/PUE/COL supramolecular hydrogel. **a** Optical characterization images of the COL, PL/COL, PUE/COL, and PL/PUE/COL hydrogels after cross-linking. **b** Injectability and **c** adhesive behavior of PL/PUE/COL supramolecular hydrogel. **d** Storage modulus (G’) and loss modulus (G’’) of COL, PL/COL and PL/PUE/COL hydrogels at a constant normal force of 1 N and a frequency of 10 rad/s. **e** Stress-strain curves, **f** compressive strength (*n* = 3 independent experiments) and **g** compressive modulus of COL, PL/COL, PUE/COL, and PL/PUE/COL hydrogels by mechanical tests (*n* = 3 independent experiments). **h** XRD patterns and **i** FTIR spectra of freeze-dried COL, PL/COL, PUE/COL, and PL/PUE/COL hydrogels. **j** Accumulative release curve of PUE within 21 days (*n* = 3 independent experiments). All error bars represent mean ± *SD*. *P* values were calculated by one-way analysis of variance (ANOVA) followed by Tukey’s post hoc test (ns not significant). PL poly-ε-lysine, PUE puerarin, COL recombinant human type I collagen, XRD X-ray diffraction, FTIR Fourier transform infrared spectrometry. Source data are provided as a Source Data file.

PL/PUE/COL supramolecular hydrogel was obtained by the addition of microbial transglutaminase (MTG) crosslinker into the PL/PUE/COL system. Scanning electron microscopy (SEM) observation revealed that the freeze-dried hydrogel possessed a porous interconnected structure (Supplementary Fig. 10). The gelation process was recorded photographically (Fig. 3a). To confirm the injectability, PL/PUE/COL hydrogel was extruded through a 26G needle to write “SCU” in Fig. 3b. The excellent injectability and sufficient operation time ensured the effective clinical management of periodontal defects with complexity of deep, narrow pockets. To reflect the retention capability of hydrogel within the dynamic periodontal pocket, a macroscopic adhesion test was performed on human skin and latex surfaces (Fig. 3c). Attributing to the integration of hydrophobic PUE molecules within hydrophilic COL matrix, the PL/PUE/COL hydrogel exhibited robust adhesion to the epithelium tissue and even hydrophobic latex surface. It maintained structural integrity and interfacial bonding during repeated bending of the finger joint, demonstrating exceptional tissue compliance. It allowed PL/PUE/COL hydrogel to accommodate the physiological movements in periodontal pockets caused by mastication and speech without detaching or creating mechanical irritation.

To assess the viscoelastic stability of the hydrogel network under dynamic conditions, oscillatory rheology was performed. As illustrated in Fig. 3d, all hydrogel groups exhibited a characteristic solid-like elastic behavior, with the storage modulus (G’) consistently exceeding the loss modulus (G”) across a wide range of oscillation strains. Differently, the supramolecular structure enhanced network stiffness. The plateau G’ of PL/PUE/COL hydrogel displayed about 2.5-fold increase in contrast of COL hydrogel, suggesting that PUE and PL actively participate in the network assembly and restrict the segmental motion of collagen chains. Meanwhile, PL/PUE/COL exhibited not only high elasticity but also a significantly elevated loss modulus (G”) compared to COL hydrogel. This enhanced viscosity could facilitate energy dissipation at the tissue-material interface, explaining its adhesion ability and resistance against mucosal movement^46^. Interestingly, the “weak strain overshoot” behavior in G” was recognized in the PL/PUE/COL supramolecular hydrogel before the network breakdown. It indicated that non-covalent bonds in supramolecular interactions may be disrupted or rearranged with increasing strain, which resisted deformation and dissipated energy before fracture^47^. This phenomenon confirmed the successful construction of the supramolecular hydrogel at the macroscopic level. We further evaluated the compressive mechanical properties of the hydrogels and obtained the stress-strain curves (Fig. 3e). As shown in Fig. 3f, g, in comparison with COL, the PL/PUE/COL hydrogel increased the compressive strength by ~52% (from 0.103 to 0.157 MPa) and nearly doubled the compressive modulus (from 0.055 to 0.109 MPa). The XRD patterns of PUE/COL and PL/PUE/COL showed no characteristic diffraction peaks of crystalline PUE, consistent with the FTIR results, indicating that PUE was molecularly dispersed within the collagen matrix in an amorphous state, stabilized by the supramolecular interactions (Fig. 3h, i).

The cumulative release profile of PUE from the PL/PUE/COL hydrogel was monitored for 21 days (Fig. 3j). Unlike the rapid diffusion of free small molecules, the hydrogel exhibited a sustained release behavior. The controlled release was attributed to the strong supramolecular interactions between PUE and the biopolymer matrix, which effectively restricted the release of PUE and PUE-based supramolecular complexes.

### Synergistic extracellular antibacterial activity and biocompatibility of PL/PUE/COL hydrogel

In the periodontitis microenvironment, the acidic conditions and the presence of matrix metalloproteinases facilitate the local hydrolysis of the supramolecular complexes in the PL/PUE/COL supramolecular hydrogel, leading to the release of supramolecular complex fragments that exert biological functions locally. Since periodontitis is a chronic disease caused by bacterial infection, we first selected anaerobic *P. gingivalis* as the typical bacterium of periodontitis. To rigorously benchmark the antibacterial efficacy against current clinical standards, minocycline hydrochloride (Mino) was taken as the positive control for comparison^48–50^.

**Fig. 4:**
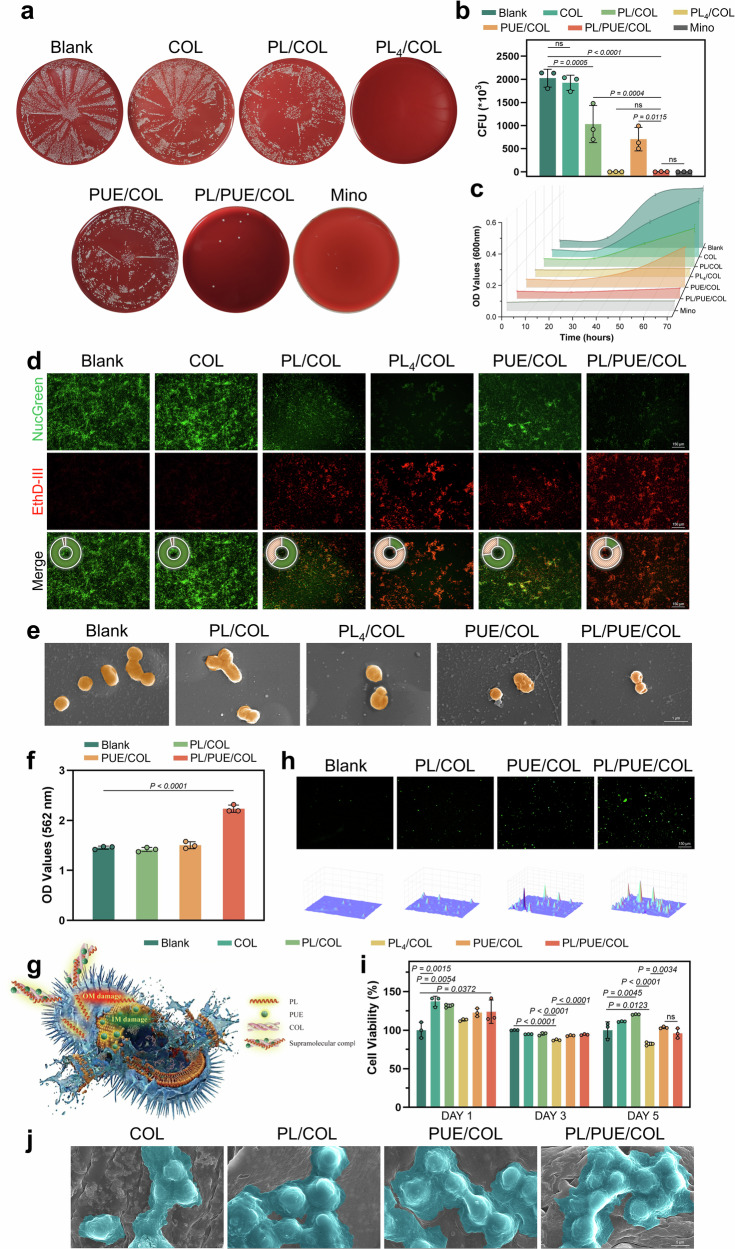
Evaluation of antibacterial capability and biocompatibility. **a** Representative photographs of spread plates of *P. gingivalis* colonies after coculturing with COL, PL/COL, PL_4_/COL, PUE/COL, PL/PUE/COL, and minocycline. **b** Quantification of *P. gingivalis* colonies (*n* = 3 independent biological samples). **c** Growth curves of *P. gingivalis* cultured in BHI media with COL, PL/COL, PL_4_/COL, PUE/COL, PL/PUE/COL, and minocycline over a 72-h period (*n* = 3 independent biological samples). **d** Representative fluorescence images of the treated *P. gingivalis* through Live/Dead staining. The images were representative of three independently repeated experiments from each group. **e** SEM morphologies of *P. gingivalis* incubated without or with different hydrogels. The images were representative of three independently repeated experiments from each group. **f** The leakage of protein from *P. gingivalis* incubated without or with PL/COL, PUE/COL and PL/PUE/COL hydrogels (*n* = 3 independent biological samples). **g** Illustration of the bacteria killing procedure. **h** DiBAC4(3) staining images of *P. gingivalis* incubated without or with PL/COL, PUE/COL and PL/PUE/COL hydrogels. The images were representative of three independently repeated experiments from each group. **i** Cell viability of RAW 264.7 cells after culturing with different hydrogels for 1 day, 3 days, and 5 days (*n* = 3 independent biological samples). **j** SEM morphologies of RAW 264.7 cells after coculturing with COL, PL/COL, PUE/COL, and PL/PUE/COL hydrogels. The images were representative of three independently repeated experiments from each group. All error bars represent mean ± SD. *P* values were calculated by one-way ANOVA followed by Tukey’s post hoc test (ns not significant). PL poly-ε-lysine, PUE puerarin, COL recombinant human type I collagen, BHI brain heart infusion, SEM scanning electron microscopy, DiBAC4(3) bis-(1,3-dibutylbarbituric acid)-trimethine oxonol. Source data are provided as a Source Data file.

Through the spread plate test, PL showed a concentration-dependent bactericidal effect. As shown in Fig. 4a, in the PL_4_/COL group, the incorporation of high-concentration PL (4 mg/mL) exhibited a more significant antibacterial effect compared to the PL/COL group with low concentration of PL (2 mg/mL). PUE/COL hydrogel demonstrated restricted antibacterial effects comparable to PL/COL. Intriguingly, when low-concentration PL was combined with PUE (PL/PUE/COL), a synergistic antibacterial effect was observed, reaching the efficient antibacterial level of PL_4_/COL and Mino positive control. The colony-forming unit (CFU) counts were quantified to evaluate the antibacterial efficiency (Fig. 4b). While the PL/COL and PUE/COL treatments only partially decreased the viable bacterial counts compared to the Blank and COL groups, the PL_4_/COL, PL/PUE/COL, and Mino groups effectively eradicated the bacteria, reducing the CFU counts by ~1000-fold. The corresponding calculated antibacterial rates are provided in Supplementary Fig. 11, confirming that the PL_4_/COL, PL/PUE/COL, and Mino groups achieved an antibacterial rate close to 100%, nearly twofold higher than those of the PL/COL and PUE/COL groups. To comprehensively assess the effect of supramolecular hydrogel on bacterial growth, we monitored bacterial growth over a 72-h period (Fig. 4c). In the Blank and COL groups, bacterial growth began with rapid proliferation at 24 h, which slowed down after 48 h. The PL/COL and PUE/COL groups appeared to slightly inhibit bacterial proliferation, while PL/PUE/COL, PL_4_/COL, and Mino groups significantly suppressed bacterial proliferation, with no noticeable bacterial growth observed throughout the entire 72-h period. Live/dead assay was also conducted to visualize the viable (green) and non-viable (red) *P. gingivalis* (Fig. 4d). Blank and COL groups were almost entirely dyed green, which means few bacteria were killed. Consistent with previous results, a majority of the bacteria in PL/PUE/COL and PL_4_/COL groups were killed, while only part of the bacteria exhibited red fluorescence in PL/COL and PUE/COL groups. SEM was used to investigate the micromorphology of *P. gingivalis* under different treatments (Fig. 4e). *P. gingivalis* exhibited spherical or capsule-like morphology with a smooth surface in the Blank group. In the other groups, bacteria exhibited varying degrees of morphological changes, with the most pronounced shrinkage deformation observed in the PL/PUE/COL group, indicating destruction of bacterial structures. To further validate the antibacterial performance of the supramolecular hydrogels against the pathogen in periodontitis, we evaluated their inhibitory effect against *Fusobacterium nucleatum* (*F. nucleatum*). *F. nucleatum* can facilitate coaggregation between early and late colonizers in dental plaque, enhance the infectivity of other periodontitis pathogens and directly influence host responses^51,52^. We monitored *F. nucleatum* growth over a 72-hour period (Supplementary Fig. 12). The PL/COL and PUE/COL groups showed limited impact on bacterial expansion, whereas the PL/PUE/COL group effectively restrained bacterial growth, with virtually no detectable increase throughout the entire observation window. For Live/Dead staining, the PL/COL and PUE/COL groups exhibited predominantly green fluorescence with only limited red staining, while PL/PUE/COL displayed strong red signals (Supplementary Fig. 13). This confirms the bactericidal activity of the PL/PUE/COL supramolecular complex against *F. nucleatum*. Since the periodontitis microenvironment is typically characterized by complex polymicrobial communities rather than a single pathogen, we further evaluated the antibacterial ability against an in vitro dual-species infection model comprising *P. gingivalis* and *F. nucleatum*. As shown in Supplementary Fig. 14, the Blank and COL groups exhibited rapid and continuous bacterial proliferation. The PL/COL and PUE/COL hydrogels provided only limited inhibition against the polymicrobial challenge. In contrast, the PL/PUE/COL hydrogel effectively suppressed the growth of the mixed bacterial population throughout the 72-h period. Its efficacy was close to both the high-dose PL_4_/COL and the clinical standard Mino group. It indicates that the synergistic antibacterial capacity remains satisfactory even in the complex dual-species infection microenvironment.

Having confirmed that the synergistic antibacterial efficacy of the PL/PUE/COL system was comparable to that of the PL_4_/COL and Mino group, we next tried to elucidate the mechanistic basis of this synergistic antibacterial effect. We therefore focused our mechanistic investigations on the three core experimental groups (PL/COL, PUE/COL, and PL/PUE/COL), and first evaluated the leakage of intracellular contents from *P. gingivalis*. Quantitative analysis revealed that the supernatants of the PL/PUE/COL group had significantly higher levels of protein content leaked from bacterial cytoplasm, while PUE/COL group and PL/COL group were at the same level as Blank group (Fig. 4f). It indicated that the supramolecular complexes released from PL/PUE/COL synergistically damaged the bacterial structure, leading to a significant leakage of intracellular content.

The synergistic effect observed in protein leakage can be explained by the complementary antibacterial mechanisms of PL and PUE. *P. gingivalis* is a Gram-negative bacterium, and its cell envelope primarily consists of the outer membrane (OM) and the inner membrane (IM)^53,54^. The OM is a lipid bilayer, principally consisting of lipopolysaccharide (LPS), which is an effective barrier for hydrophobic molecules. The IM is a phospholipid bilayer, which is a key site for energy generation and metabolic processes. Cationic PL interacts with the negatively charged cell surface, stripping the LPS layer and leading to a permeabilization of the OM^19^. However, as a macromolecule, PL cannot easily penetrate the IM. PUE in the supramolecular complex is also enriched on the bacterial surface due to the interaction between PL and the bacteria. PUE is an amphiphilic flavonoid molecule, capable of interacting with the IM^55,56^. In the absence of cholesterol, the IM is susceptible to the insertion of PUE, which leads to the disturbance of the structure and function of IM^57,58^. In the supramolecular system, we hypothesize that PL first disrupts the OM barrier. PUE then accumulates in the IM and disturbs the phospholipid bilayer. This combined action disrupts both the OM and IM, resulting in the significant leakage observed (Fig. 4g).

To further validate this hypothesis, we assessed the bacterial transmembrane potential using the voltage-sensitive dye bis-(1,3-dibutylbarbituric acid)-trimethine oxonol (DiBAC4(3)) (Fig. 4h). The PL/PUE/COL group exhibited intense green fluorescence, indicative of severe membrane depolarization. The PUE/COL group and PL/COL group displayed only faint signals, while the Blank group showed negligible fluorescence. Compared to the PL/COL and PUE/COL groups, the PL/PUE/COL group exhibited a significantly higher mean fluorescence intensity (Supplementary Fig. 15). This can be explained by the specific localization of the electron transport chain on the IM. In the process, PL disrupts the OM barrier, thereby enabling PUE to effectively perturb the metabolic activity of the IM.

While both PL_4_/COL and PL/PUE/COL both achieved high bactericidal efficacy, the clinical translation potential of high-concentration cationic antimicrobial polypeptides is often limited by potential biosafety risks. We therefore systematically evaluated the in vitro biocompatibility of all hydrogels to identify the optimal therapeutic formulation with both high antibacterial activity and favorable biocompatibility. In the Cell Counting Kit-8 (CCK-8) assay, all groups exhibited excellent biocompatibility on day 1. By day 3, except for the PL_4_/COL group, all other groups maintained high cell viability comparable to the Blank group. For the proliferative activity on day 5, cell viability in the PL_4_/COL group declined further, whereas the other groups retained favorable viability (Fig. 4i). Live/Dead staining assay exhibited similar results (Supplementary Fig. 16). A notable number of dead cells were observed in the PL_4_/COL group, while only sporadic dead cells were detected in the other groups. Hemolysis assay was performed to evaluate the blood compatibility of hydrogels (Supplementary Fig. 17). The hemolysis rate (HR) values of all groups were lower than the international permissible level of 5% for biomedical materials, indicating the nonhemolytic properties of the hydrogels. Morphologically, macrophages on COL, PL/COL, PUE/COL, and PL/PUE/COL exhibited well-spread morphology and the formation of lamellipodia, indicating enhanced cell activity and adhesive behavior (Fig. 4j). These results indicated that while both PL_4_/COL and PL/PUE/COL possessed excellent antibacterial efficacy, PL_4_/COL induced mild cytotoxicity.

Notably, the cell viability of the PL/COL group was comparable to that of the PL/PUE/COL group, indicating that the reduced toxicity was primarily due to the lower PL concentration, and the incorporation of PUE did not further alter the cytotoxicity. Therefore, as the synergistic supramolecular complex of PL and PUE not only enhances antibacterial efficiency but also ensures biocompatibility, we used the PL with a concentration of 2 mg/mL in the following experiments.

### Immunomodulatory effects of PL/PUE/COL hydrogel

The bacterial toxins, such as LPS and inflammatory mediators in the periodontitis microenvironment, result in immune dysregulation and bone resorption^59^. Specifically, the microenvironment drives macrophage polarization toward the pro-inflammatory M1 phenotype, which exacerbates periodontal tissue destruction through the excessive secretion of inflammatory cytokines. In contrast, M2 phenotype contributes to tissue repair by secreting anti-inflammatory and reparative signals^60^. Here, we used LPS to simulate the periodontitis microenvironment and investigate the immunoregulatory effect of supramolecular hydrogels on the periodontitis microenvironment.

**Fig. 5:**
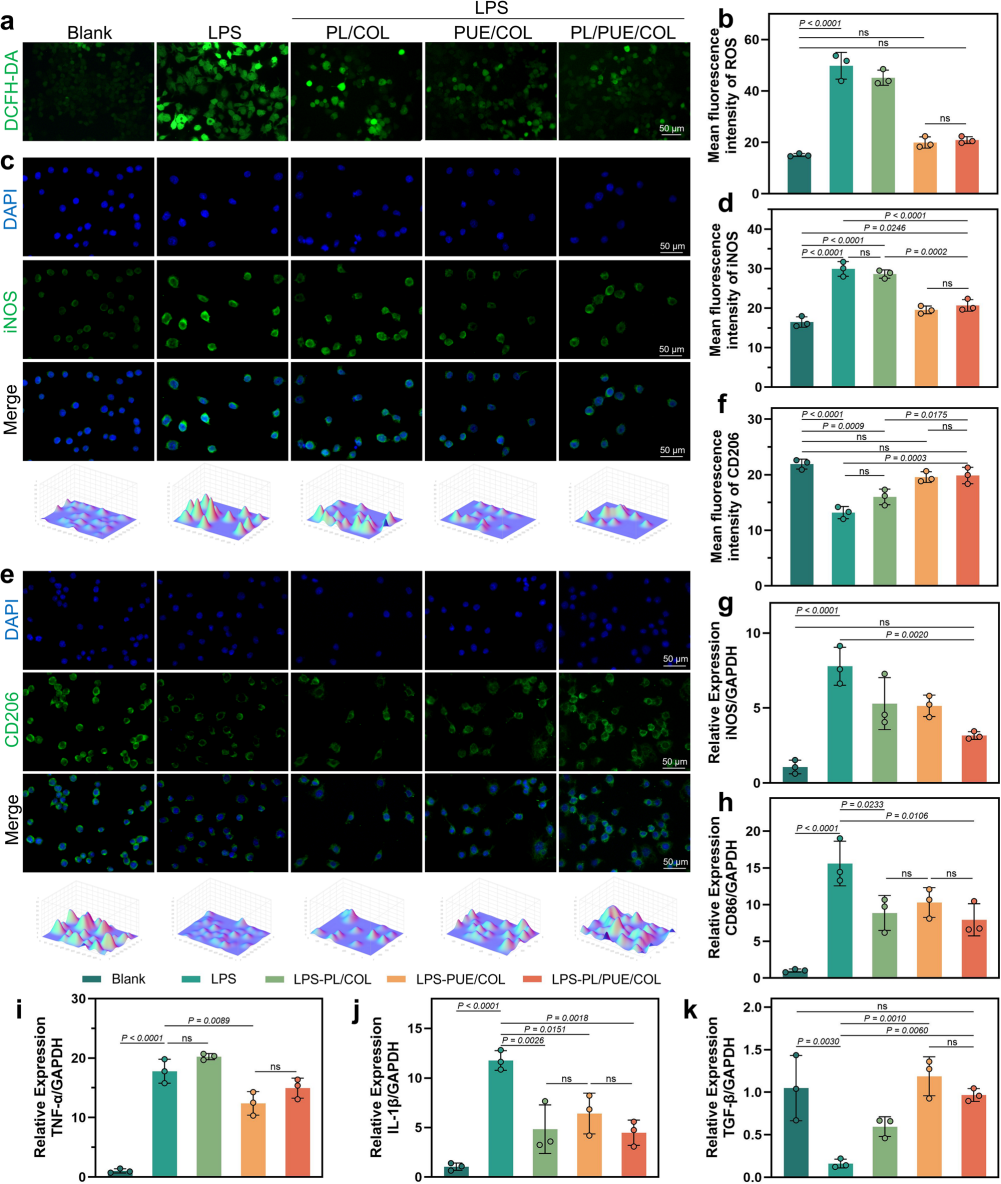
Cellular antioxidation and anti-inflammatory properties. **a** Representative fluorescence images of intracellular ROS detected by DCFH-DA, treated without or with PL/COL, PUE/COL and PL/PUE/COL hydrogels. **b** Statistical analysis of mean fluorescence intensity in Fig. 5a (*n* = 3 independent biological samples). **c** Representative fluorescence images of the expression of iNOS in RAW 264.7 cells with different treatments and **d** related statistical results of mean fluorescence intensity (*n* = 3 independent biological samples). **e** Representative fluorescence images of the expression of CD206 in RAW 264.7 cells with different treatments and **f** related statistical results of mean fluorescence intensity (*n* = 3 independent biological samples). Relative mRNA expression of **g** *iNOS*, **h** *CD86*, **i** *TNF-α*, **j** *IL-1β*, and **k** *TGF-β* in RAW 264.7 cells with different treatments (*n* = 3 independent biological samples). All error bars represent mean ± SD. *P* values were calculated by one-way ANOVA followed by Tukey’s post hoc test (ns not significant). PL poly-ε-lysine, PUE puerarin, COL recombinant human type I collagen, ROS reactive oxygen species, DCFH-DA 2’,7’-dichlorofluorescin diacetate, iNOS inducible nitric oxide synthase, TNF-α tumor necrosis factor-α, IL-1β interleukin-1β, TGF-β transforming growth factor-β. Source data are provided as a Source Data file.

Excess ROS can drive M1 polarization, leading to an imbalance in the M1/M2 ratio. Thus, 2’,7’-dichlorofluorescin diacetate (DCFH-DA) was used to demonstrate the cellular ROS-scavenging property. As shown in Fig. 5a, b, RAW 264.7 cells exhibited intense fluorescence upon LPS stimulation, indicating a significant elevation in intracellular ROS level. When cocultured with PUE/COL and PL/PUE/COL hydrogels under LPS stimulation, the mean fluorescent intensity was significantly attenuated, while the PL/COL group showed no significant difference with the LPS group. It suggests the strong ability of PL/PUE/COL supramolecular hydrogel to scavenge ROS due to the antioxidant activity of PUE, as previously reported^61^. Immunofluorescence staining was then used to assess the effect of supramolecular hydrogels on M1/M2 polarization in macrophages. LPS stimulation remarkably upregulated inducible nitric oxide synthase (iNOS, a marker of M1 macrophages) expression, and the treatment with PUE/COL and PL/PUE/COL effectively downregulated these levels (Fig. 5c). No significant difference was observed between the PL/COL group and the LPS group. While the PUE/COL and PL/PUE/COL groups exhibited significantly lower iNOS expression compared to the LPS group, there was no statistical difference between these two treatment groups (Fig. 5d). Conversely, while LPS stimulation downregulated the expression of CD206 (a marker of M2 macrophages), PUE/COL and PL/PUE/COL restored its expression to a level close to that of the Blank group (Fig. 5e, f).

Furthermore, at the mRNA level, the M1 phenotypic markers (*iNOS* and *CD86*) expressions were downregulated in the PL/PUE/COL group following LPS stimulation, corroborating the ability to inhibit M1 polarization (Fig. 5g, h). Similarly, the PUE-based supramolecular hydrogels attenuated the LPS-induced upregulation of inflammatory cytokines, with significant inhibition observed for interleukin-1β (*IL-1β*), and a notable downward trend was also seen in tumor necrosis factor-α (*TNF-α*) expression (Fig. 5i, j). The PL/COL group also downregulated the expression levels of *CD86* and *IL-1β* to varying degrees, which may be attributed to its cationic characteristic to neutralize LPS extracellularly, reducing the initial transcriptional signaling. Similarly, LPS downregulated the expression of transforming growth factor-β (*TGF-β*), a cytokine associated with tissue repair. This reduction was significantly restored following treatment with PUE/COL or PL/PUE/COL (Fig. 5k).

Collectively, these results demonstrate that the PL/PUE/COL supramolecular hydrogel effectively scavenges ROS, inhibits M1 polarization, and promotes M2 polarization, laying the foundation for remodeling the immune microenvironment of periodontitis.

### Intracellular bacterial eradication and restoration of osteo-immune microenvironment

While alleviating extracellular LPS-induced inflammation is crucial, periodontitis pathogens such as *P. gingivalis* can actively invade host cells to evade immune clearance, disrupting local immune and metabolic responses. As the first line of innate defense, macrophages play a pivotal role in eradicating these pathogens. Therefore, beyond general immunomodulation, enhancing the capacity of macrophages to clear intracellular bacteria is essential to overcome immune evasion and restore the osteo-immune microenvironment.

To evaluate the engulfment ability, the external uptake of macrophages was tested using RAW 264.7 cells cocultured with fluorescein isothiocyanate (FITC)-labeled polystyrene microspheres. As shown in Supplementary Fig. 18a, the inclusion of PUE significantly promoted the phagocytic efficiency in PUE/COL and PL/PUE/COL groups relative to the Blank group. Quantitative results indicated no significant difference between the PUE/COL and PL/PUE/COL groups (Supplementary Fig. 18b). Similarly, the PL/COL group showed no statistical difference compared to the Blank group. It suggested that PUE-based supramolecular complexes elevated the engulfment ability of macrophages.

**Fig. 6:**
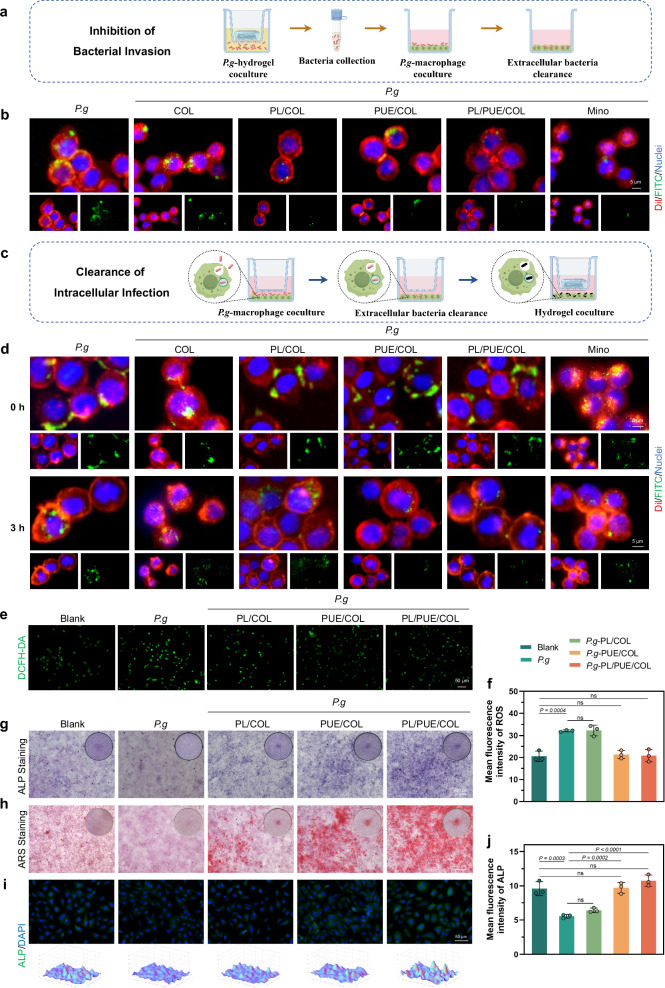
Evaluation of phagocytic capability, intracellular bactericidal efficacy, and immune-mediated osteogenesis. **a** Schematic of the bacterial invasion assay. Created by Figdraw (www.figdraw.com). **b** Representative fluorescence images of the bacterial invasion assay. The images were representative of three independently repeated experiments from each group. **c** Schematic of the intracellular bacterial clearance assay. Created by Figdraw (www.figdraw.com). **d** Representative fluorescence images of intracellular FITC-labeled *P. gingivalis* before (top) and after different treatments for 3 h (bottom). The images were representative of three independently repeated experiments from each group. **e** Representative fluorescence images and **f** statistical analysis of intracellular ROS in *P. gingivalis* infected RAW 264.7 cells with treatments of PL/COL, PUE/COL, and PL/PUE/COL hydrogels (*n* = 3 independent biological samples). **g** ALP and **h** ARS staining of MC3T3-E1 cells cultured with conditioned medium from infected RAW 264.7 with different treatments. The images were representative of three independently repeated experiments from each group. **i** Representative fluorescence images and **j** statistical analysis of the expression of ALP in MC3T3-E1 cells cultured with conditioned medium from infected RAW 264.7 with different treatments (*n* = 3 independent biological samples). All error bars represent mean ± SD. *P* values were calculated by one-way ANOVA followed by Tukey’s post hoc test (ns not significant). PL poly-ε-lysine, PUE puerarin, COL recombinant human type I collagen, FITC fluorescein isothiocyanate, ROS reactive oxygen species, ALP alkaline phosphatase, ARS Alizarin Red S. Source data are provided as a Source Data file.

However, due to the immune evasion of *P. gingivalis*, robust phagocytosis alone is insufficient. Enhanced intracellular eradication is essential to prevent bacterial persistence. We further evaluated the capacity of the supramolecular hydrogel to both eliminate intracellular bacteria and attenuate bacterial invasion, using Mino as the positive control at the same concentration based on its robust in vitro antibacterial performance (Fig. 4a–c). As illustrated in Fig. 6a, to investigate the ability of hydrogels to attenuate bacterial invasion, bacteria were treated with hydrogels before being cocultured with macrophages. Compared with the untreated *P. gingivalis* (*P.g*) group, the COL group and PUE/COL group, the PL/COL group, PL/PUE/COL group, and the Mino group reduced the bacterial internalization (Fig. 6b). It may be attributed to the PL-induced and antibiotic-induced disruption of the *P. gingivalis* OM, which impairs its intracellular persistence capacity. Furthermore, under the synergistic action of PUE and PL, the disruption of the electron transport chain on IM hindered ATP production, which further reduced bacterial invasive activity (Supplementary Fig. 19).

To investigate the capacity of the supramolecular hydrogel to eliminate intracellular bacteria, as illustrated in Fig. 6c, RAW 264.7 cells were infected by *P. gingivalis* before hydrogel treatment. Macrophages were infected with FITC-labeled *P. gingivalis* for 3 h. After removing extracellular bacteria and staining macrophage membranes with Dil membrane dye, immediate observation confirmed that the initial bacterial internalization was comparable among all groups. Subsequently, the infected macrophages were cocultured with the different hydrogels for another 3 h. Compared to the untreated *P. gingivalis* (*P.g*) group, the other groups all exhibited reduced intracellular bacterial persistence to varying degrees (Fig. 6d). However, despite its superior extracellular bactericidal efficacy, the Mino positive control displayed limited intracellular eradication efficiency, which was comparable to the PL/COL group. In contrast, the PUE/COL and PL/PUE/COL groups demonstrated significant clearance, evidenced by sporadic intracellular fluorescence signals. These findings indicated that the PUE-based supramolecular complexes effectively cleared the majority of intracellular *P. gingivalis*.

The presence of persistent intracellular bacteria induced a sustained pro-inflammatory response, driving the secretion of cytokines and the generation of excessive ROS^62,63^. As shown in Fig. 6e, invasion by *P. gingivalis* significantly elevated intracellular ROS levels. Quantitative analysis revealed that while PL/COL treatment had no significant effect, both PUE/COL and PL/PUE/COL treatments significantly reduced intracellular ROS (Fig. 6f). Macrophages play an important role in the regeneration and repair of periodontal tissues. Immune dysregulation hinders stem cell recruitment and osteogenic differentiation, thereby impairing tissue repair^59^. Thus, osteoblasts were indirectly cocultured with macrophages, to evaluate the impact of *P. gingivalis* on macrophage-mediated osteogenesis. The early and late osteogenic activities were assessed utilizing alkaline phosphatase (ALP) and Alizarin Red S (ARS) staining. Representative ALP staining images on day 7 revealed that *P. gingivalis* suppressed macrophage-mediated early osteogenic differentiation, which was reversed by the treatment of PUE/COL and PL/PUE/COL (Fig. 6g). In ARS staining on day 14 for calcium nodules evaluation, the rescue effect was even more pronounced (Fig. 6h). While the Blank group exhibited relatively modest osteogenic activity, PUE/COL and PL/PUE/COL groups exhibited mineralization levels that even surpassed the Blank group. PL/COL also showed enhanced calcium nodules. Next, we utilized immunofluorescence staining to examine the macrophage-mediated osteogenic differentiation at the protein levels (Fig. 6i, j). It revealed that *P. gingivalis* treatment downregulated ALP expression in MC3T3-E1, which was restored in the PUE/COL and PL/PUE/COL groups. These findings indicated that the PUE-based supramolecular complexes possess superior immunomodulatory capabilities, simultaneously eliminating intracellular bacteria and potentiating macrophage-mediated bone tissue repair.

Apart from immune-mediated indirect osteogenesis, we evaluated the direct osteogenic effects of the hydrogels on osteoblasts. As shown in Supplementary Fig. 20, extracts from PUE/COL and PL/PUE/COL enhanced ALP staining intensity and protein expression compared to the Blank group, whereas the PL/COL group showed only a slight improvement. It suggests that in addition to the immunomodulatory role, PUE also directly promotes osteogenic differentiation, thereby facilitating local bone repair through a multidimensional therapeutic approach.

### Mechanism of reversing immuno-metabolic dysregulation

Previous studies have demonstrated that PUE exhibited favorable antioxidant and immunomodulatory properties, while also regulating lysosomal activity^23,25^. However, the polar-biased amphiphilic structure of PUE limited its transmembrane transport, making it difficult to effectively exert intracellular functions. In this study, we utilized PUE-based supramolecular complexes as “Trojan horses” to enhance intracellular transport through endocytosis, thereby effectively reversing immuno-metabolic dysregulation in the periodontitis microenvironment. To investigate the mechanism underlying the immuno-metabolic regulation of the supramolecular complex, we performed RNA sequencing on macrophages with and without *P. gingivalis* infection, and those treated with supramolecular hydrogels post-infection.

**Fig. 7:**
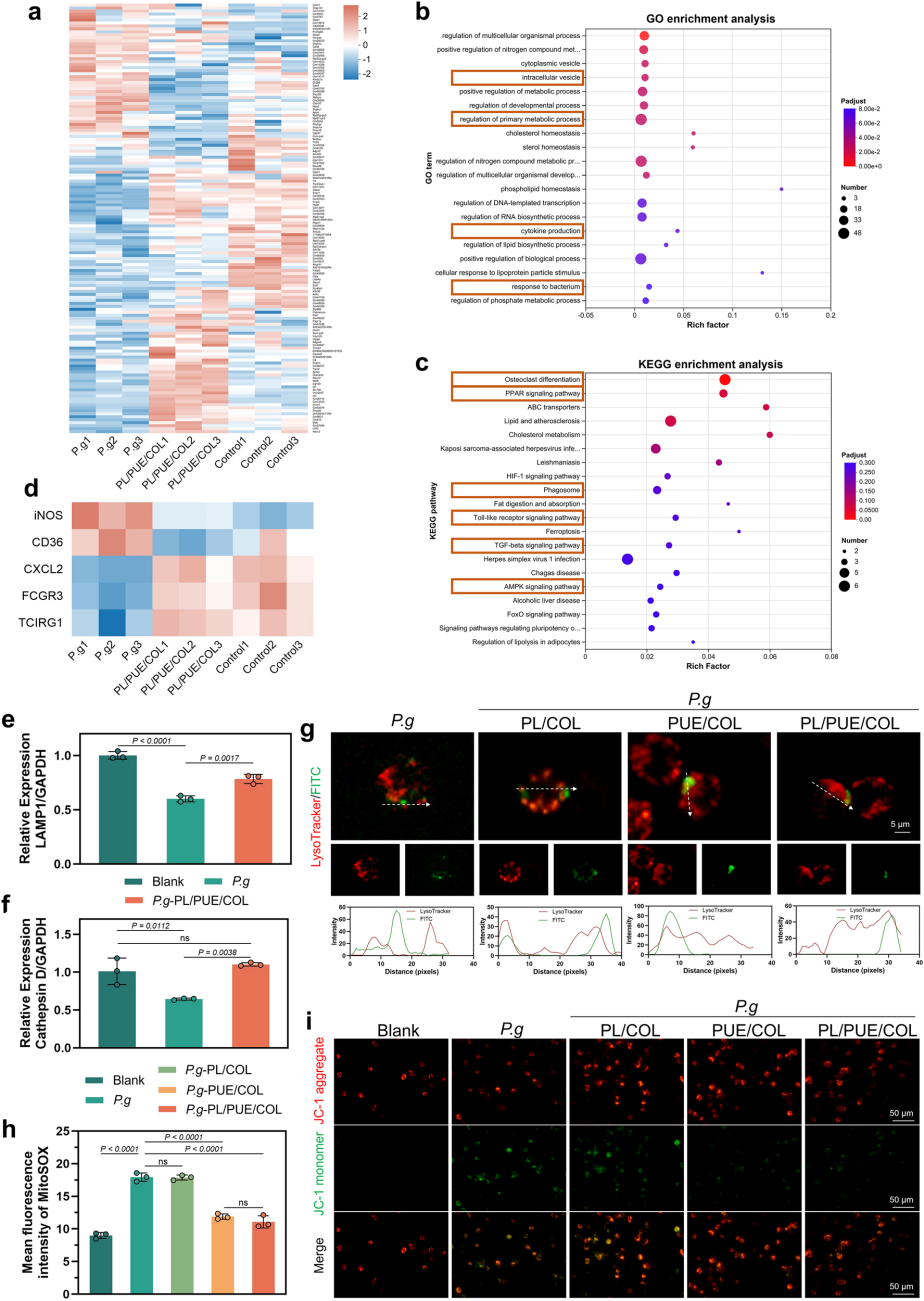
Mechanism of PL/PUE/COL-mediated immuno-metabolic regulation. **a** Heat map of significantly upregulated and downregulated genes. **b** GO enrichment analysis between *P. gingivalis* group and PL/PUE/COL group. **c** KEGG pathway enrichment analysis between *P. gingivalis* group and PL/PUE/COL group. **d** Heat map of genes related to inflammation response and lysosomal function. Relative mRNA expression of **e** *LAMP1* and **f** *CTSD* in infected RAW 264.7 cells with different treatments (*n* = 3 independent biological samples). **g** Colocalization images of lysosomes and bacteria. **h** Quantitative analysis of the mtROS in infected RAW 264.7 cells with different treatments (*n* = 3 independent biological samples). **i** Representative fluorescence images of MMP JC-1 staining. The images were representative of three independently repeated experiments from each group. All error bars represent mean ± SD. *P* values were calculated by one-way ANOVA followed by Tukey’s post hoc test (ns not significant). PL poly-ε-lysine, PUE puerarin, COL recombinant human type I collagen, GO gene ontology, KEGG Kyoto Encyclopedia of Genes and Genomes, LAMP1 lysosomal-associated membrane protein 1, CTSD cathepsin D, mtROS mitochondrial reactive oxygen species, MMP mitochondrial membrane potential. Source data are provided as a Source Data file.

Three groups showed different transcriptomic profiles, while PL/PUE/COL treated group displayed expression profiles more similar to those of the uninfected control (Blank) group (Fig. 7a). To reveal the functions of the differentially expressed genes (DEG), gene ontology (GO) analysis was performed. As shown in Fig. 7b, the DEG were highly correlated with intracellular vesicles and response to bacterium, which are closely related to the processes of bacteria invasion. Additionally, GO analysis also revealed gene enrichment related to cytokine production and the regulation of primary metabolic processes. It further elucidated the role of PL/PUE/COL in promoting macrophage-mediated osteogenic activity (Fig. 6g–j). Furthermore, Kyoto Encyclopedia of Genes and Genomes (KEGG) enrichment analysis revealed that the treatment of supramolecular hydrogel significantly affected the phagosome pathway, which confirmed the enhanced phagocytic and lysosomal digestive capacity after PL/PUE/COL treatment (Fig. 7c). KEGG analysis also found that DEG were enriched in *TGF-β*, adenosine 5’-monophosphate-activated protein kinase (AMPK), Toll-like receptor and peroxisome proliferator-activated receptors (PPAR) signaling pathway. It indicated the immuno-metabolic regulation ability of the PL/PUE/COL supramolecular hydrogel. It is of particular significance that the Toll-like receptor signaling pathway is closely related to immune evasion by periodontal bacteria and their intracellular persistence^7,10,63^.

The representative DEG related to inflammation response and lysosomal function were exhibited in the heat map (Fig. 7d). The invasion of *P. gingivalis* increased the expression of pro-inflammatory genes such as *iNOS* and *CD36*. The upregulation of *iNOS* in macrophages can lead to the production of large amounts of NO, resulting in oxidative stress and tissue damage^64^. The pattern recognition receptor CD36 is able to amplify the intracellular inflammatory signals induced by bacteria^65^. The application of PL/PUE/COL reversed the expression of these inflammatory mediators, thereby alleviating the hyperinflammatory response triggered by bacterial infection. *P. gingivalis* also downregulated the expression of *CXCL2*, inhibiting the chemotaxis of immune cells^66^. In contrast, PL/PUE/COL restored appropriately chemotactic activity, thereby activating the immune clearance capacity in the infectious microenvironment. Importantly, PL/PUE/COL also reversed the downregulation of Fc gamma receptor III (FCGR3; phagocytic receptor-related gene) and T-cell immune regulator 1 (TCIRG1; lysosomal activity-related gene) caused by *P. gingivalis* infection, rescuing the induced immune subversion. Therein, *TCIRG1* encodes a key subunit of the vacuolar-type H^+^-ATPase (V-ATPase) complex, the primary proton pump essential for maintaining lysosomal acidity^67^. Its downregulation by *P. gingivalis* implies a mechanism of immune subversion via inhibiting lysosomal acidification^11,68^. Therefore, the upregulation of *TCIRG1* under PL/PUE/COL treatment indicated the restoration of lysosomal activity.

In this view, we hypothesized that the PL/PUE/COL hydrogel could revive lysosomal function to eradicate intracellular *P. gingivalis* and influence metabolic behaviors. To validate this hypothesis, we further detected the mRNA expression levels of lysosomal-associated membrane protein 1 (*LAMP1*) and cathepsin D (*CTSD*) via real-time quantitative polymerase chain reaction (PCR), which reflected lysosomal homeostasis and function. As a marker of lysosomal structural integrity, *LAMP1* exhibited downregulation following *P. gingivalis* infection, and it was significantly restored after treatment with the PL/PUE/COL supramolecular hydrogels (Fig. 7e). Similarly, CTSD, an important lysosomal enzyme involved in bacterial degradation, was downregulated following *P. gingivalis* infection, and this reduction was significantly restored after PL/PUE/COL treatment (Fig. 7f). These results indicated that the PL/PUE/COL supramolecular hydrogel not only restored the V-ATPase complex, but also promoted the homeostasis and enzymatic capacity of lysosome, enhancing the capacity for effective bacterial clearance.

Subsequently, we investigated whether it translated to effective phagolysosomal fusion at the cellular level. We visualized the spatial relationship between intracellular FITC-labeled *P. gingivalis* and lysosomes stained with LysoTracker Red (Fig. 7g). To validate the colocalization, spatial fluorescence intensity profiles were plotted along the paths indicated by the white dashed arrows. In the untreated *P. gingivalis* infection group (*P.g* group) and the PL/COL group, the fluorescence peaks of bacteria (green) and lysosomes (red) were spatially mismatched. Since LysoTracker selectively accumulates in acidic compartments, this separation indicated that *P. gingivalis* evaded phagolysosome fusion or impaired the acidification process. In contrast, after treatment with PUE/COL and PL/PUE/COL, the fluorescence intensity profiles of both channels exhibited a highly synchronized spatial overlap, suggesting a restoration of phagolysosome structure and function. Crucially, since LysoTracker selectively accumulates in highly acidic compartments, its spatial colocalization with the bacteria indicated restored lysosomal acidification. Combined with the molecular upregulation of essential lysosomal proteases (CTSD) and structural membrane proteins (LAMP1), these findings confirm that the PUE-based supramolecular hydrogel revives the lysosomal bioactivity.

The restoration of lysosomal function is intrinsically linked to the cellular redox state, as oxidative stress is a primary driver of lysosomal membrane destabilization. We have demonstrated that *P. gingivalis* infection increased intracellular ROS levels (Fig. 6e). As shown in Fig. 7h and Supplementary Fig. 21, we further evaluated the mitochondrial ROS (mtROS) levels. *P. gingivalis* infection triggered a surge in mitochondrial superoxide production, while PUE/COL and PL/PUE/COL groups exhibited a noteworthy decrease. Under oxidative stress conditions, the infection group displayed a significant increase in mitochondrial membrane potential (MMP) depolarization. Similarly, PUE-based supramolecular hydrogel treatment reversed the change, alleviating cellular oxidative damage (Fig. 7i).

Based on these findings, we proposed a lysosome-centered immuno-metabolic regulatory mechanism. PUE was internalized via supramolecular-mediated endocytosis and subsequently accumulated within the phagolysosomes. While COL and PL were gradually degraded by enzymes, the C-glycoside structure of PUE conferred exceptional resistance to acid hydrolysis and enzymatic degradation. It allowed PUE to accumulate and persist within the phagolysosomes as an in situ antioxidant component.

Typically, macrophages rely on ROS for bactericidal activity. However, *P. gingivalis* utilizes innate antioxidant defenses to resist the physiological ROS generated by macrophages^69^. Rather than being cleared, intracellular pathogens can actively induce excessive, pathological ROS to promote their intracellular survival^70,71^. This excessive ROS generation directly reduces lysosome membrane stability and triggers lysosomal membrane permeabilization (LMP)^72^. LMP usually causes the loss of bactericidal hydrolases, enabling the pathogen to evade lysosomal degradation and persist intracellularly, while simultaneously serving as a principal driver for macrophage necroptosis^70,71^.

By scavenging this pathological ROS burst, PUE attenuates LMP and potentially restrains ROS-driven necroptosis. Preserving lysosomal membrane integrity supports V-ATPase-mediated maintenance of the acidic pH required for hydrolase activation, while stabilized lysosomal homeostasis further interrupts the LMP-triggered cascade of intracellular metabolic dysregulation^72^. Consequently, the supramolecular functional integration not only facilitated intracellular bacteria elimination, but more importantly, reversed immuno-metabolic dysregulation via the restoration of lysosomal homeostasis, thereby establishing a microenvironment conducive to tissue regeneration.

### PL/PUE/COL for in vivo periodontitis treatment

**Fig. 8:**
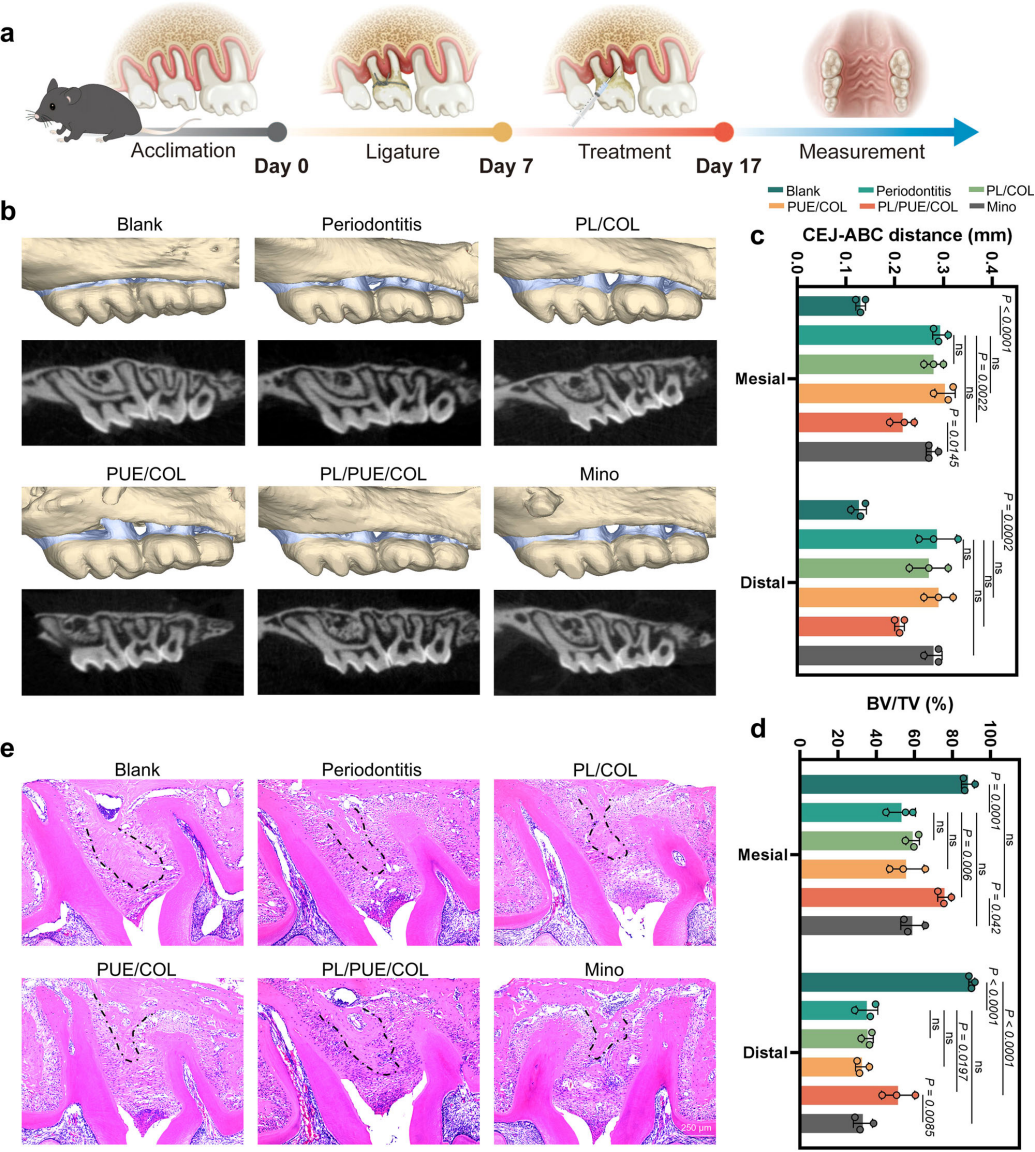
Therapeutic effect of PL/PUE/COL supramolecular hydrogel in periodontitis. **a** Schematic illustration of establishing the ligature-induced periodontitis model. **b** Three-dimensional reconstructed and section images of the maxillary molars with different treatments by Micro-CT. Blue regions represent the distance between ABC and CEJ. Quantitative analysis of **c** CEJ-ABC distance and **d** BV/TV at the mesial and distal aspects of M2 (*n* = 3 independent biological samples). **e** HE staining of the area between the maxillary first and second molars. Dashed lines indicate the alveolar bone within the lesion area. The images were representative of three independently repeated experiments from each group. All error bars represent mean ± SD. *P* values were calculated by one-way ANOVA followed by Tukey’s post hoc test (ns not significant). PL poly-ε-lysine, PUE puerarin, COL recombinant human type I collagen, Micro-CT micro-computed tomography, ABC alveolar bone crest, CEJ cemento-enamel junction, BV/TV bone volume fraction, M2 maxillary second molar, HE hematoxylin and eosin. Source data are provided as a Source Data file.

To evaluate the in vivo therapeutic efficacy of a supramolecular hydrogel, a ligature-induced periodontitis model was established in C57BL/6 mice. Building upon the in vitro synergy between PL and PUE, we focused on the therapeutic performance of the PL/COL, PUE/COL and PL/PUE/COL supramolecular hydrogel in vivo, incorporating the clinical gold standard antibiotic Mino as a positive control. Following the process illustrated in Fig. 8a, hydrogels or Mino were locally injected into the periodontal pockets. Micro-computed tomography (Micro-CT) was employed to visualize and quantify the alveolar bone resorption. Compared to the Blank group, the Periodontitis group exhibited significant alveolar bone loss around the maxillary second molar (M2). The distance between the cemento-enamel junction and alveolar bone crest (CEJ-ABC) notably increased, confirming the successful establishment of the periodontitis model. Treatments with the single-component hydrogels (PL/COL and PUE/COL) and the Mino failed to effectively arrest the bone resorption, exhibiting severe alveolar bone loss comparable to the untreated Periodontitis group. In contrast, mice treated with PL/PUE/COL showed improved alveolar bone recovery, as exhibited in the three-dimensional reconstructed and cross-sectional view (Fig. 8b). Measurements of the CEJ-ABC distance were conducted at both the mesial and distal aspects of M2. While the PL/COL, PUE/COL, and Mino groups maintained large CEJ-ABC distances similar to the Periodontitis group, the PL/PUE/COL treated group showed a significant reduction at the mesial aspect, with a consistent downward trend also observed at the distal aspect, indicating the preservation of alveolar bone height (Fig. 8c). To further characterize bone structure, bone volume fraction (BV/TV) was quantified at both the mesial and distal aspects. The Blank group exhibited the highest BV/TV values, reflecting the most intact alveolar bone structure. The Periodontitis group had the lowest BV/TV values, which indicated a significant reduction in alveolar bone mass following periodontitis. Similarly, the PL/COL, PUE/COL, and Mino groups maintained low BV/TV levels, while PL/PUE/COL group showed an improvement in BV/TV values (Fig. 8d). The quantified values were consistent with the results of three-dimensional reconstruction images, thereby confirming the PL/PUE/COL supramolecular hydrogel had a significant advantage in addressing alveolar bone destruction in periodontitis, which is consistent with its in vitro antibacterial activity and immuno-metabolic regulation capabilities.

Histological evaluation via hematoxylin and eosin (HE) staining revealed that the Periodontitis group exhibited pronounced pathological characteristics, including inflammatory cell infiltration, apical migration of the junctional epithelium, and alveolar ridge resorption. The destructive phenotypes were also observed in the PL/COL, PUE/COL, and Mino-treated groups, indicating their limited therapeutic efficacy. In comparison, the PL/PUE/COL group demonstrated effective tissue restoration with reduced inflammation infiltration and the re-establishment of junctional epithelium and alveolar bone (Fig. 8e). To further evaluate the periodontal tissues, Masson staining was performed to assess collagen deposition and organization (Supplementary Fig. 22). The Blank group displayed an intact periodontal tissue structure, with collagen fibers arranged in an orderly and dense manner. In the Periodontitis group, collagen fibers exhibited a disordered and irregular structure, indicative of a disrupted collagenous fiber arrangement due to local inflammation. Similar to the Periodontitis group, PL/COL, PUE/COL, and Mino groups exhibited disorganized collagen deposition, suggesting that single-component hydrogels or conventional antibiotics are insufficient to restore the connective tissue structure in an inflammatory milieu. In contrast, following the treatment with PL/PUE/COL, the periodontal tissues displayed denser and more orderly collagenous fibers.

To evaluate the local inflammatory and regenerative microenvironment, immunohistochemical staining of TNF-α and TGF-β around the periodontal microenvironment was performed. As shown in Supplementary Fig. 23a, compared to the elevated TNF-α expression in the Periodontitis group, all treatments attenuated the inflammatory marker to varying degrees. While the PL/COL and the Mino groups provided partial reductions, both PUE-containing hydrogels (PUE/COL and PL/PUE/COL) more effectively downregulated TNF-α to levels comparable to the Blank group (Supplementary Fig. 23b). We further investigated the local expression levels of TGF-β, a pivotal mediator of tissue repair, to determine the underlying regenerative capacity of the periodontal microenvironment (Supplementary Fig. 24a). Compared to Blank group, TGF-β levels were significantly reduced in the Periodontitis group. While PL/COL, PUE/COL, and Mino groups provided only a moderate recovery of TGF-β, the PL/PUE/COL treatment effectively restored its expression, showing no significant difference from the Blank group (Supplementary Fig. 24b). These findings indicate that while PUE attenuates inflammation, the synergistic effect of the supramolecular system is essential to re-establish a microenvironment favorable for tissue regeneration. Moreover, following PL/PUE/COL treatment, no notable signs of inflammation or lesions were observed in main organ tissues (Supplementary Fig. 25), indicating the nontoxic nature of the supramolecular hydrogel toward normal tissues and organs.

## Discussion

In summary, we successfully developed an injectable PL/PUE/COL supramolecular hydrogel to address the therapeutic challenge of intracellular bacteria-induced immune-metabolic dysfunction in periodontitis. The supramolecular amorphization of PUE not only effectively improves its low solubility and permeability, but also enables efficient intracellular accumulation via supramolecular complex-mediated endocytosis.

The PL/PUE/COL supramolecular hydrogel achieves effective pathogen clearance through a dual-compartment strategy. Extracellularly, it eliminates pathogens through a synergistic mechanism where PL damages the bacterial outer membrane and PUE disrupts the inner membrane, reducing the bacterial burden and impairing pathogen invasiveness. Intracellularly, by protecting the lysosomal membrane from bacteria-induced oxidative damage, it restores the lysosomal homeostasis required for effective bactericidal function. The restoration of lysosomal activity not only helps eradicate intracellular bacteria, but also reverses the bacteria-mediated immune-metabolic dysregulation. Such an integrated system bridges pathogen clearance with osteo-immune remodeling, providing a promising strategy to disrupt the vicious cycle of chronic inflammation and bacterial persistence in periodontitis.

While the supramolecular co-assembly effectively overcomes PUE’s inherent constraints of solubility and permeability, certain limitations remain. The specific regulatory pathways of lysosomal homeostasis need to be further elucidated. The precise intracellular trafficking kinetics and the specific interactions of these complexes at the subcellular structural level also warrant deeper mechanistic investigation. Drawing inspiration from advanced platforms that leverage precise molecular architectures for multi-level biological activities, future studies could integrate our easily fabricated and low-cost supramolecular system with advanced nanotechnology to construct nanomedicines with precisely controlled strategies^73–76^. Building upon this co-assembly mechanism, targeted optimization and functional modification of the constituent components will be essential to achieve cell- and organelle-specific targeting, thereby further advancing the clinical translation of this system.

## Methods

### Construction and characterization of PL/PUE/COL supramolecular system

#### Construction of a supramolecular system

The PL/PUE/COL supramolecular system was constructed via a facile solution co-assembly strategy. Briefly, PL (2 mg/mL, Aladdin, China) and recombinant human type I collagen (COL, 100 mg/mL, Chuangjian, China) were first dissolved in phosphate-buffered saline (PBS; Gibco, USA) and thoroughly mixed. Subsequently, PUE (2, 4, 6, 8, and 10 mg/mL, Aladdin, China) was introduced into the mixture. The resultant solution was subjected to continuous magnetic stirring at room temperature for 1 h, to ensure homogeneity and induce the thermodynamic equilibration of the supramolecular precursors prior to gelation.

#### Physicochemical characterization

To evaluate the solubilization effect of the PL/PUE/COL system, PUE (0, 2, 4, 6, 8, and 10 mg/mL, denoted as PUE_2_ to PUE_10_) was mixed with PBS, COL, or PL/COL solutions. The mixtures were thoroughly stirred, and photographs were taken immediately and after 5 min of standing. To quantify solution turbidity, the absorbance at 600 nm was measured using a NanoDrop spectrophotometer (Thermo Fisher, USA).

To validate the solubilization capacity and supramolecular co-assembly behavior within a physiological oral microenvironment, artificial saliva (AS; Solarbio, China) was utilized as the solvent. COL (100 mg/mL) was first dissolved in AS, followed by the addition of PUE (10 mg/mL), and the co-assembly process was conducted as described above. The control group was prepared by adding PUE (10 mg/mL) directly into pure AS. The mixtures were thoroughly stirred, and macroscopic photographs were captured immediately and after 5 minutes of standing, to evaluate the solubility and sedimentation.

The supramolecular complexes were freeze-dried for further physicochemical characterization. The crystal structures were analyzed by X-ray diffractometry (XRD; D8 ADVANCE, Germany), and the functional groups were detected by Fourier transform infrared spectrometry (FTIR; Nicolet iS5, USA). ^1^H NMR spectra were recorded using a Bruker AVANCE II spectrometer (Bruker, 400 MHz, Germany) to investigate the intermolecular interactions. The freeze-dried samples were completely dissolved in deuterated DMSO. The chemical shifts were reported in parts per million (ppm). The thermal properties of the supramolecular complexes were characterized using differential scanning calorimetry (DSC; DSC3, Switzerland). The thermal scans were performed under a constant nitrogen purge over a temperature range of 20 to 190 °C, with a heating rate of 5 °C/min.

The antioxidant capacity of the supramolecular complexes was evaluated using the DPPH radical scavenging assay. DPPH powder (Macklin, China) was added to anhydrous ethanol and diluted to 80 µg/mL by ultrasound until it was completely dissolved. Sample solutions (COL, PUE_2_/COL, PUE_4_/COL, PUE_6_/COL, PUE_8_/COL, PUE_10_/COL, PL/COL, and PL/PUE_10_/COL) were thoroughly mixed with equivalent volumes of freshly prepared DPPH-ethanol solution. The mixtures were incubated in the dark at room temperature for 60 min. The absorbance was then measured at 517 nm. The DPPH radical scavenging rate was calculated using Eq. (1).

1 $${{{\rm{DPPH\,Clearance}}}}(\%)=[1-({A}_{t}-{A}_{b})/{A}_{c}]\times 100$$

where *A*_*c*_ is the absorbance of DPPH at 517 nm when no sample was added; *A*_*t*_ is the absorbance at 517 nm after mixing the sample with DPPH for 60 minutes; *A*_*b*_ is the absorbance of the sample without the addition of DPPH at 517 nm.

The transmucosal permeability of PUE under different conditions was evaluated using freshly excised porcine mandibular gingiva as an ex vivo model. To visualize the penetration behavior, PUE was labeled with Cy3. The experimental groups included: PUE suspension in PBS, PUE solution in 10% DMSO, PUE/COL, and PL/PUE/COL. The PUE concentration in these groups was 10 mg/mL. The varying formulations were applied to the mucosal surface of the gingival tissues. After incubating at 37 °C for 1.5 h, the tissues were washed with PBS, embedded and cryosectioned. 4’,6-diamidino-2-phenylindole (DAPI; Solarbio, China) was used for nuclear staining. The cross-sectional fluorescence distribution was imaged using a fluorescence microscope (Leica, DMi8, Germany).

To investigate the intracellular internalization of PUE, RAW 264.7 were seeded in a 24-well plate at a density of 5 × 10^4^ cells/mL and cultured for 12 hours to allow for adhesion. The DMEM culture medium was then replaced with fresh DMEM containing one of the following treatments: PUE/PBS, PUE/DMSO (0.1% DMSO concentration), PUE/COL, or PL/PUE/COL. The equivalent concentration of PUE was maintained at 20 μg/mL across all groups. Following a 1.5 h incubation at 37 °C, the cells were washed three times with cold PBS to remove non-internalized compounds. The cell membranes were labeled with DiO membrane dye (Beyotime, China) according to the manufacturer’s protocol. The fluorescence signals were subsequently captured using fluorescence microscopy (Leica, DMi8, Germany).

#### Molecular dynamics simulation

The structure of the PUE molecule was optimized at B3LYP-D3(BJ)/6-31G(d,p) level using Gaussian 16 A.03 software, and vibration analyses were carried out to ensure that the structures have no imaginary frequency^77,78^. The RESP charge was calculated using Multiwfn 3.8(dev) software, and the GAFF2 force field parameters were obtained using the acpype code^79^. The initial coordinates of the COL model were derived from the Protein Data Bank (PDB ID: 1K6F). A 15-mer PL was constructed with fully protonated side chains (-NH^3+^) to reflect its physiological cationic state. Both the COL and PL models were terminally capped at their N- and C-termini to neutralize unnatural terminal charges. The Amber ff14SB force field parameters were used for the protein, and the TIP3P water model was used here. Molecular dynamics simulations were performed using GROMACS 2021.7 software. Long-range electrostatic interactions were treated with the particle mesh Ewald (PME) method with 1.2 nm as the Coulomb cutoff, and the van der Waals (vdW) interactions were treated with the force-switching method, where the forces smoothly decayed to zero between 0.9 and 1.2 nm to reduce the cutoff noise^80^. The LINCS algorithm was used to apply the bond constraint related to hydrogen. All the simulation systems were energy-minimized for 5000 steps using the steepest descent algorithm, and then underwent 1 ns of NPT pre-equilibration run using leap-frog MD integrator and followed by a 300 ns unrestrained production run with a time step of 1 fs. During all the simulations, temperature was maintained at 298.15 K using the velocity rescaling (V-rescale) thermostat (with τ_t_ = 1.0 ps), and pressure was kept at 1 bar by C-rescale isotropic barostat (with τ_p_ = 2.0 ps)^81^. The three-dimensional periodic boundary condition (PBC) was used. Results were visualized using VMD 1.9.3 software.

### Fabrication and characterization of PL/PUE/COL supramolecular hydrogel

#### Fabrication of supramolecular hydrogel

The PL/PUE/COL supramolecular hydrogels were fabricated by enzymatic crosslinking. MTG was added to the equilibrated supramolecular system at a concentration of 40 U/g protein. The solution was gently mixed to avoid bubble formation and immediately transferred into Teflon molds to form PL/PUE/COL hydrogels at 37 °C for 10 min. COL, PL/COL, and PUE/COL hydrogels were synthesized using a similar method with or without the addition of PL or PUE.

#### Physicochemical characterization

The injectability evaluation of PL/PUE/COL was performed utilizing a 1 mL syringe with a 26G needle. Immediately after adding MTG, the hydrogels were loaded into the syringe and injected at a constant rate. The adhesive performance of PL/PUE/COL hydrogel was observed and recorded by photograph. To study the viscoelastic properties, dynamic oscillatory amplitude sweeps were performed using an Anton Paar MCR 302 rheometer (Austria) with a parallel plate geometry, at a constant normal force of 1 N and a frequency of 10 rad/s. All measurements were performed at 25 °C. Storage modulus (G’) and loss modulus (G’’) were calculated. Furthermore, the mechanical properties were studied by a universal testing machine (INSTRON, USA) using a compression sensor. The measurement distance was 4 mm, and the measuring speed was 1 mm/min. The stress-strain curve and compressive strength were obtained, while the compression modulus was calculated from the slope from 10 to 20% strain in the stress-strain curve. The micromorphology of the supramolecular hydrogel was characterized using scanning electron microscopy (SEM; JEOL Ltd., Japan). Crystal structures and functional groups were detected by XRD and FTIR, respectively. The release of total PUE content was detected using a NanoDrop spectrophotometer (Thermo Fisher, USA) according to a previous study^82^. In brief, PUE standard solutions ranging from 0.01 to 0.5 mg/mL were prepared using PBS, and the absorbance at 250 nm was measured to establish the standard curve. The hydrogel was placed in 5 mL of PBS at 37 °C. At specific time points within 21 days, the solution absorbance was measured at 250 nm, and corresponding concentrations were calculated using the standard curve.

### In vitro antibacterial property

To evaluate the antibacterial activity, *P. gingivalis* (ATCC 33277) or *F. nucleatum* (ATCC 25586) suspensions (300 μL, 1 × 10^6^ CFU/mL) were incubated with 100 μL of sterilized hydrogels in a 48-well plate under anaerobic conditions (10% H_2_, 10% CO_2_, 80% N_2_, 37 °C). The following seven groups were used: blank control group (untreated bacteria), labeled as “Blank”; COL group (pure collagen hydrogel), labeled as “COL”; collagen loaded with 2 mg/mL PL, marked as “PL/COL”; collagen loaded with 4 mg/mL PL, marked as “PL_4_/COL”; collagen loaded with 10 mg/mL PUE, labeled as “PUE/COL”; collagen loaded with 2 mg/mL PL and 10 mg/mL PUE, labeled as “PL/PUE/COL”; treatment of 20 μg/mL minocycline hydrochloride (Apex Bio, USA), labeled as “Mino”.

The OD values of the bacterial suspensions were measured at 600 nm every 24 h. After 72 h of incubation in an anaerobic environment, the obtained bacterial suspensions were diluted, before 100 μL were taken to spread on brain heart infusion (BHI) blood agar plates. After culturing in an anaerobic environment at 37 °C for 96 h, the bacterial colonies on the plates were photographed, and the antibacterial efficiency was calculated for quantitative analysis using Eq. (2).

2 $${{{\rm{Antibacterial\,ratio}}}}(\%)=({N}_{0}-{N}_{1})/{N}_{0}\times 100\%$$

Here, *N*_*0*_ represented the number of bacteria without any treatment, while *N*_*1*_ represented the number of bacteria cultured with different hydrogels.

For the bacterial Live/Dead assay, samples were collected and treated with the Live/Dead bacterial staining kit (Apex Bio, USA), according to the manufacturer’s instructions. The staining operation proceeded under dark conditions. Live bacteria showed green fluorescence, and dead bacteria showed red fluorescence. The stained bacteria were observed using a fluorescence microscope (Leica, DMi8, Germany). To observe the morphology of the bacteria cocultured with hydrogels, the bacterial precipitation was treated with 2.5% Glutaraldehyde EM Grade (Solarbio, China) at 4 °C overnight. Then, the samples were dehydrated in a gradient ethanol solution of 30, 50, 75, 85, 95, and 100% sequentially. Morphology observation was under SEM (JEOL Ltd., Japan) with the exposure parameters of 20 kV.

A dual-species infection model was established. *P. gingivalis* (ATCC 33277) and *F. nucleatum* (ATCC 25586) suspensions (1 × 10^6^ CFU/mL) were mixed at a 1:1 volume ratio to acquire a dual-species bacterial suspension^83^. Subsequently, 300 µL of the suspension was further incubated with 100 µL of sterilized hydrogels in a 48-well plate at 37 °C under anaerobic conditions. The OD values at 600 nm were monitored every 24 h over a 72-h period.

To evaluate the permeability of bacterial cell membranes, the leakage of proteins was detected in bacteria. The protein content in the supernatant was determined using the bicinchoninic acid (BCA) reagent kit (Solarbio, China) by measuring the absorbance at 562 nm. The membrane potential of bacteria was determined using DiBAC4(3), a voltage-sensitive fluorescent dye. When the membrane voltage increased, the DiBAC4(3) dye molecules were enriched on the membrane surface. After incubating *P. gingivalis* with different hydrogels in an anaerobic environment at 37 °C for 12 h, the collected bacterial suspensions were washed with PBS and introduced with DiBAC4(3) (5 μM), incubating at 37 °C for 30 min. Subsequently, fluorescence images were captured using a fluorescence microscope (Leica, DMi8, Germany) to assess the bacterial potential. The fluorescence intensity was represented as a 3D surface plot, and the mean fluorescence intensity was quantified using Fiji ImageJ software.

### Biocompatibility

Mouse macrophage RAW 264.7 cell line were obtained from the ATCC (Manassas, VA, USA). RAW 264.7 cells were cultured in Dulbecco’s modified Eagle’s medium (DMEM; Gibco, USA) supplemented with 10% fetal bovine serum (FBS; Vazyme, China). Cells were cultured under a 5% CO_2_ atmosphere in an incubator at 37 °C, and the medium was changed every 2 days.

CCK-8 assay was conducted to preliminarily evaluate the potential cytotoxicity of hydrogels. 0.5 g of sterilized hydrogels (COL, PL/COL, PL_4_/COL, PUE/COL, PL/PUE/COL) were immersed in 5 mL of DMEM medium at 37 °C for 24 h to prepare the hydrogel extracts^50^. The extracts were sterile-filtered (0.22 μm, Millipore), supplemented with 10% FBS, using for CCK-8 assay. RAW 264.7 cells (1 × 10^4^ per well) were seeded in 96-well plates. Cells were cultured with hydrogel extracts for 1, 3, and 5 days. According to the manufacturer’s instructions, at each designated time point, the existing supernatant was replaced with a working solution of DMEM containing CCK-8 (Apex Bio, USA). The plates were then incubated in the dark at 37 °C for 30 min, after which the absorbance at 450 nm was recorded, and the proliferation percentages were calculated using Eq. (3).

3 $${{{\rm{Cell\,proliferation}}}}\,(\%)=[({{OD}}_{{Experiment}}-{{OD}}_{{Blank}})/({{OD}}_{{Ctrl}}-{{OD}}_{{Blank}})]\times 100$$

To further assess cellular viability, 100 μL of each sterilized hydrogel was fabricated in a 48-well plate. Subsequently, RAW 264.7 macrophages were seeded directly onto the hydrogel surfaces at a density of 5 × 10^4^ cells per well. Following a 24-h incubation, the cells were stained using a Calcein-AM/PI Double Stain Kit (Solarbio, China) under dark conditions according to the manufacturer’s protocol. Fluorescence imaging was acquired utilizing a fluorescence microscope (Leica, DMi8, Germany).

Hemolysis assay was performed to evaluate the blood compatibility of hydrogels. About 0.02 g of the hydrogels (COL, PL/COL, PL_4_/COL, PUE/COL, and PL/PUE/COL) were incubated in 1 mL 0.9% normal saline at 37 °C for 30 min, while pure 0.9% normal saline and distilled water was set as negative control and positive control, respectively. Then, 20 μL of defibrillated rabbit blood (Solarbio, China) was added and incubated at 37 °C for 1 h. Afterward, the mixture was centrifuged at 200×*g* for 5 min, and the absorbance of the supernatant was measured. The hemolysis ratio (HR) was calculated using Eq. (4).

4 $${{{\rm{HR}}}}(\%)=[({{OD}}_{{sample}}-{{OD}}_{{negative}})/({{OD}}_{{positive}}-{{OD}}_{{negative}})]\times 100$$

To evaluate cellular adhesion and morphology, RAW 264.7 cells were plated directly onto the hydrogels in 48-well plates at a density of 3 × 10^4^ cells per well. Following a 48-h incubation, the samples were gently rinsed using PBS (Gibco, USA) and fixed in 2.5% Glutaraldehyde EM Grade (Solarbio, China) at 4 °C overnight. Subsequently, the samples underwent sequential dehydration through ascending concentrations of ethanol (30, 50, 75, 85, 95, and 100%) prior to gold sputtering. Scanning electron microscopy (SEM; JEOL Ltd., Japan) was performed at an accelerating voltage of 20 kV to capture the morphological details.

### Anti-inflammatory effect evaluation

An inflammatory model was established using LPS from E. *coli*. 100 μL of PL/COL (loading with 2 mg/mL PL), PUE/COL (loading with 10 mg/mL PUE), PL/PUE/COL (loading with 2 mg/mL PL and 10 mg/mL PUE) hydrogel was sterilized and fabricated in a 48-well plate. Then, RAW 264.7 macrophages (5 × 10^4^ cells/mL) were seeded on hydrogels in a 48-well plate. After incubation for 24 h, all groups were treated with 100 ng/mL LPS for another 24 h. Blank group was set as a negative control with no LPS stimulation, while the LPS group was set as a positive group with LPS stimulation.

For ROS-scavenging detection, the DCFH-DA (Solarbio, China) probe was used to detect cellular ROS levels. DCFH-DA was diluted in serum-free culture medium at a 1:1000 ratio to achieve a final concentration of 10 μM, and incubated with cells at 37 °C for 30 min. After gently washing with serum-free culture medium three times, cells were observed under a fluorescence microscope (Leica, DMi8, Germany). For quantitative analysis, three regions in each group were randomly selected, and quantified using ImageJ software.

Macrophage polarization was investigated by Immunofluorescent staining. RAW 264.7 cells (3 ×  10^4^/well) were seeded on hydrogels (200 μL/well) in a 24-well plate. As described above, cells were stimulated with LPS for 24 h. Cells were cleaned with PBS and treated with 4% paraformaldehyde for 20 min. After being treated with 0.25% Triton for 8 min and 1% bovine serum albumin (BSA) solution for 30 min, cells were incubated with anti-iNOS primary antibody (1:100 dilution, ER1706-89, Huabio, China) and anti-CD206 primary antibody (1:400 dilution, ET1702-04, Huabio, China) at 4 °C overnight. Then Alexa Fluor® Plus 488 Goat anti-Rabbit secondary antibody (1:1000 dilution, A32731, Invitrogen, USA) was incubated with cells at 37 °C for 1 h under dark conditions. DAPI (Solarbio, China) was used for nuclear staining. The immunofluorescence-stained cells were visualized under the fluorescence microscope (Leica, DMi8, Germany). The fluorescence intensity was represented as a 3D surface plot using Fiji ImageJ software. Three regions in each group were randomly selected for quantitative analysis, and the relative fluorescence intensity was quantified using ImageJ software.

For real-time quantitative PCR, RAW 264.7 cells were seeded on hydrogels (1 mL/well) in 6-well plates at a density of 1 × 10^6^ cells per well. Following 24 h of initial adherence, the cells were challenged with LPS for an additional 24 h. Total cellular RNA extraction, subsequent cDNA synthesis, and quantitative PCR amplification were executed utilizing the FastPure® Cell/Tissue Total RNA Isolation Kit V2 (Vazyme, China), HiScript® III RT SuperMix (+gDNA wiper) (Vazyme, China), and Taq Pro Universal SYBR qPCR Master Mix (Vazyme, China), respectively. The reactions were run on an ABI 7300 Real-Time PCR System (Applied Biosystems, USA) according to the manufacturer’s protocols. The 2^–ΔΔCt^ method was used to assess the expression levels of the target genes (*CD86, iNOS, IL-1β, TNF-α*, and *TGF-β*). Glyceraldehyde 3-phosphate dehydrogenase (*GAPDH*) served as the endogenous housekeeping gene. The primer sequences are documented in Supplementary Table 1.

### Phagocytic activity and intracellular bacteria elimination effect

Polystyrene microspheres with green fluorescence (size 100 nm) were prepared for the macrophage phagocytic activity assay. The fluorescent microspheres were preconditioned by incubation in DMEM containing 1% BSA, which was performed at 37 °C for 30 min, protected from light. The microspheres were sonicated for 5 min before use. RAW 264.7 cells (3 × 10^4^/well) were seeded on a 24-well plate. After incubation for 24 h, cells were cocultured without or with hydrogels (PL/COL, PUE/COL, and PL/PUE/COL) for another 24 h using a transwell chamber. Then, the pretreated microspheres were added at a density of 1 × 10^7^/well, and cultured with cells at 37 °C for 3 h. After gentle washes with PBS three times, the cell membranes were labeled with Dil dye (Beyotime, China) according to the manufacturer’s protocol. The cells were subsequently fixed with 4% paraformaldehyde for 20 min, and the nuclei were stained with DAPI. Cells were observed under a fluorescence microscope (Leica, DMi8, Germany).

To label with FITC, *P. gingivalis* were centrifuged (6000×*g*, 10 min, 4 °C), washed with PBS three times, and resuspended in 0.5 M NaHCO₃ (2.5 × 10⁹ CFU/mL). FITC (0.15 mg/mL) was added to the bacterial suspension, incubating on a rotator for 30 min under dark conditions. Following incubation, the labeled bacteria were washed three times with PBS and resuspended in DMEM. RAW 264.7 cells (3 × 10^4^/well) were seeded on a 24-well plate and incubated for 24 h. Cells were infected with FITC-labeled *P. gingivalis* at a multiplicity of infection (MOI) of 100 for 3 h. After triple PBS washing, extracellular bacteria were eliminated via 1 h incubation with gentamicin and metronidazole. After washing three times with PBS, cells were then cocultured with hydrogels (COL, PL/COL, PUE/COL, and PL/PUE/COL) or 20 μg/mL minocycline hydrochloride for 3 h using a transwell chamber. Bacteria cultured without a hydrogel served as the untreated *P. gingivalis* control group (denoted as *P.g* group). Samples were collected before and after hydrogel coculture, and the cell membrane and nuclei were stained as described above. The elimination of intracellular bacteria was observed under a fluorescence microscope (Leica, DMi8, Germany).

To detect the invasion ability of pretreated bacteria, *P. gingivalis* of 1 × 10^9^ CFU/mL were pretreated by coculturing with the hydrogels (COL, PL/COL, PUE/COL and PL/PUE/COL) or 20 μg/mL minocycline hydrochloride for 12 h. Bacteria cultured without hydrogel were served as the control group, denoted as “*P.g*”. The bacteria from each group were harvested by centrifugation, washed, and then labeled with FITC as described above. RAW 264.7 cells (3 × 10^4^/well) were seeded on a 24-well plate and incubated for 24 h. Then, the pretreated and FITC-labeled *P. gingivalis* were added (MOI = 100) and cocultured for 3 h. After infection, cells were washed three times with PBS, followed by a 1-h incubation with medium containing metronidazole and gentamicin to eliminate extracellular bacteria. After the staining of the cell membrane (Dil) and nuclei (DAPI), the bacterial invasion capability was observed under a fluorescence microscope (Leica, DMi8, Germany). For ATP detection, the bacterial suspensions after different treatments for 12 h were centrifuged and washed three times with PBS. The ATP content in bacteria was detected with ATP Chemiluminescence Assay Kit (Elabscience, China) according to the instructions.

### Infected macrophage induced osteogenic activity

RAW 264.7 cells (1 × 10^6^/well) were seeded in six-well plate, and cultured for 24 h followed by *P. gingivalis* infection (MOI = 100) for 3 h. After clearance of extracellular bacteria, infected macrophages were then cocultured without or with hydrogels (PL/COL, PUE/COL, and PL/PUE/COL) for 12 hours. The supernatant from different groups was collected, filtered through a 0.22-μm filter and mixed with culture medium at a 1:1 ratio as the conditioned medium. About 10 mM β-glycerophosphate, 50 μg/mL L-ascorbic acid, and 10% FBS were added to the conditioned medium to formulate the osteogenic induction medium.

Mouse MC3T3-E1 pre-osteoblastic cells were obtained from the ATCC (Manassas, VA, USA), and were cultured in α-MEM medium supplemented with 10% FBS (Vazyme, China). MC3T3-E1 cells were seeded into a 24-well plate at a density of 5 × 10^4^/well. After culturing for 24 h, the culture medium was changed to osteogenic induction medium from different groups. The culture medium was changed every other day. After culturing for 7 days, cells were washed with PBS and then fixed in 4% paraformaldehyde for 30 min for ALP staining. ALP Staining Kit (Beyotime, China) was used according to the manufacturer’s instructions. After staining for 1 h, the staining result was observed by a stereo microscope (Olympus, SZX16, Japan). Similarly, MC3T3-E1 cells cultured in osteogenic induction medium from different groups for 14 days were fixed for ARS staining. ARS solution (Solarbio, China) was used for staining for 30 min at 37 °C, and the staining result was observed by a stereo microscope (Olympus, SZX16, Japan).

For immunofluorescence evaluations, MC3T3-E1 cells (3 × 10^3^/well) were seeded in a 24-well plate. After 24 h, the standard media was substituted with the respective conditioned media. Following a 7-day culture period, the specimens were rinsed with PBS and fixed utilizing 4% paraformaldehyde for 20 min. The fixed cells were then incubated overnight at 4 °C with an anti-ALP primary antibody (1:100 dilution, ET1601-21, Huabio, China). Subsequently, Alexa Fluor® Plus 488 Goat anti-Rabbit secondary antibody (1:1000 dilution, A32731, Invitrogen, USA) was applied at 37 °C, followed by nuclear counterstaining with DAPI (Solarbio, China). The stained cells were observed by a fluorescence microscope (Leica, DMi8, Germany). The generation of 3D surface plots and fluorescence quantification adhered to the same methods mentioned above.

To evaluate the direct osteogenic potential, hydrogel extracts were prepared by incubating the sterilized hydrogels in culture medium at 37 °C for 24 h, as described above. MC3T3-E1 cells were seeded and cultured for 24 h, before the culture medium was replaced with osteogenic induction medium supplemented with the respective hydrogel extracts. After culturing for 7 days, the cells were subjected to ALP staining and immunofluorescence staining for ALP, following the protocols described above.

### RNA-sequencing analysis

RAW 264.7 cells were infected with *P. gingivalis* as described above. After clearance of extracellular bacteria, infected macrophages were then cocultured without or with PL/PUE/COL for 12 h. Untreated cells served as the negative control. Total RNA was extracted utilizing RNAiso Plus (Takara, Japan). Then RNA quality was determined by the 5300 Bioanalyser (Agilent) and quantified using the ND-2000 (NanoDrop Technologies). The RNA-seq transcriptome library was prepared following Illumina^®^ Stranded mRNA Prep, Ligation from Illumina (San Diego, CA). Majorbio (Shanghai, China) executed the transcriptome sequencing and provided subsequent analytical support.

### Measurement of cellular ROS scavenging, lysosomal function, and mitochondrial function

After *P. gingivalis* infection, RAW 264.7 cells were further cultured without or with hydrogels (PL/COL, PUE/COL, and PL/PUE/COL) for 3 h. The detection of intracellular ROS levels was performed by using the DCFH-DA (Solarbio, China) probe. Fluorescence images were then observed under a microscope and quantified using ImageJ software.

For intracellular colocalization of bacteria and lysosomes, FITC-labeled *P. gingivalis* invaded RAW 264.7 as described above. After coculturing without or with hydrogels (PL/COL, PUE/COL, and PL/PUE/COL), cells were further stained with LysoTracker Red (Beyotime, China) for 30 min to visualize lysosomes. The stained cells were observed by a fluorescence microscope. For colocalization analysis, spatial fluorescence intensity profiles were generated using Fiji ImageJ software along the paths indicated by the white dashed arrows to map the spatial distribution of FITC and LysoTracker signals. For real-time quantitative PCR, after infected macrophages were cocultured with hydrogels, the expression levels of the target gene (*CTSD* and *LAMP1*) were assessed by the protocol described above. The primer sequences are listed in Supplementary Table 1.

The assessment of mtROS generation was conducted utilizing the MitoSOX Red mitochondrial superoxide assay kit (Beyotime, China), while the MitoTracker Green was employed to label mitochondria for colocalization. Afterward, the MMP was measured via a JC-1 assay kit (Beyotime, China) according to the manufacturer’s instructions. Red fluorescence emission from JC-1 represents an energized MMP, whereas green fluorescence indicates a lower membrane potential. The fluorescence intensity was represented as a 3D surface plot using Fiji ImageJ software. Three regions in each group were randomly selected for quantitative analysis, and the relative fluorescence intensity was quantified using ImageJ software.

### In vivo evaluation of the hydrogel function

All animal surgical procedures were approved by the Ethics Committee of West China Hospital of Stomatology, Sichuan University, with registration of WCHSIRB-D-2025−075. Six-week-old C57BL/6 mice (male, 18–20 g) were used to construct an in vivo periodontitis model. Mice were housed in a specific pathogen-free (SPF) facility at 20–22 °C and 40–60% humidity on a 12-h light/dark cycle, with ad libitum access to standard chow and water. Based on in vitro results, animals were randomly divided into healthy control group, periodontitis group, PL/COL group (periodontitis treated with PL/COL hydrogel), PUE/COL group (periodontitis treated with PUE/COL hydrogel), PL/PUE/COL group (periodontitis treated with PL/PUE/COL hydrogel) and Mino group (periodontitis treated with 20 μg/mL minocycline hydrochloride). Under a sterile environment, zoletil (80 mg/kg, Virbac, France) along with xylazine (12 mg/kg, Virbac, France) was used through intramuscular injection for anesthesia. 6-0 silk ligatures were firmly inserted sub-gingivally around the maxillary second molar^84^. After 7 days of ligation to allow for periodontitis development, sutures were removed, and 20 μL of PBS, hydrogels or minocycline hydrochloride were locally applied to the periodontal pocket around the maxillary second molar every other day through a microsyringe^84,85^. Ten days after periodontal injection, the animals were sacrificed, and the obtained maxillary bones, hearts, livers, lungs, spleens, and kidneys were collected and fixed in 4% paraformaldehyde at 4 °C for further experiments.

Fixed maxillary samples were scanned by Micro-CT (μCT-50, Scanco Medical AG, Switzerland). Scanning was performed at 200 μA and 70 kVp every 10 μm at high resolution. Micro-CT quantification of periodontal destruction was performed by measuring CEJ-ABC distances at both the mesial and distal aspects of the second molar (M2). Similarly, three-dimensional regions of interest on both sides were analyzed for BV/TV. The quantification and three-dimensional reconstruction process were conducted by Mimics Medical v20.0 (Materialise).

Maxillary samples were decalcified for 1 month. Then, the decalcified samples and main organs (heart, liver, spleen, lung, and kidney) were dehydrated with a graded series of alcohol from 75 to 100%. All samples were embedded in molten paraffin, and the embedded samples were cut into 5-μm thick sections. The maxillary samples were processed for HE staining and Masson staining, while the main organs and bone tissue were prepared for HE staining. For immunohistochemistry staining, sections of maxillary samples were processed with anti-TGF-β primary antibody (1:300 dilution, GB115739, Servicebio, China) and anti-TNF-α primary antibody (1:500 dilution, GB115702, Servicebio, China) before incubating with a secondary antibody conjugated to horseradish peroxidase (HRP). The stained tissue sections were visualized under a microscope (Leica, DMi8, Germany). Semiquantitative analysis of the average optical density was performed using ImageJ software to compare the expression levels across all groups.

### Statistics and reproducibility

Biological experiments were performed at least three times, and the results were presented as means ±  standard deviations (SD). Figures were generated using Origin 2024 and GraphPad Prism 8.0. All data were analyzed using SPSS 22.0 statistical software. One-way analysis of variance (ANOVA) with Tukey’s post hoc test was employed to analyze differences among multiple groups, and a two-tailed Student’s *t*-test was performed for the comparison between two groups. Values of *P* < 0.05 were considered statistically significant.

### Reporting summary

Further information on research design is available in the Nature Portfolio Reporting Summary linked to this article.

## Supplementary information

Supplementary Information

Reporting Summary

Transparent Peer Review file

## Source data

Source Data

## Data Availability

The main data supporting the results of this study are available within the paper and its Supplementary Information. The initial coordinates of the COL model used in this study are available in the Protein Data Bank under accession code 1K6F. Raw RNA sequencing data generated in this study have been deposited in the Genome Sequence Archive (GSA) database under accession number CRA041814. Source data are provided with this paper.
