# Supplementary material for: Supramolecular strategy for compartment pathogen clearance and immuno-metabolic homeostasis to treat periodontitis
Source: Nat Commun. 2026 Jun 18;17:7714. doi: 10.1038/s41467-026-74544-5 (PMC13434047; doi:10.1038/s41467-026-74544-5)
Supplement: Supplementary file 1 [file 41467_2026_74544_MOESM1_ESM.pdf]

# Supplementary Information for

## Supramolecular Strategy for Compartment Pathogen Clearance and Immuno-Metabolic Homeostasis to Treat Periodontitis

**This PDF file includes:**

Supplementary Figures 1 to 25

Supplementary Table 1

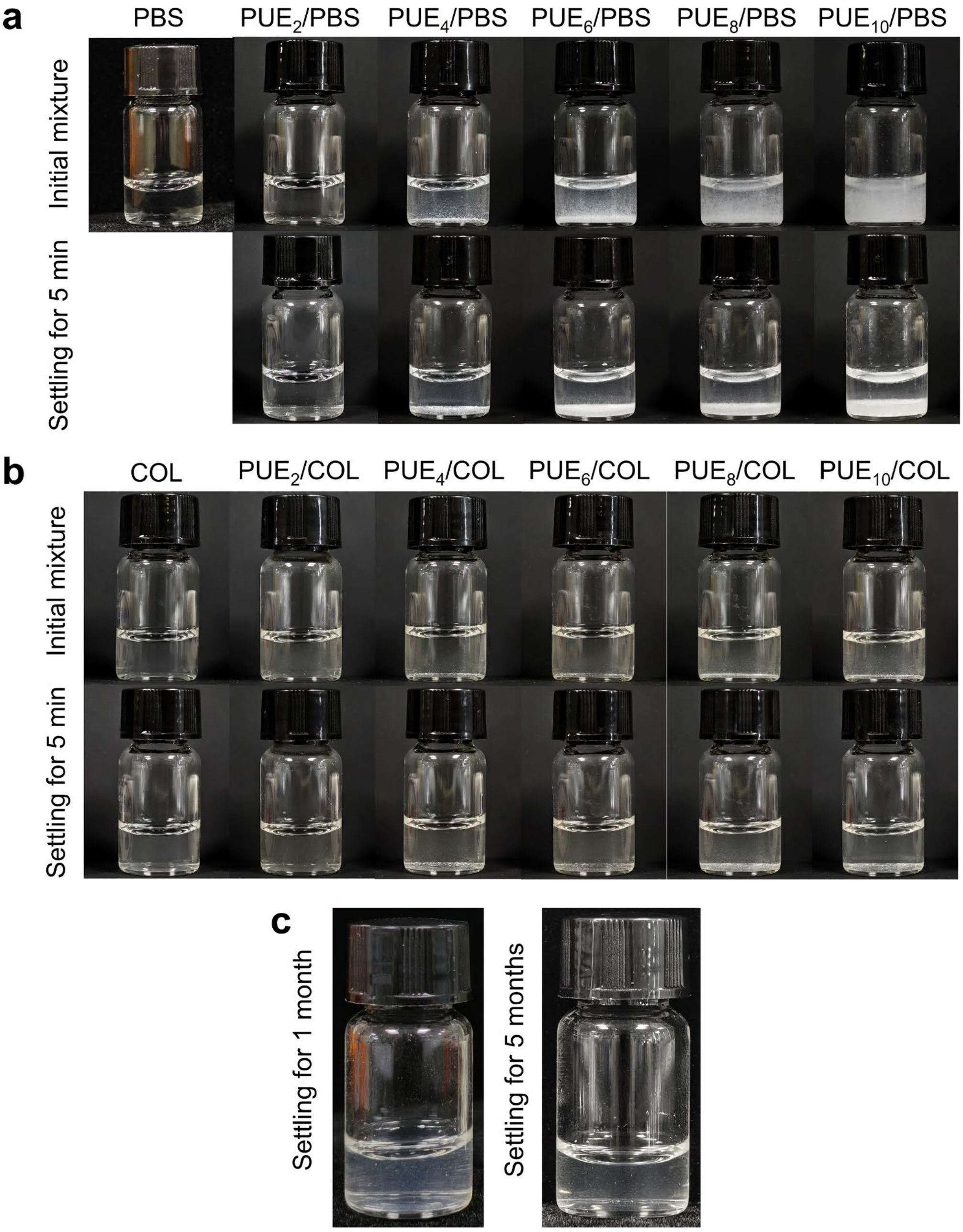


**Supplementary Fig. 1.** Optical images of the solubility and sedimentation of PUE with concentrations of 0, 2, 4, 6, 8, and 10 mg/mL (denoted as $PUE_2$ to $PUE_{10}$) in (a) PBS and (b) COL solution. (c) Image of PL/PUE/COL standing for 1 month and 5 months at 37 °C. COL recombinant human type I collagen, PL poly-ε-lysine, PUE puerarin, PBS phosphate-buffered saline.

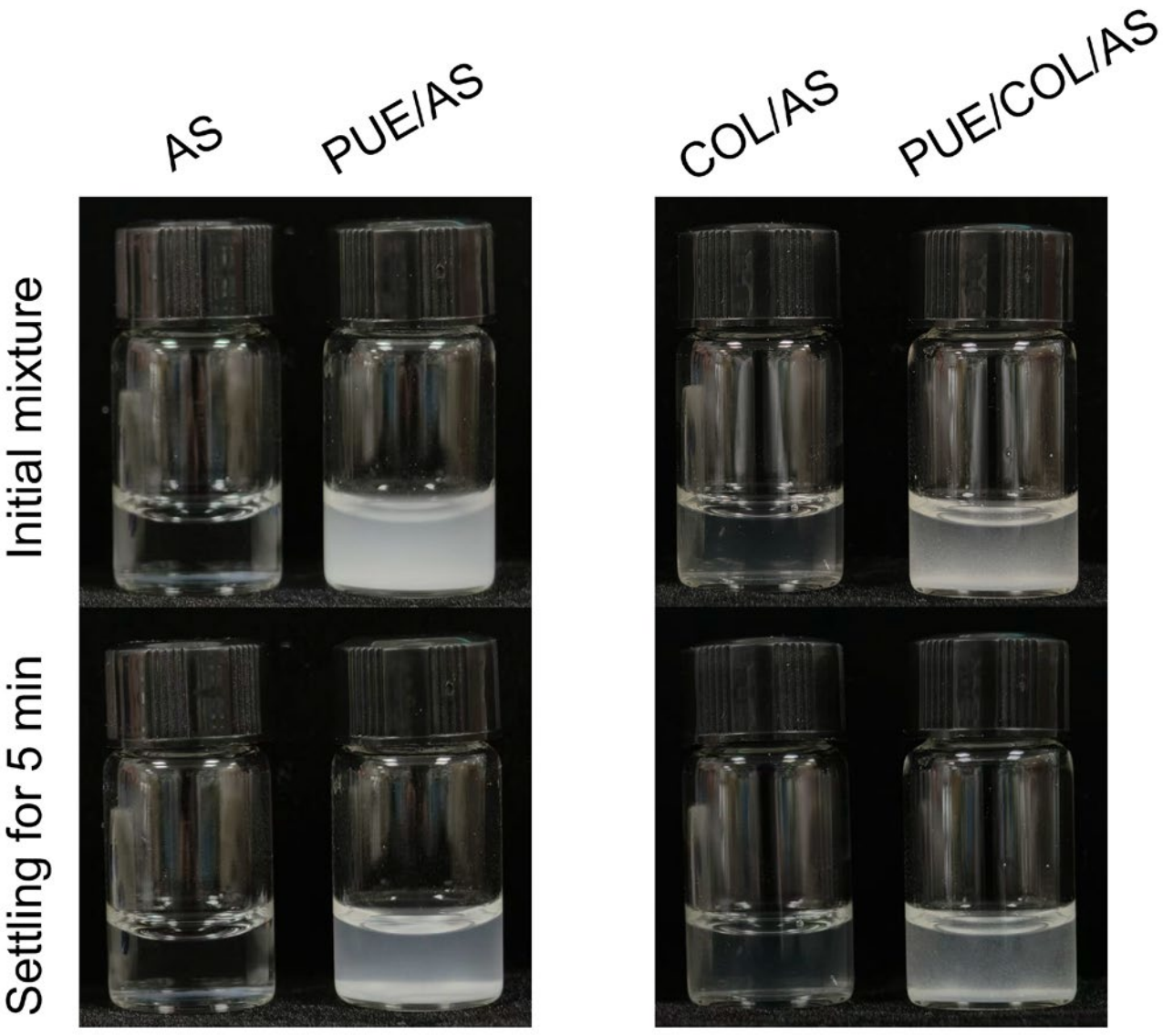


**Supplementary Fig. 2.** Optical images of the solubility and sedimentation of 10 mg/mL PUE in pure AS and COL-containing AS solution. COL recombinant human type I collagen, PUE puerarin, AS artificial saliva.

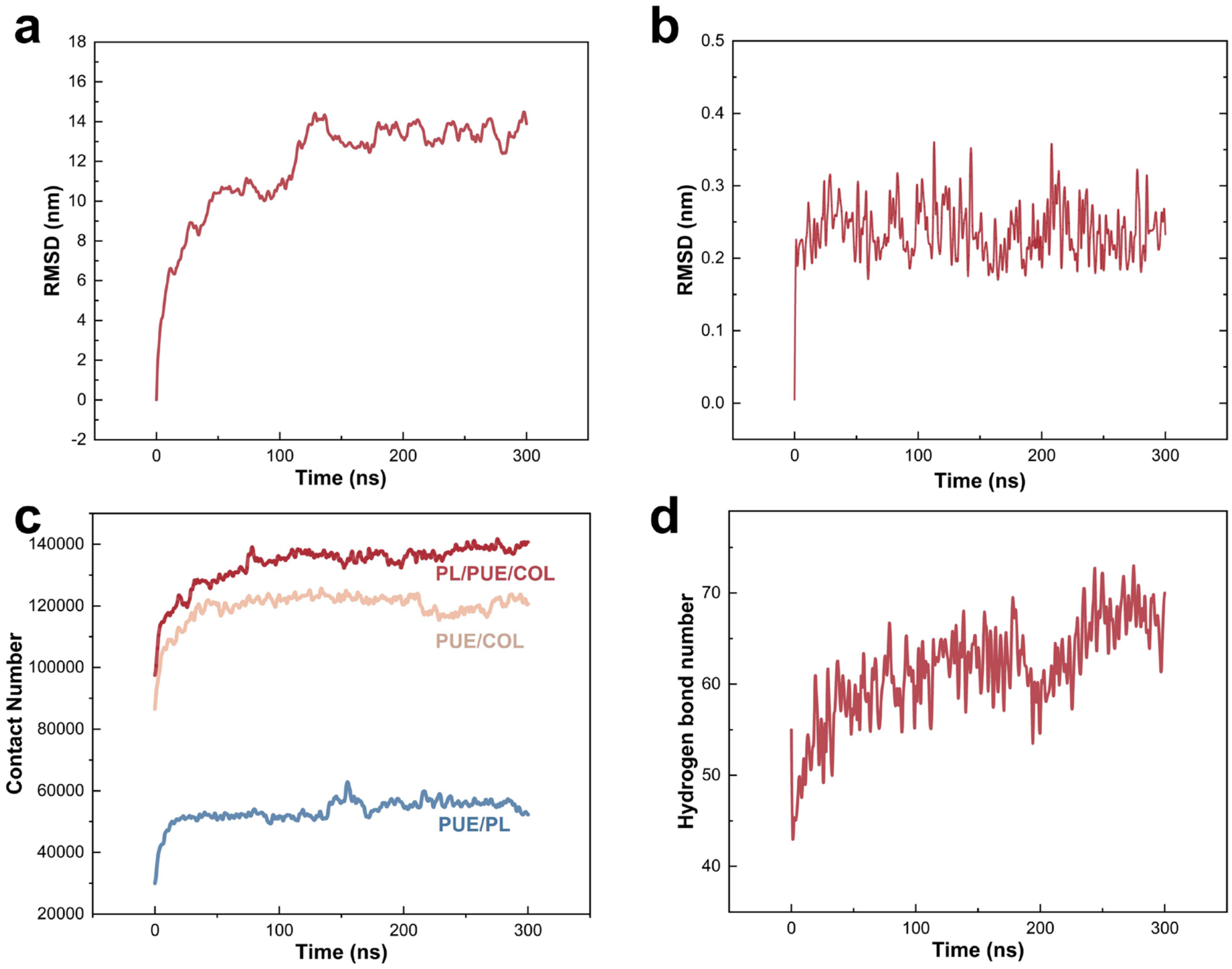


**Supplementary Fig. 3.** Structural stability and assembly dynamics of the PL/PUE/COL supramolecular complex. (a) RMSD curves of the PL/PUE/COL cluster over time. (b) RMSD curves of COL in the PL/PUE/COL system over time. (c) Contact number in the PUE/PL system, PUE/COL system and PL/PUE/COL system. (d) Number of hydrogen bonds in the PL/PUE/COL system over time. COL recombinant human type I collagen, PL poly-ε-lysine, PUE puerarin, RMSD root mean square deviation. Source data are provided as a Source Data file.

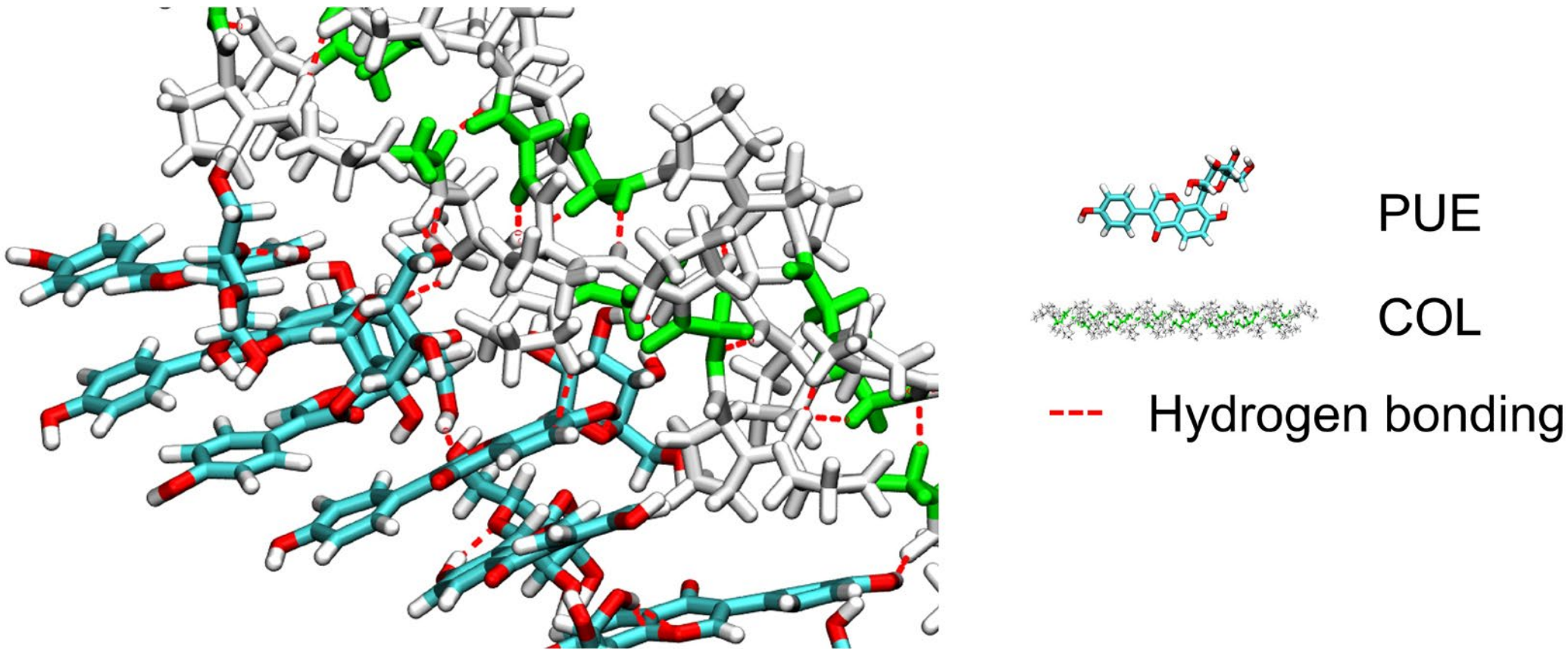


**Supplementary Fig. 4.** Intermolecular interactions between PUE and COL (based on the structural snapshot). The white areas on the COL model represent the hydrophobic regions formed by proline residues. COL recombinant human type I collagen, PUE puerarin.

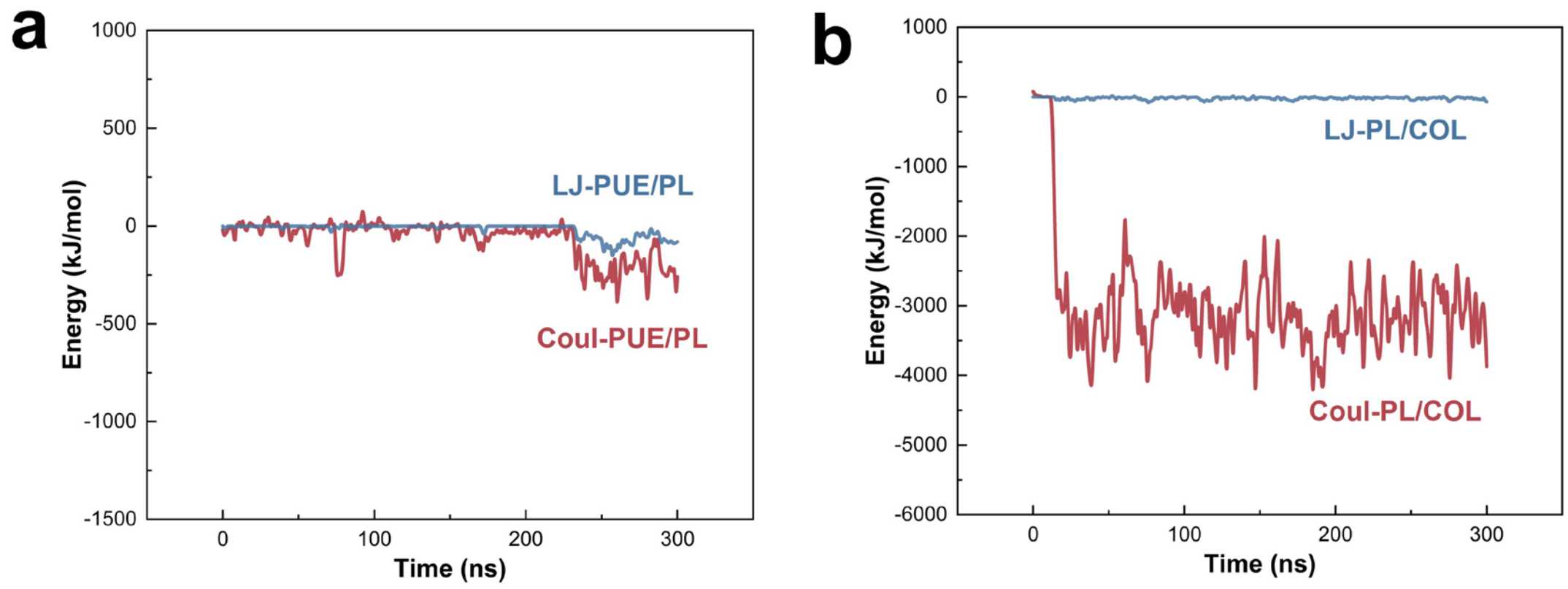


**Supplementary Fig. 5.** Interaction energy (a) between PUE and PL and (b) between PL and COL over time. COL recombinant human type I collagen, PL poly-ε-lysine, PUE puerarin, Coul Coulomb interactions, LJ Lennard-Jones potentials. Source data are provided as a Source Data file.

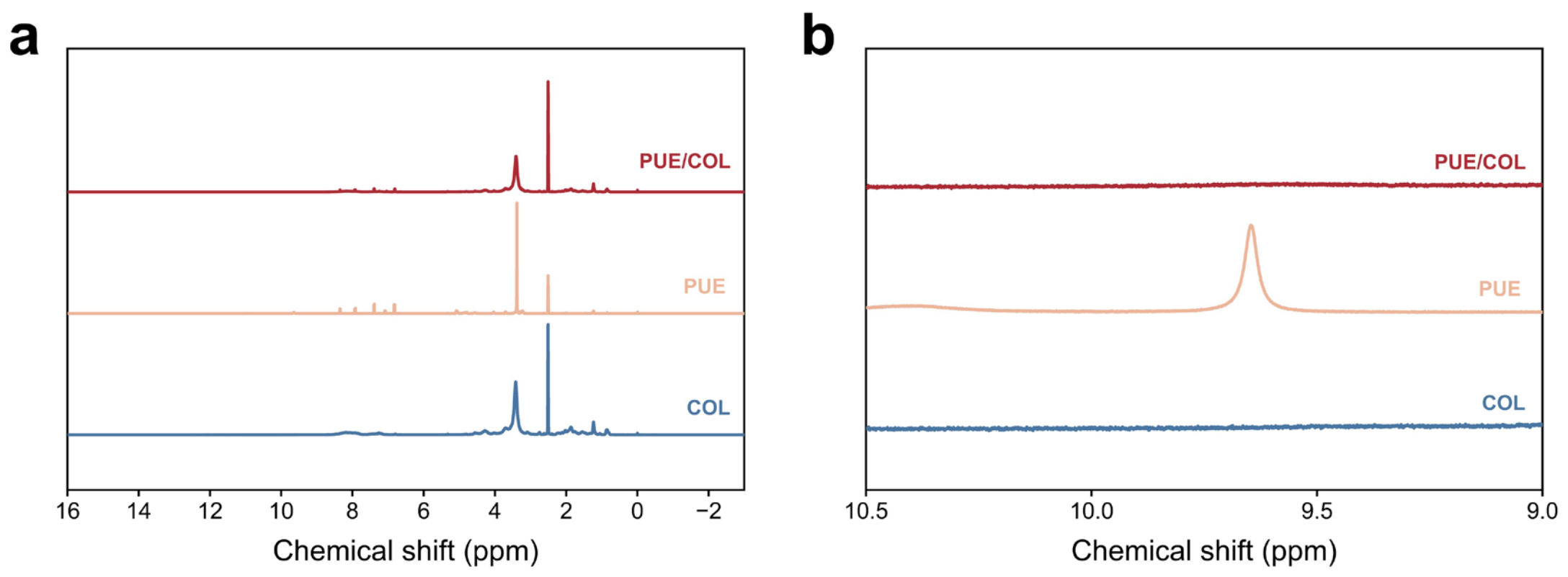


**Supplementary Fig. 6.** $^{1}$H NMR (a) full spectra and (b) corresponding locally enlarged spectra (δ 9.0-10.5 ppm) of COL, PUE, and PUE/COL. COL recombinant human type I collagen, PUE puerarin, NMR nuclear magnetic resonance. Source data are provided as a Source Data file.

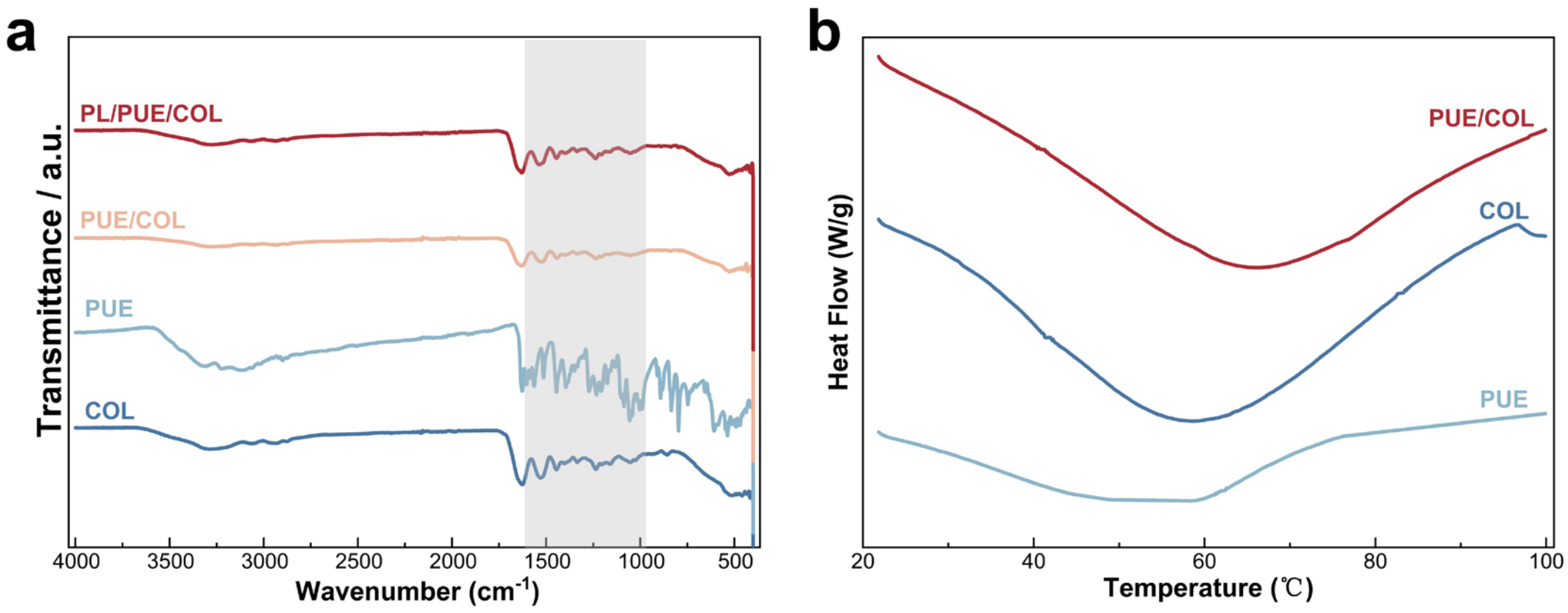


**Supplementary Fig. 7.** (a) FTIR spectra of PUE, COL, PUE/COL and PL/PUE/COL. (b) DSC thermographs of PUE, COL, and PUE/COL. COL recombinant human type I collagen, PL poly-ε-lysine, PUE puerarin, FTIR Fourier transform infrared spectrometry, DSC differential scanning calorimetry. Source data are provided as a Source Data file.

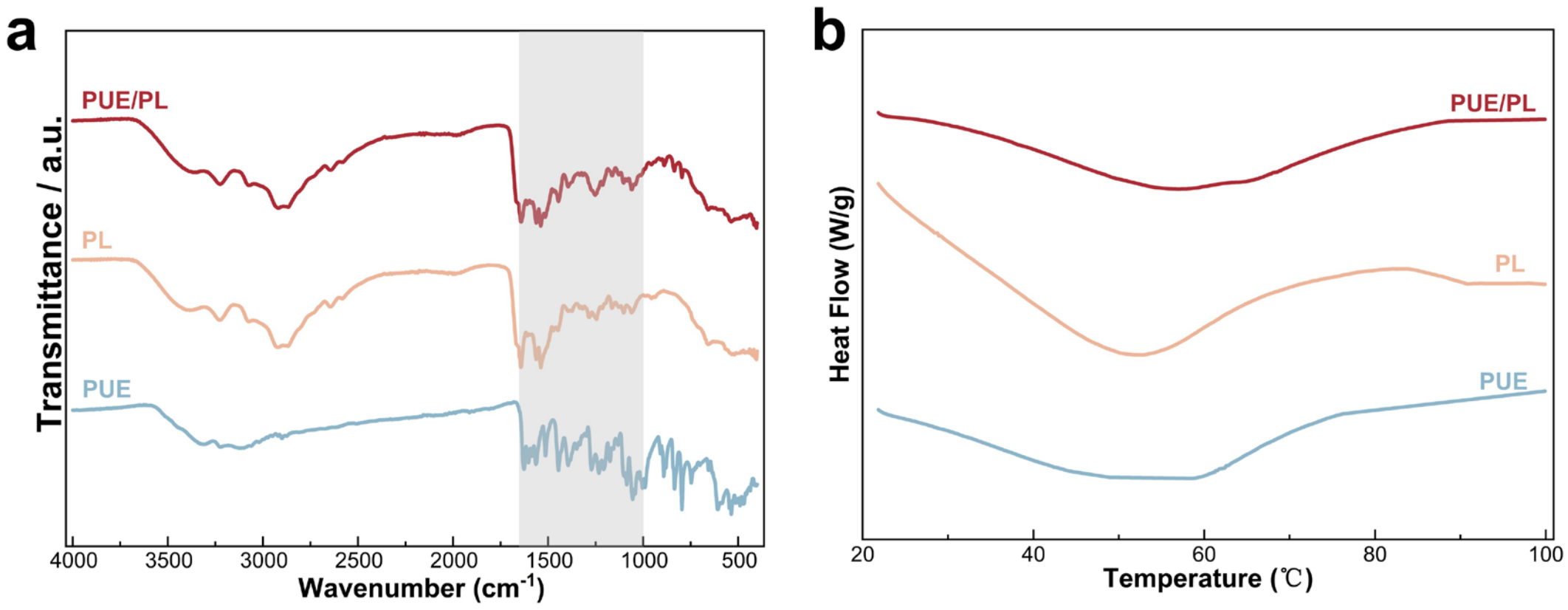


**Supplementary Fig. 8.** (a) FTIR spectra and (b) DSC thermographs of PUE, PL, and PUE/PL. PL poly-ε-lysine, PUE puerarin, FTIR Fourier transform infrared spectrometry, DSC differential scanning calorimetry. Source data are provided as a Source Data file.

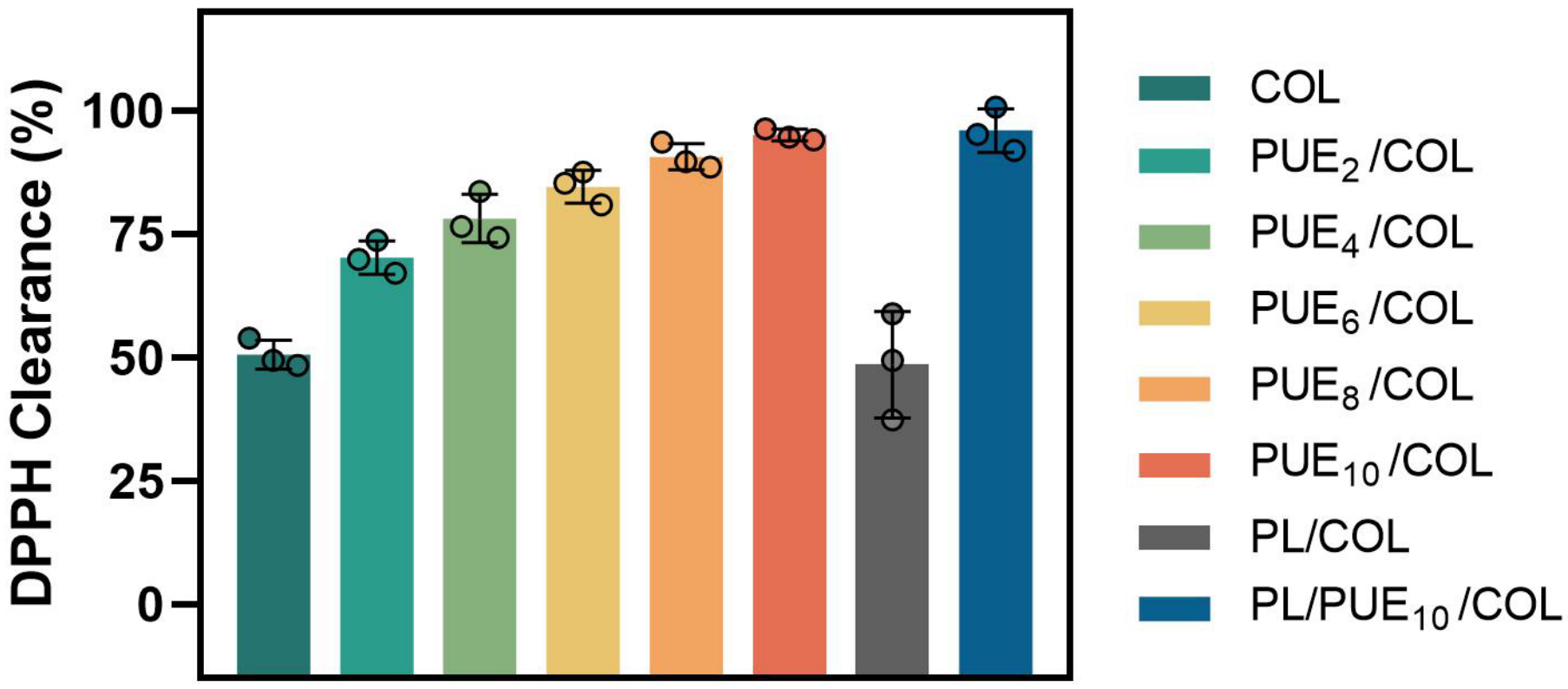


**Supplementary Fig. 9.** DPPH radical scavenging activity of COL, PUE/COL supramolecular complexes with gradient PUE concentrations (2, 4, 6, 8, 10 mg/mL, denoted as $PUE_2$/COL to $PUE_{10}$/COL), PL/COL, and PL/$PUE_{10}$/COL supramolecular complexes (*n*=3 independent experiments). All error bars represent mean ± *SD*. COL recombinant human type I collagen, PL poly-ε-lysine, PUE puerarin, DPPH 2,2-diphenyl-1-picrylhydrazyl. Source data are provided as a Source Data file.

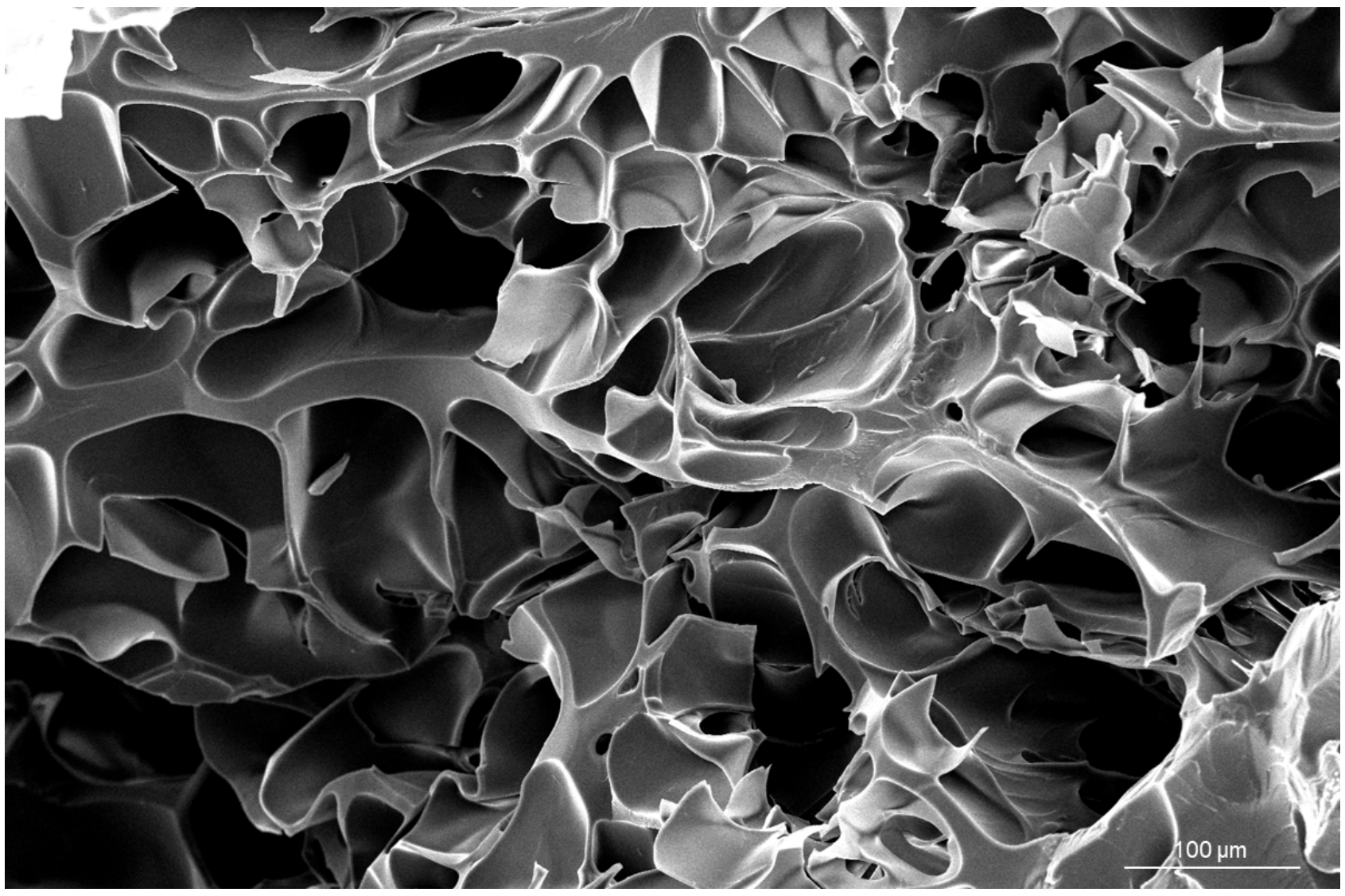


**Supplementary Fig. 10.** SEM image of freeze-dried PL/PUE/COL hydrogel. COL recombinant human type I collagen, PL poly-ε-lysine, PUE puerarin, SEM scanning electron microscopy.

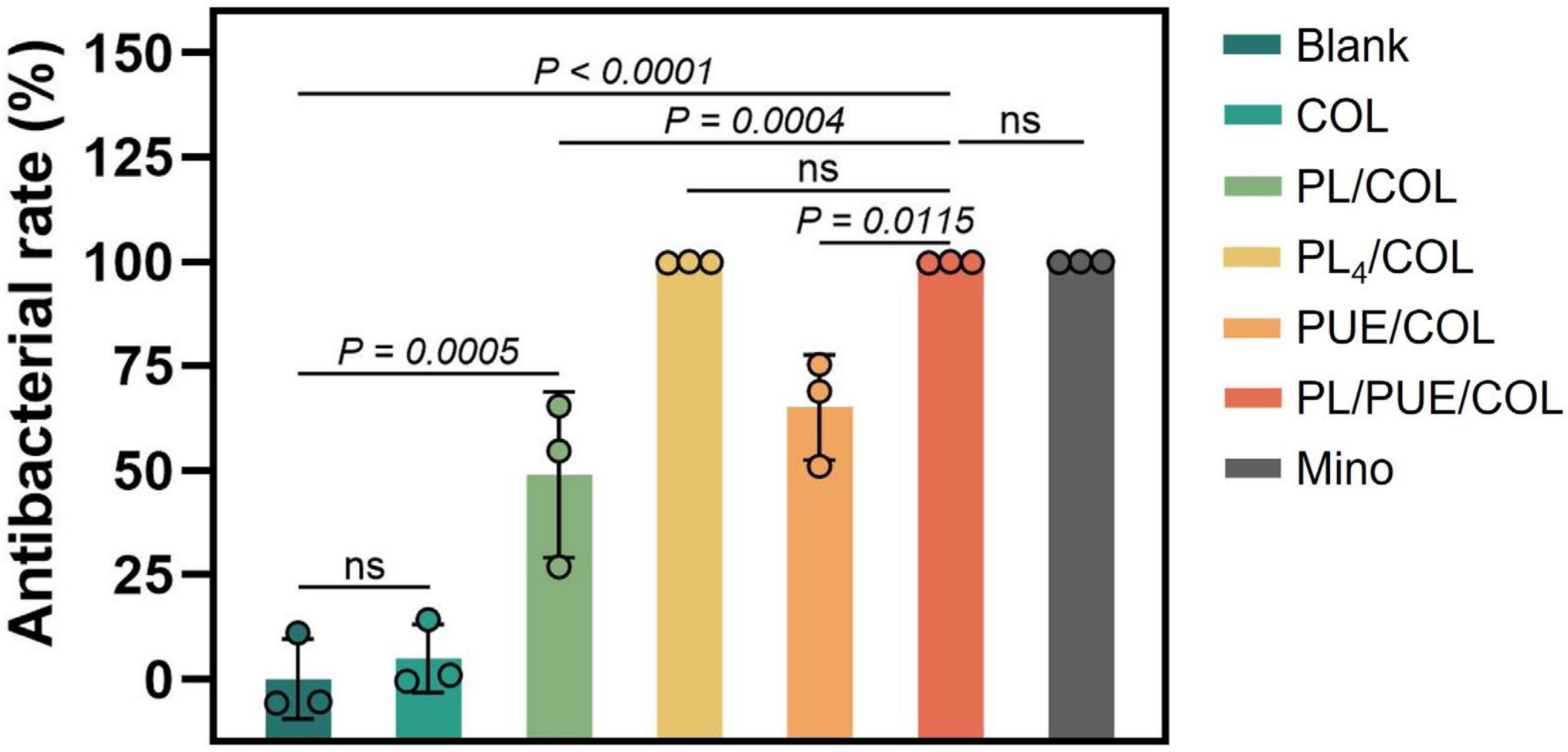


**Supplementary Fig. 11.** Quantitative analysis of corresponding antibacterial rates in Fig. 4a (*n*=3 independent biological samples). All error bars represent mean ± *SD*. *P* values were calculated by one-way ANOVA followed by Tukey's post hoc test (ns, not significant). Source data are provided as a Source Data file.

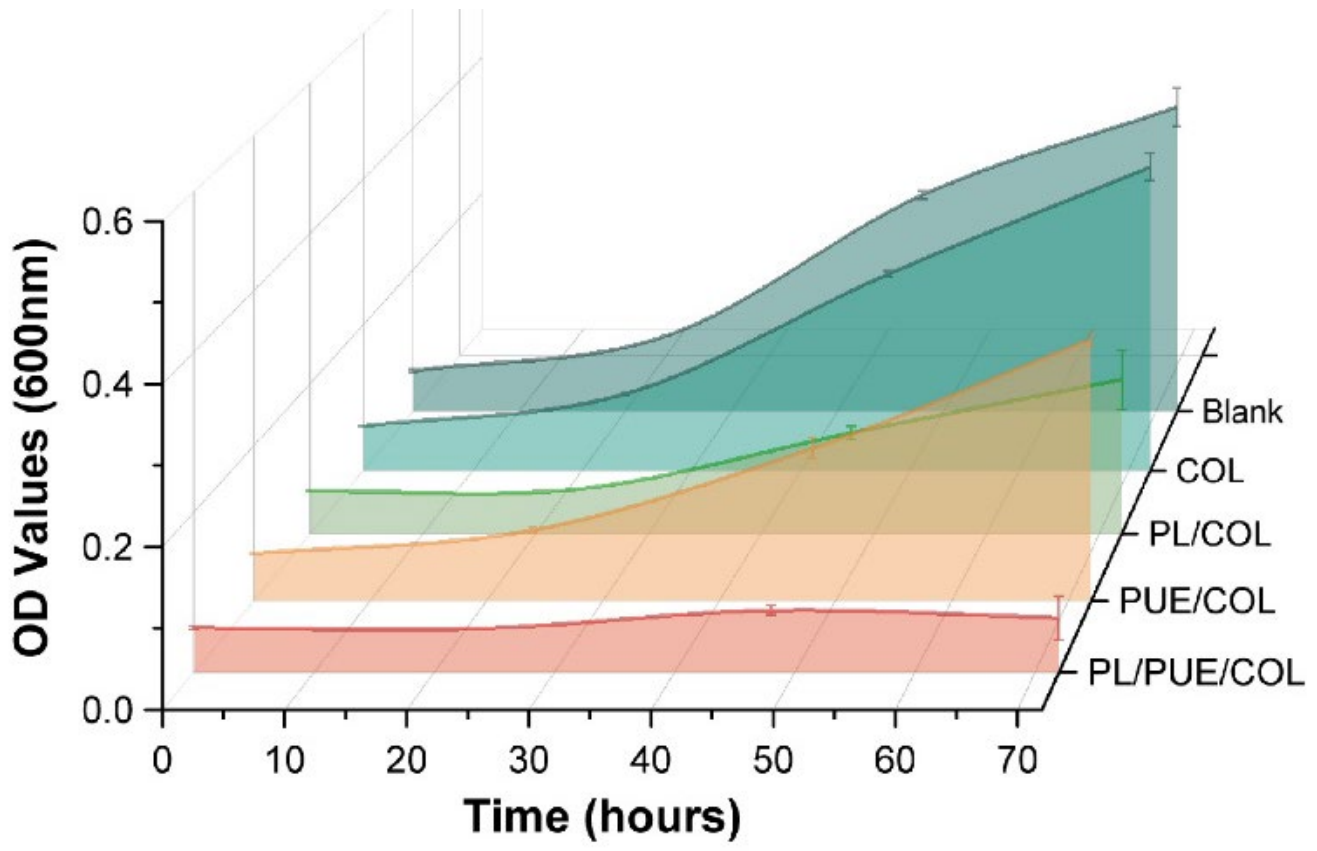


**Supplementary Fig. 12.** Growth curves of *F. nucleatum* cultured with or without different hydrogels over a 72-hour period (*n*=3 independent biological samples). All error bars represent mean ± *SD*. Source data are provided as a Source Data file.

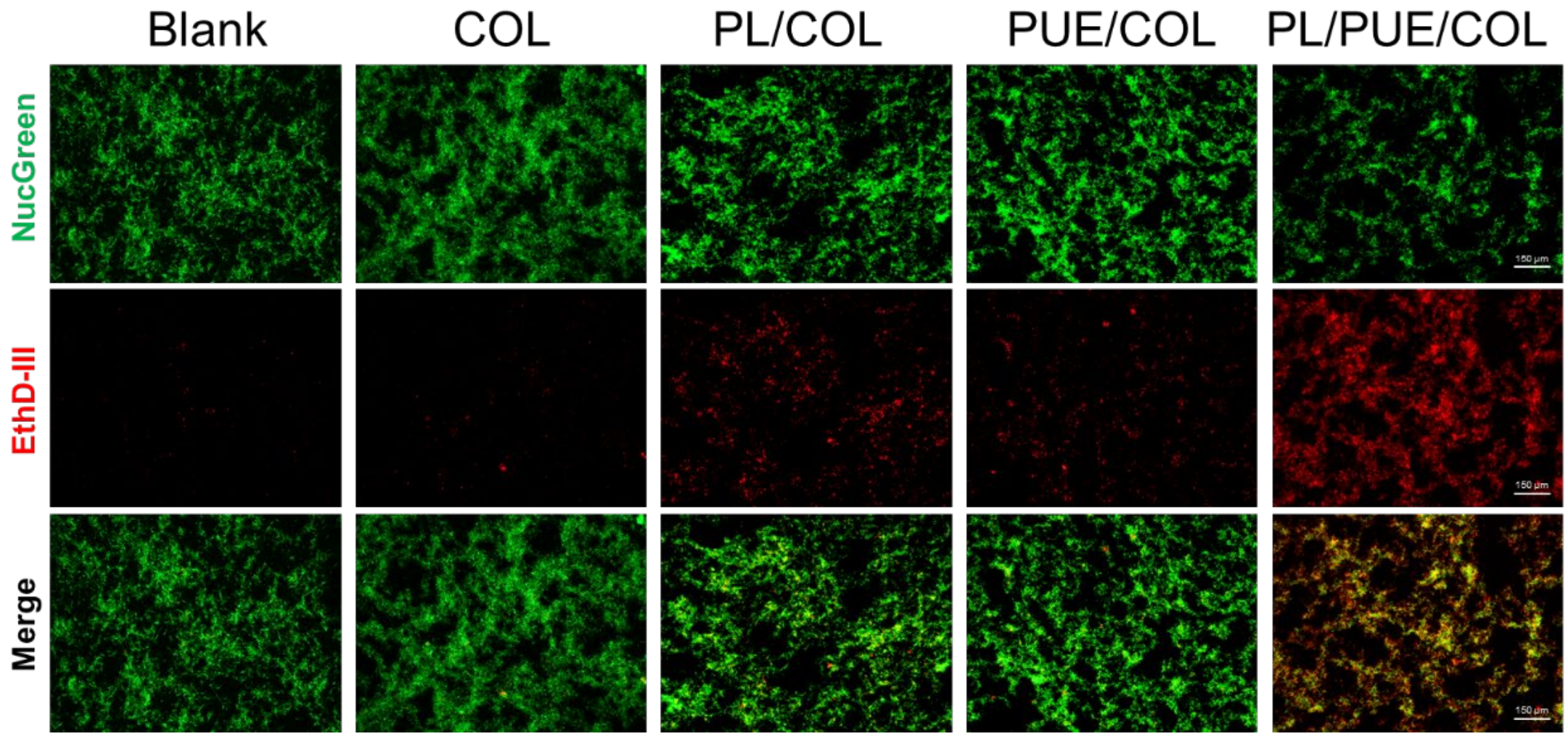


**Supplementary Fig. 13.** Fluorescence images of the treated *F. nucleatum* through Live/Dead staining. The images were representative of three independently repeated experiments from each group.

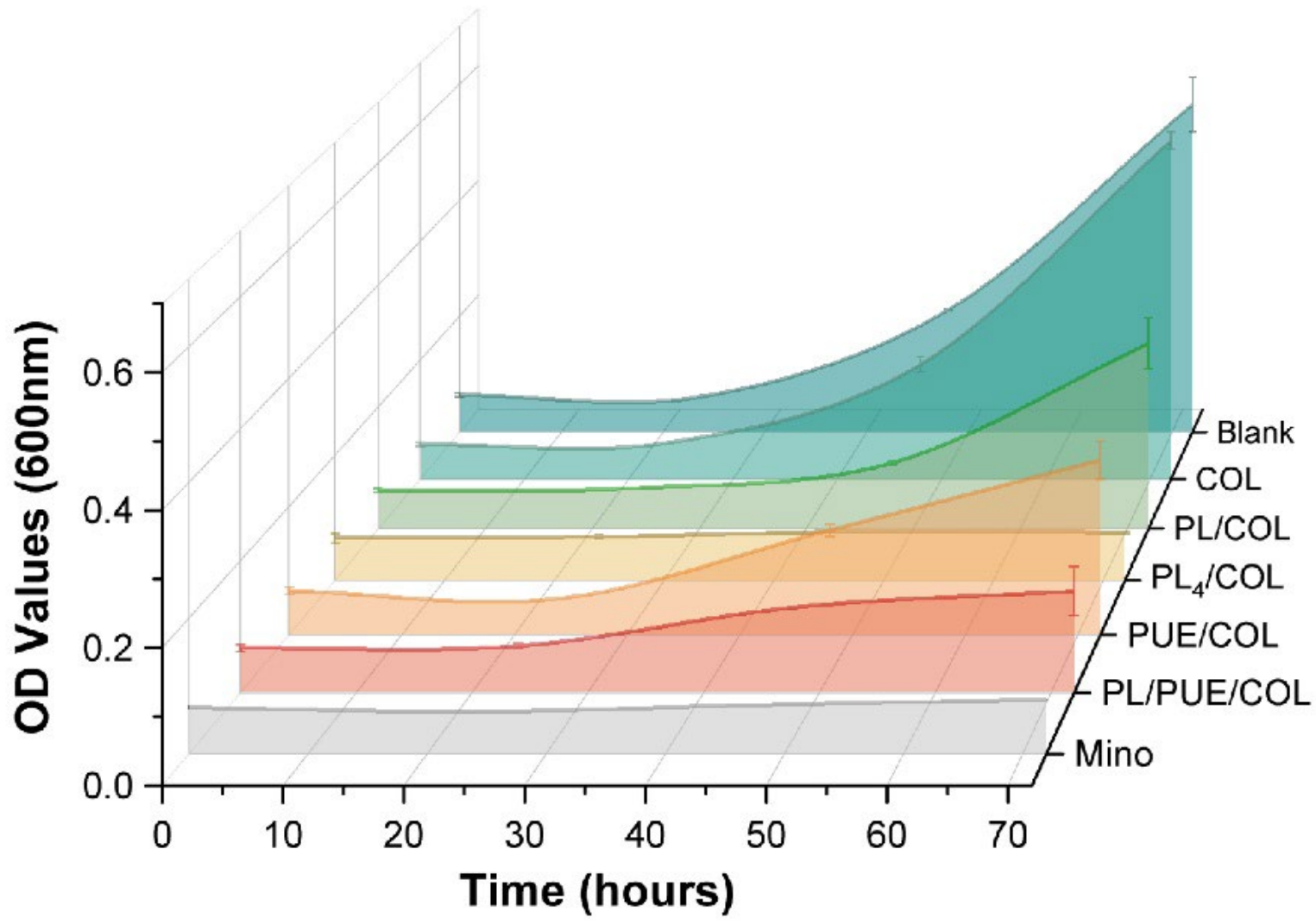


**Supplementary Fig. 14.** Growth curves of mixed infection model of *F. nucleatum* and *P. gingivalis* cultured with or without different hydrogels over a 72-hour period (*n*=3 independent biological samples). All error bars represent mean ± *SD*. Source data are provided as a Source Data file.

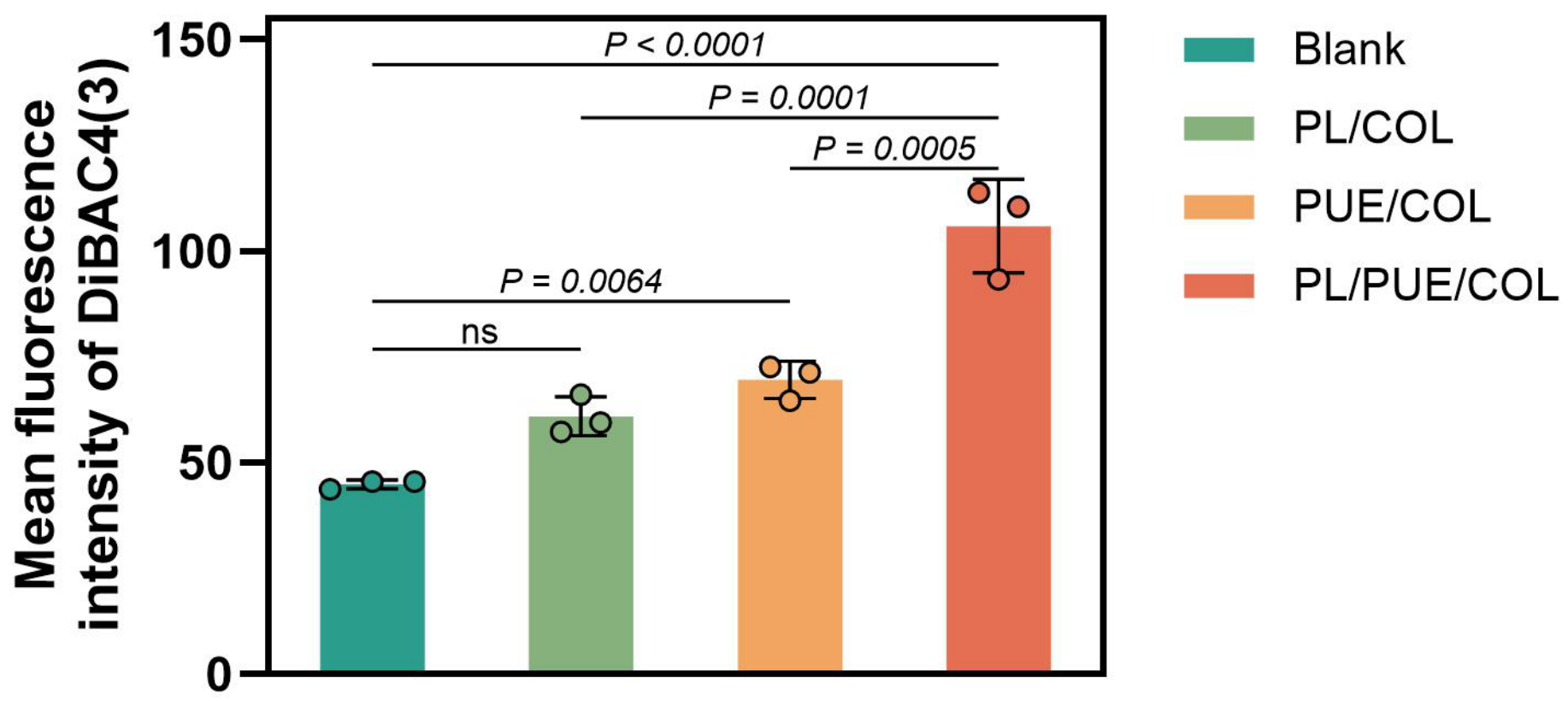


**Supplementary Fig. 15.** Statistical analysis of the relative fluorescence density in Fig. 4h ($n$=3 independent biological samples). All error bars represent mean ± *SD*. *P* values were calculated by one-way ANOVA followed by Tukey's post hoc test (ns, not significant). Source data are provided as a Source Data file.

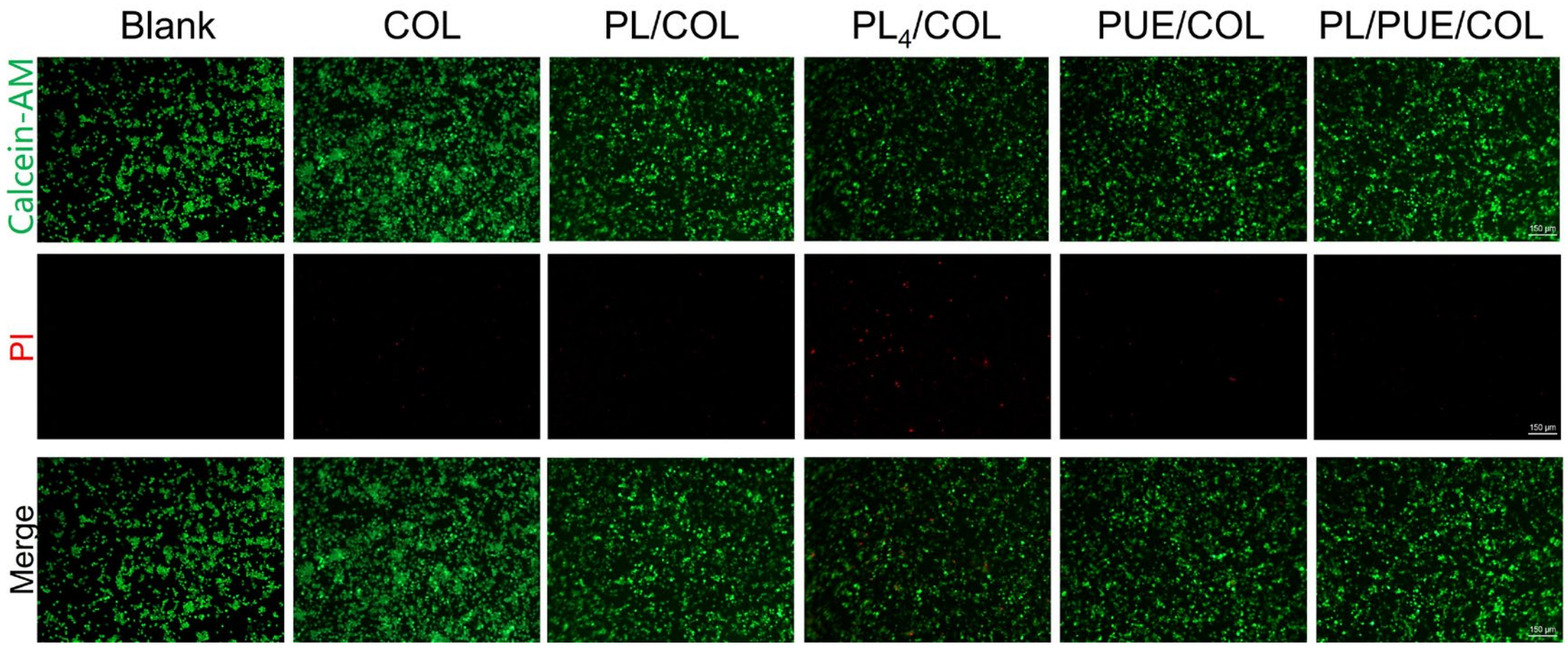


**Supplementary Fig. 16.** Live/Dead staining with Calcein AM (green) and PI (red) indicating live and apoptotic RAW264.7 cells cultured on different hydrogels. The images were representative of three independently repeated experiments from each group.

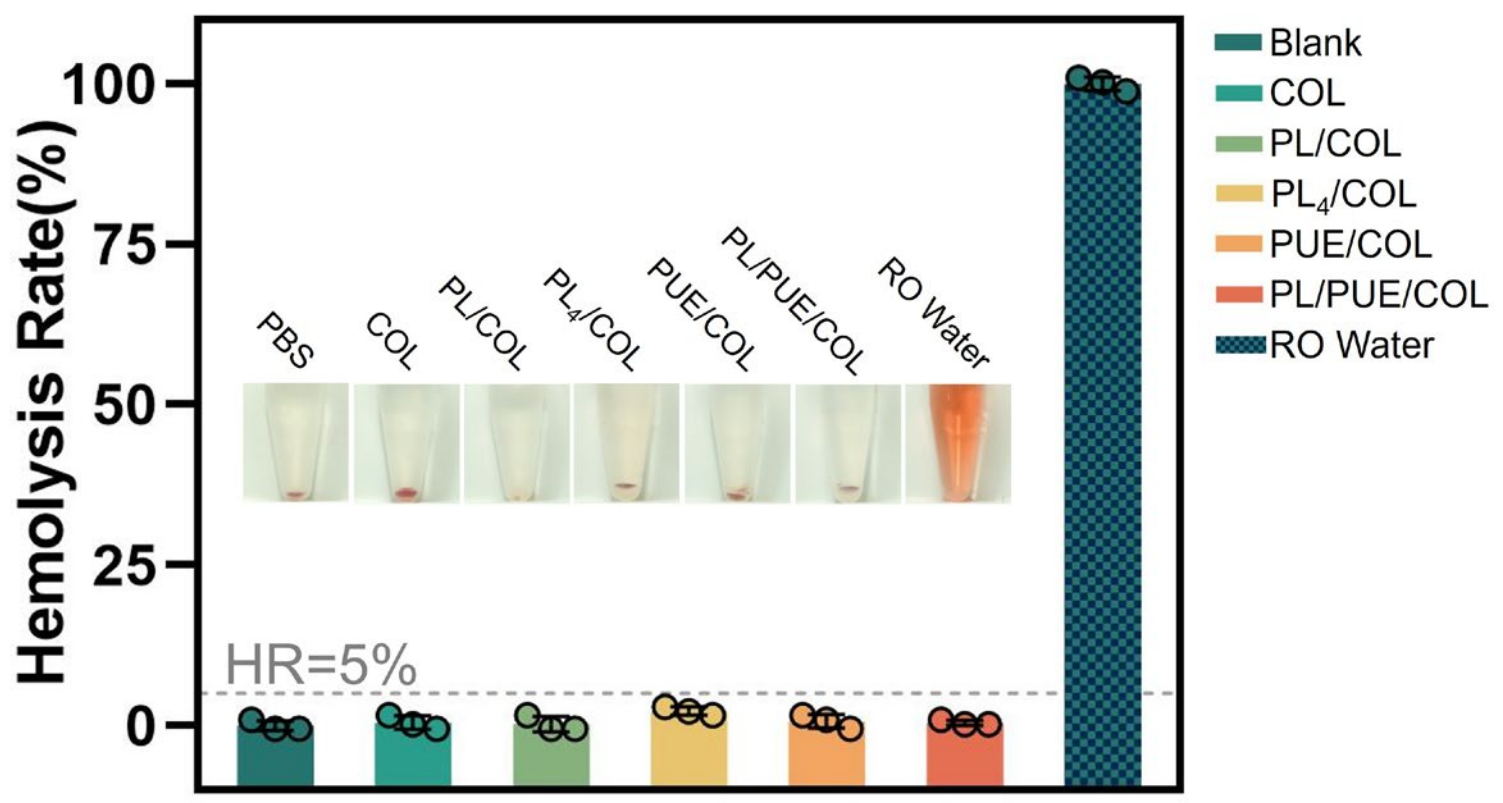


**Supplementary Fig. 17.** The HR values and optical images (*n*=3 independent biological samples). All error bars represent mean ± *SD*. HR hemolysis rate, RO water reverse osmosis water, PBS phosphate-buffered saline. Source data are provided as a Source Data file.

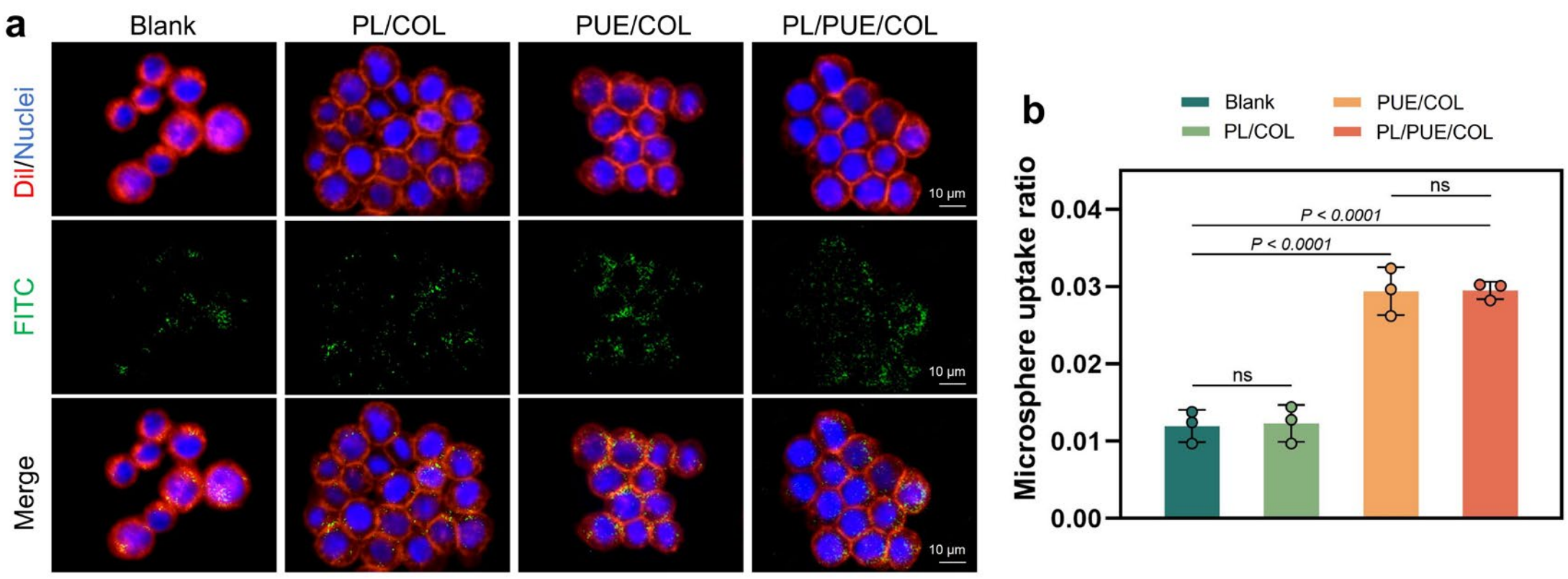


**Supplementary Fig. 18.** (a) Immunofluorescence images and (b) phagocytic rate of phagocytic assay of RAW264.7 cells (*n*=3 independent biological samples). All error bars represent mean ± *SD*. *P* values were calculated by one-way ANOVA followed by Tukey's post hoc test (ns, not significant). Source data are provided as a Source Data file.

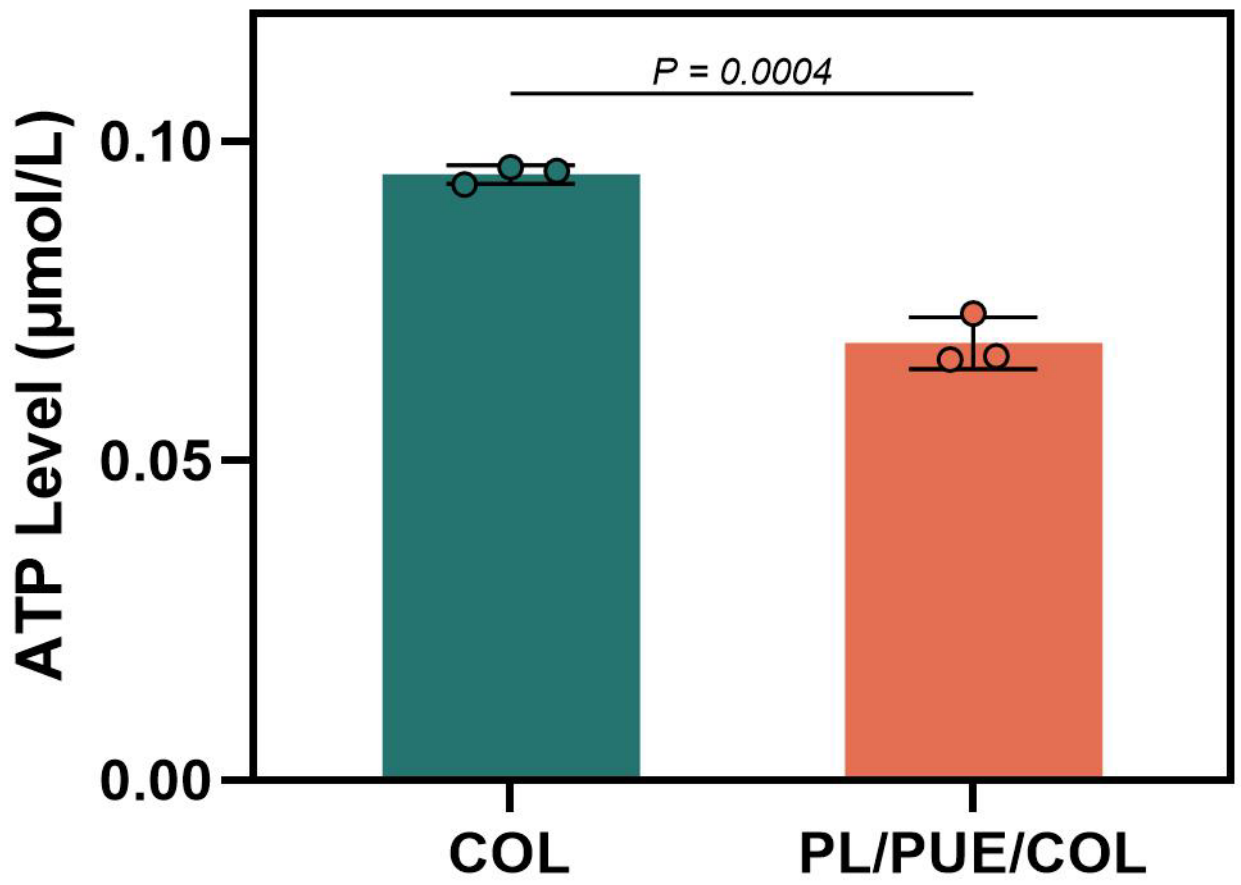


**Supplementary Fig. 19.** The detection of ATP levels in *P. gingivalis* treated with COL and PL/PUE/COL hydrogels (*n*=3 independent biological samples). All error bars represent mean ± *SD*. *P* values were calculated by two-tailed Student's t-test. COL recombinant human type I collagen, PL poly-ε-lysine, PUE puerarin, ATP adenosine triphosphate. Source data are provided as a Source Data file.

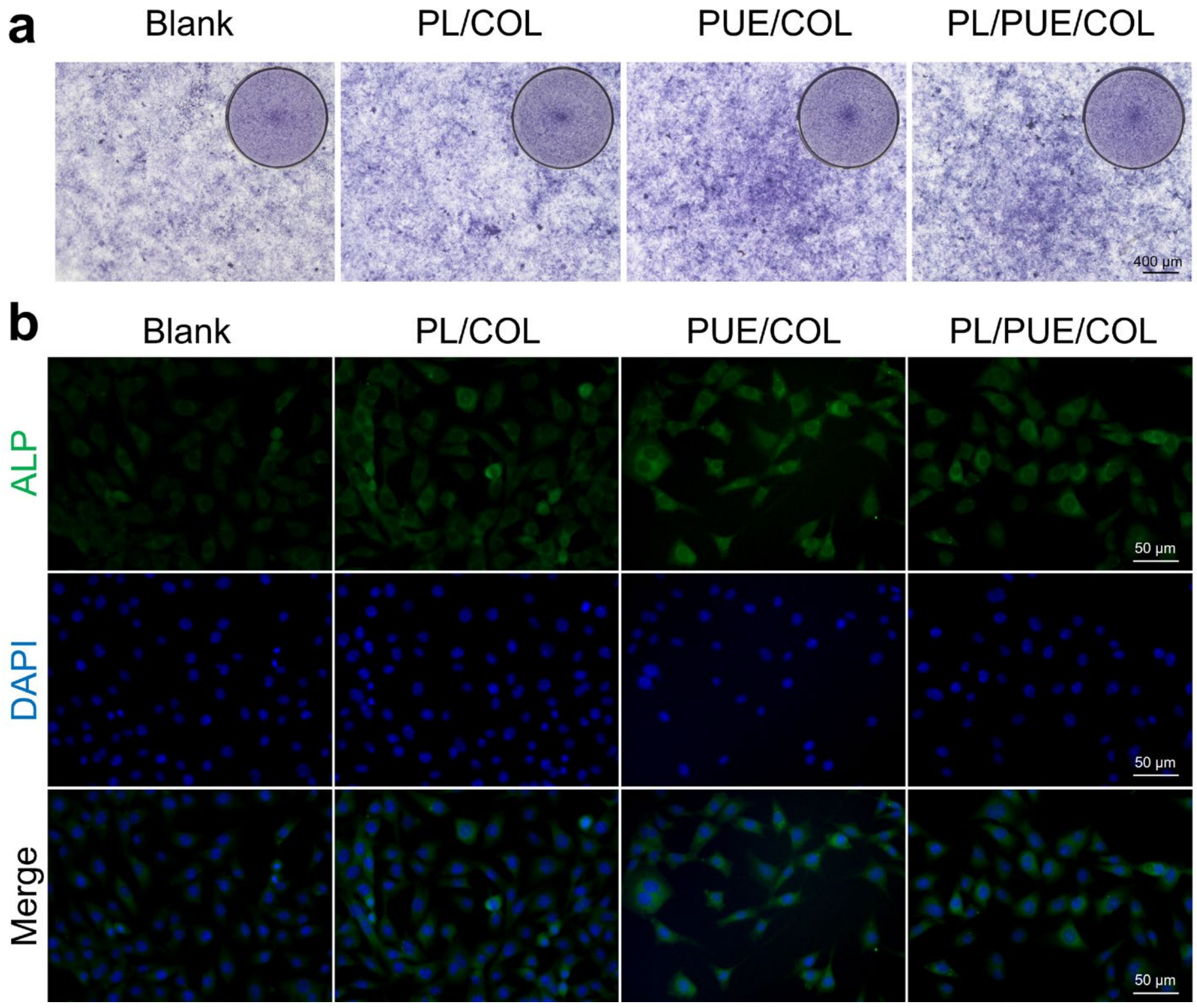


**Supplementary Fig. 20.** Representative images of alkaline phosphatase (ALP) staining and immunofluorescence staining for ALP in MC3T3-E1 cells, treated with PL/COL, PUE/COL, and PL/PUE/COL hydrogels. The images were representative of three independently repeated experiments from each group. COL recombinant human type I collagen, PL poly-ε-lysine, PUE puerarin, ALP alkaline phosphatase.

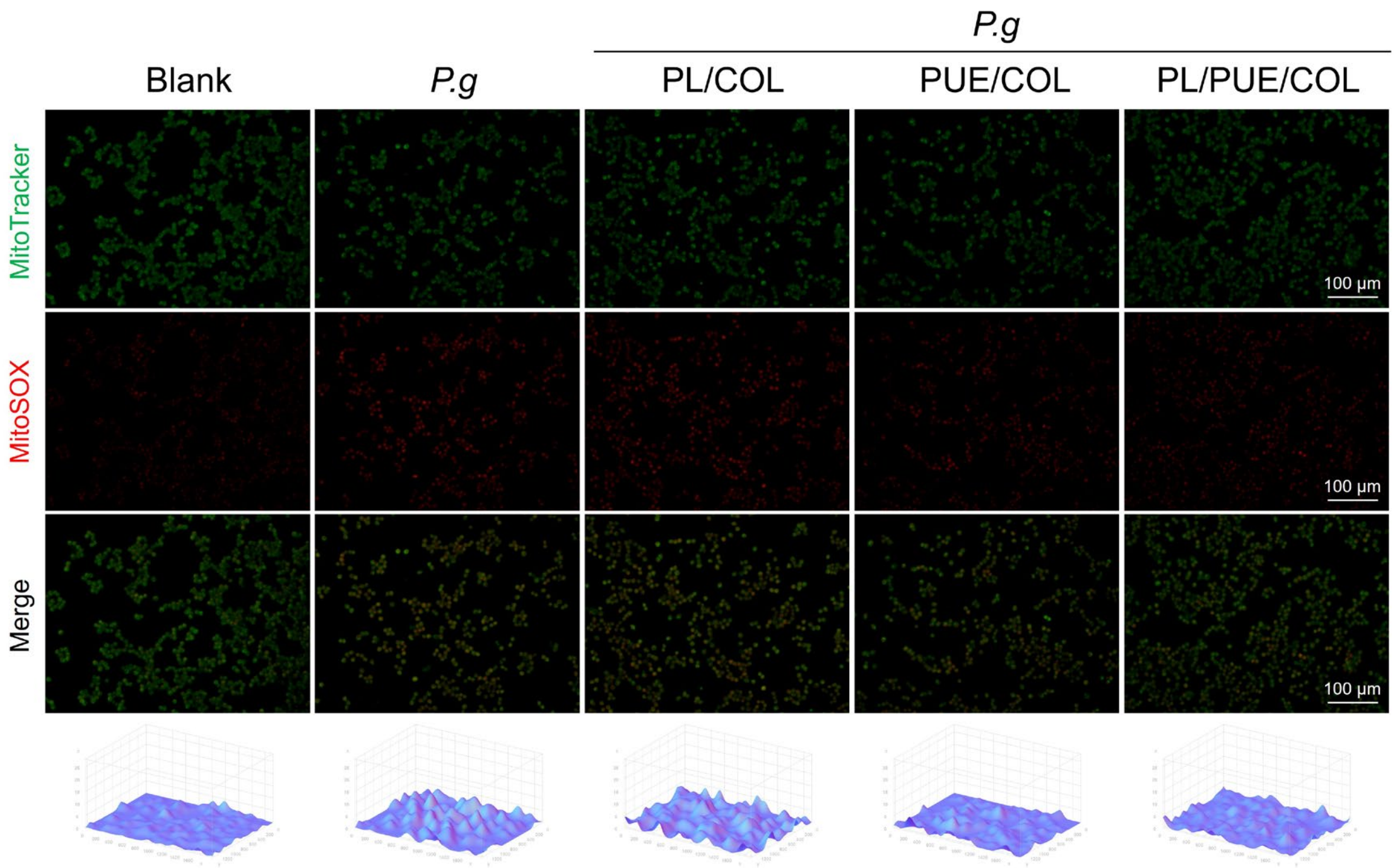


**Supplementary Fig. 21.** Evaluation of mtROS using MitoSOX staining. mtROS mitochondrial reactive oxygen species. The images were representative of three independently repeated experiments from each group.

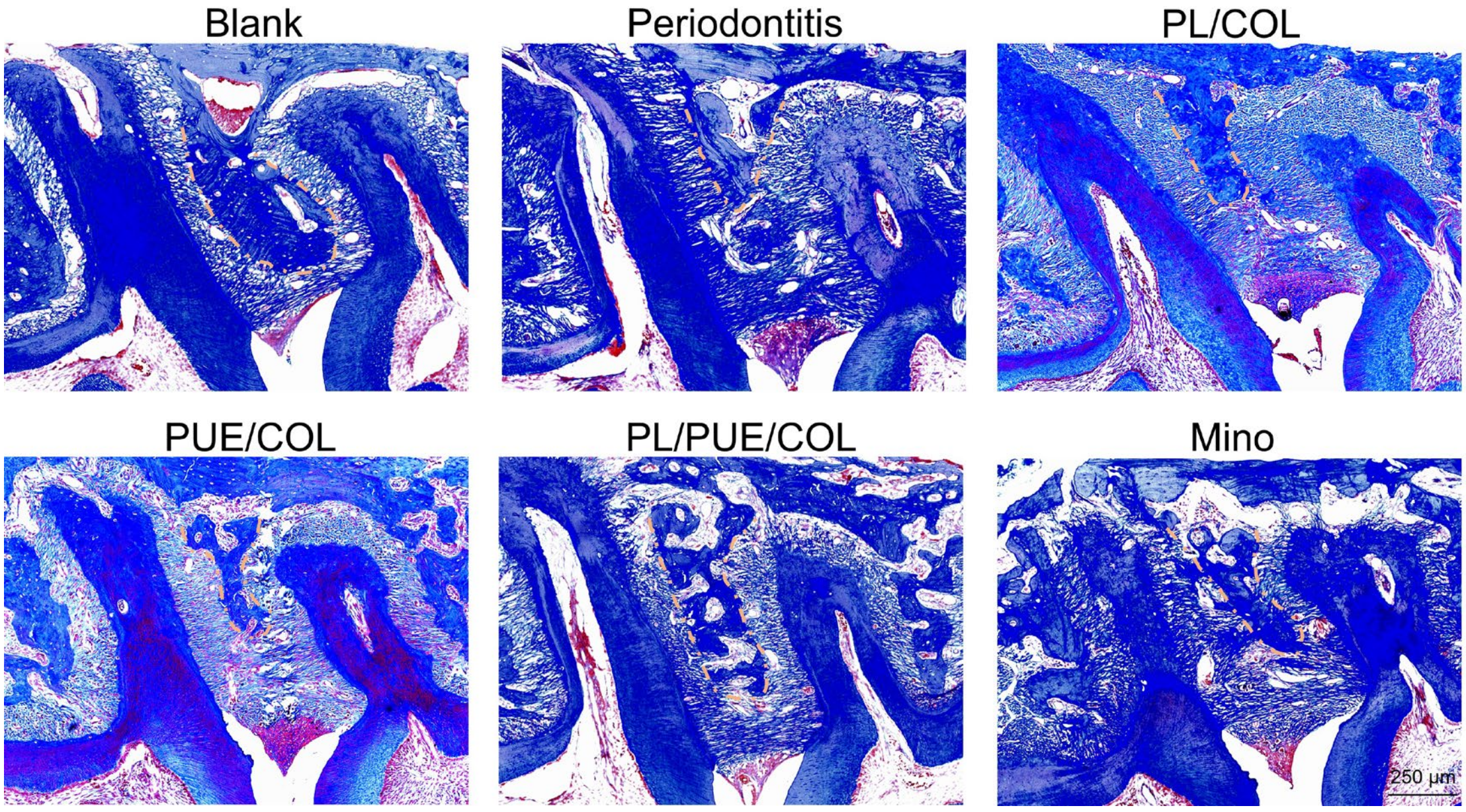


**Supplementary Fig. 22.** Masson staining of the area between the maxillary first and second molars. The images were representative of three independently repeated experiments from each group. Dashed lines indicate the alveolar bone within the lesion area.

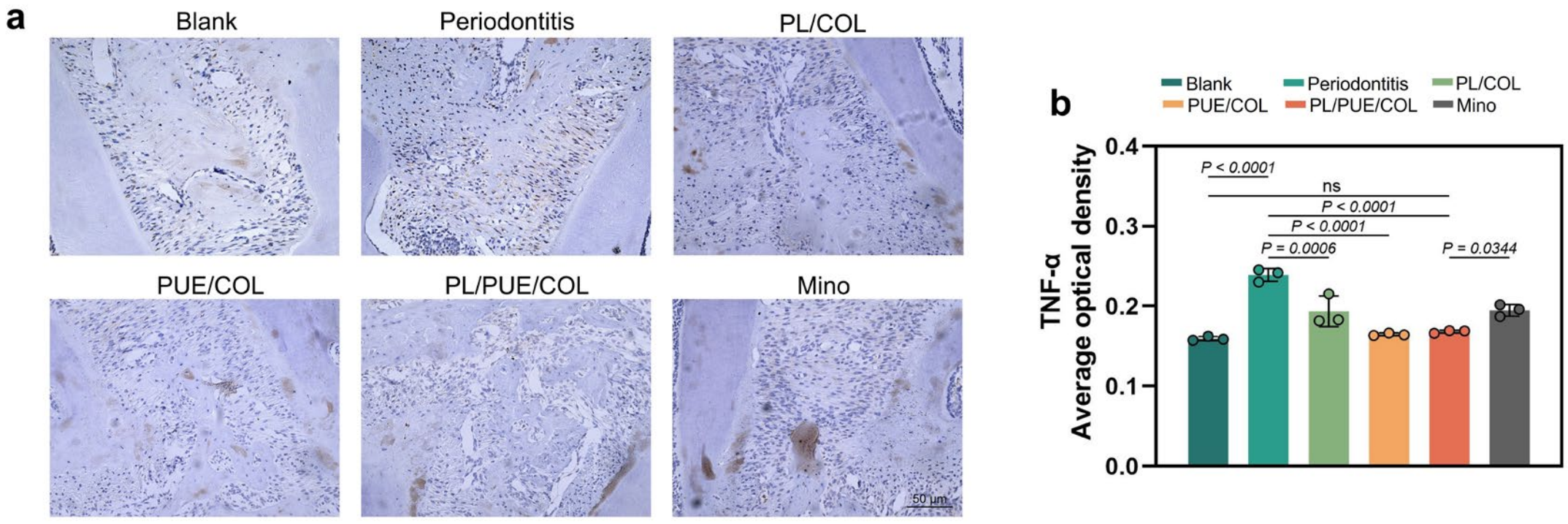


**Supplementary Fig. 23.** (a) Representative images of immunohistochemical staining for TNF-α in periodontal tissue. (b) Semiquantitative analysis of the average optical density (*n*=3 independent biological samples). All error bars represent mean ± *SD*. *P* values were calculated by one-way ANOVA followed by Tukey's post hoc test (ns, not significant). TNF-α tumor necrosis factor-α. Source data are provided as a Source Data file.

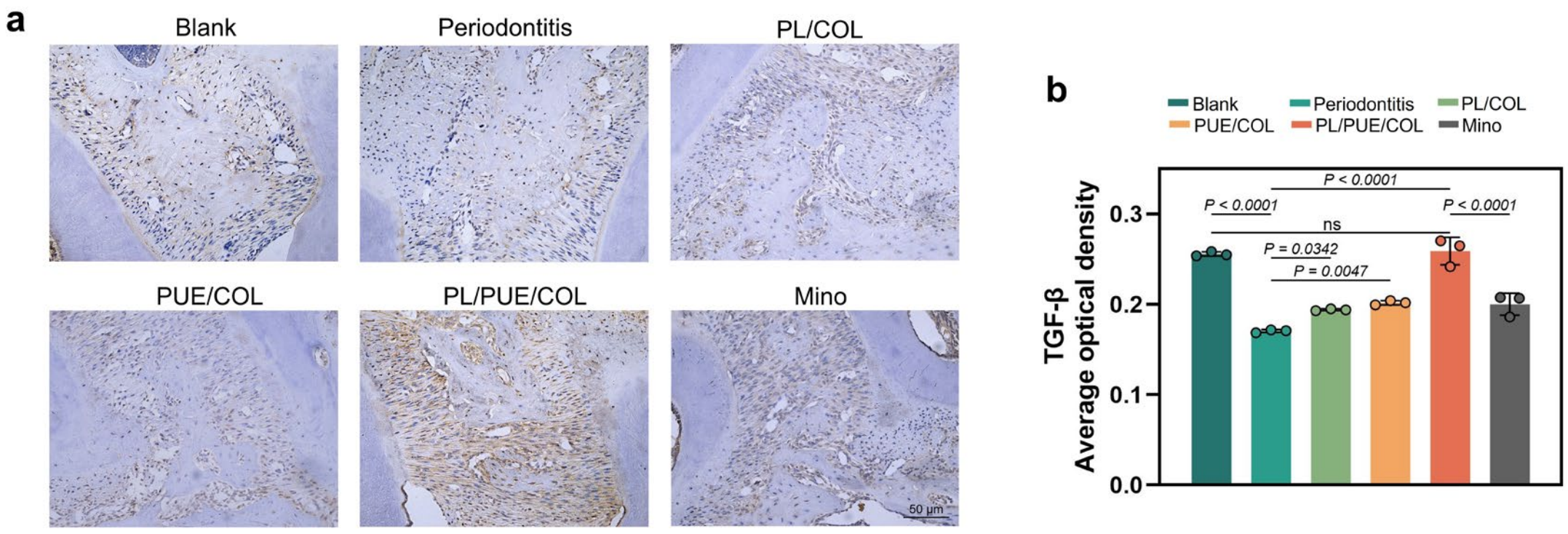


**Supplementary Fig. 24.** (a) Representative images of immunohistochemical staining for TGF-β in periodontal tissue. (b) Semiquantitative analysis of the average optical density (*n*=3 independent biological samples). All error bars represent mean ± *SD*. *P* values were calculated by one-way ANOVA followed by Tukey's post hoc test (ns, not significant). TGF-β transforming growth factor-β. Source data are provided as a Source Data file.

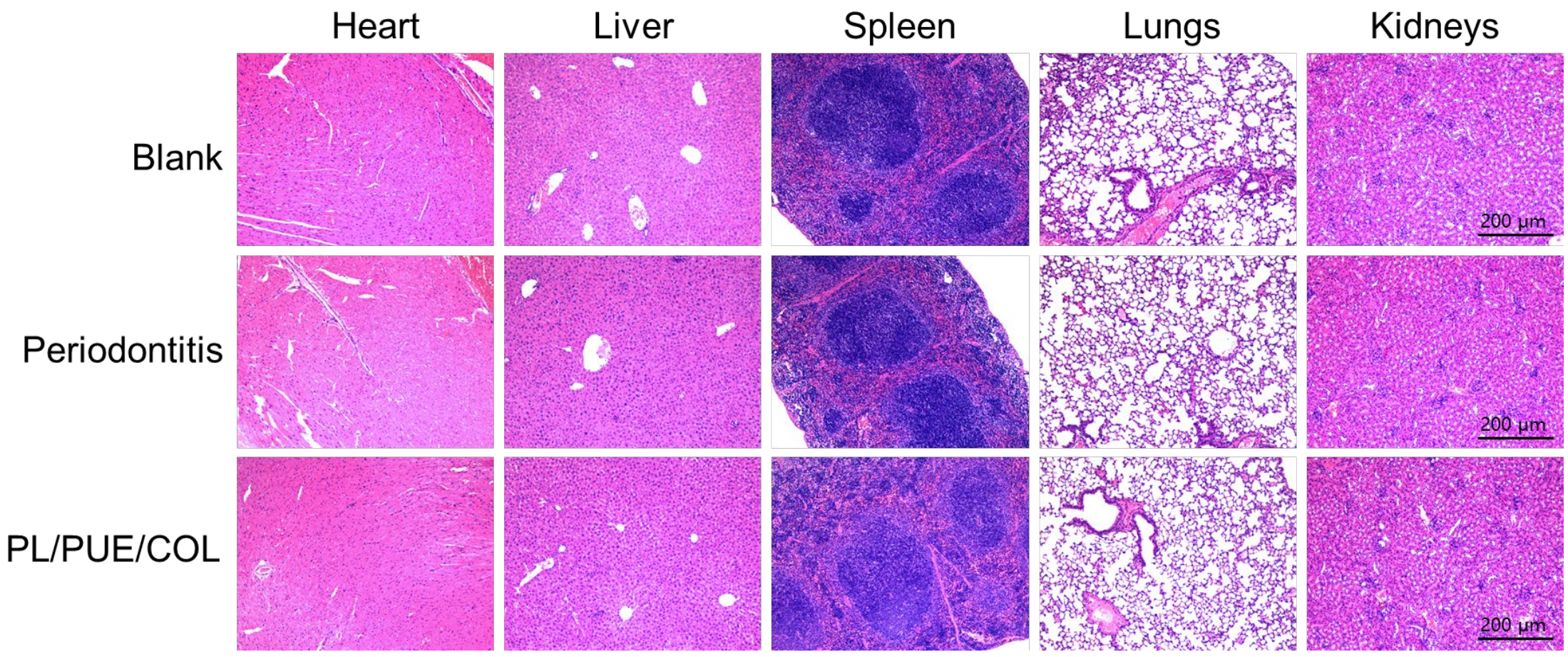


**Supplementary Fig. 25.** HE staining of the main organs of mice after treatment. The images were representative of three independently repeated experiments from each group. HE hematoxylin and eosin.

**Supplementary Table 1** Primer sequences of the genes involved in this study.

| Primer | Forward primer 5′-3′ | Reverse primer 5′-3′ |
|---|---|---|
| *GAPDH* | AAGGCCGGGGCCCACTTGAA | GGACTGTGGTCATGAGCCCTTCCA |
| *iNOS* | GTTCTCAGCCCAACAATACAAGA | GTGGACGGGTCGATGTCAC |
| *CD86* | TCAATGGGACTGCATATCTGCC | GCCAAAATACTACCAGCTCACT |
| *TNF-α* | CAGGCGGTGCCTATGTCTC | CGATCACCCCGAAGTTCAGTAG |
| *IL-1β* | GAAATGCCACCTTTTGACAGTG | TGGATGCTCTCATCAGGACAG |
| *TGF-β* | GAGCCCGAAGCGGACTACTA | TGGTTTTCTCATAGATGGCGTTG |
| *LAMP1* | AGCATACCGGTGTGTCAGTG | GTTGGGGAAGGTCCATCCTG |
| *CTSD* | ACCTCTGGCTTTAAGCTTTGCT | TAATCGCGAAGGAGGACGAAG |
