# Supplementary material for: Supramolecular strategy for compartment pathogen clearance and immuno-metabolic homeostasis to treat periodontitis
Source: Nat Commun. 2026 Jun 18;17:7714. doi: 10.1038/s41467-026-74544-5 (PMC13434047; doi:10.1038/s41467-026-74544-5)
Supplement: Supplementary file 2 [file 41467_2026_74544_MOESM2_ESM.pdf]

nature portfolio

Corresponding author(s): Dingming Huang and Zhenming Wang

Last updated by author(s): May 28, 2026

Reporting Summary

Nature Portfolio wishes to improve the reproducibility of the work that we publish. This form provides structure for consistency and transparency in reporting. For further information on Nature Portfolio policies, see our Editorial Policies and the Editorial Policy Checklist.

Statistics

For all statistical analyses, confirm that the following items are present in the figure legend, table legend, main text, or Methods section.

n/a	Confirmed	
☐	☒	The exact sample size (*n*) for each experimental group/condition, given as a discrete number and unit of measurement
☐	☒	A statement on whether measurements were taken from distinct samples or whether the same sample was measured repeatedly
☐	☒	The statistical test(s) used AND whether they are one- or two-sided<br>*Only common tests should be described solely by name; describe more complex techniques in the Methods section.*
☒	☐	A description of all covariates tested
☐	☒	A description of any assumptions or corrections, such as tests of normality and adjustment for multiple comparisons
☐	☒	A full description of the statistical parameters including central tendency (e.g. means) or other basic estimates (e.g. regression coefficient) AND variation (e.g. standard deviation) or associated estimates of uncertainty (e.g. confidence intervals)
☐	☒	For null hypothesis testing, the test statistic (e.g. *F*, *t*, *r*) with confidence intervals, effect sizes, degrees of freedom and *P* value noted<br>*Give P values as exact values whenever suitable.*
☒	☐	For Bayesian analysis, information on the choice of priors and Markov chain Monte Carlo settings
☒	☐	For hierarchical and complex designs, identification of the appropriate level for tests and full reporting of outcomes
☒	☐	Estimates of effect sizes (e.g. Cohen's *d*, Pearson's *r*), indicating how they were calculated

Our web collection on statistics for biologists contains articles on many of the points above.

Software and code

Policy information about availability of computer code

Data collection	No custom code or software was used for data collection.
Data analysis	Statistical analysis and data visualization were performed using SPSS 22.0, GraphPad Prism 8, and Origin 2024. Image quantification was conducted using Fiji/ImageJ software. Micro-CT 3D reconstruction and quantification were performed using Mimics Medical v20.0. Molecular dynamics simulations and analyses were conducted using Gaussian 16 A.03, Multiwfn 3.8(dev), acpype, GROMACS 2021.7, and VMD 1.9.3.

For manuscripts utilizing custom algorithms or software that are central to the research but not yet described in published literature, software must be made available to editors and reviewers. We strongly encourage code deposition in a community repository (e.g. GitHub). See the Nature Portfolio guidelines for submitting code & software for further information.

Data

Policy information about availability of data

All manuscripts must include a data availability statement. This statement should provide the following information, where applicable:

- Accession codes, unique identifiers, or web links for publicly available datasets
- A description of any restrictions on data availability
- For clinical datasets or third party data, please ensure that the statement adheres to our policy

The data that support the findings of this study are available within the paper and its Supplementary Information files. The initial coordinates of the COL model used in this study are available in the Protein Data Bank under accession code 1K6F [http://doi.org/10.2210/pdb1K6F/pdb]. Raw RNA sequencing data generated in

this study have been deposited in the Genome Sequence Archive (GSA) database under accession number CRA041814 [https://ngdc.cncb.ac.cn/gsa/browse/CRA041814]. Source data are provided with this paper.

Research involving human participants, their data, or biological material

Policy information about studies with human participants or human data. See also policy information about sex, gender (identity/presentation), and sexual orientation and race, ethnicity and racism.

Reporting on sex and gender	This study does not involve human research participants.
Reporting on race, ethnicity, or other socially relevant groupings	This study does not involve human research participants.
Population characteristics	This study does not involve human research participants.
Recruitment	This study does not involve human research participants.
Ethics oversight	This study does not involve human research participants.

Note that full information on the approval of the study protocol must also be provided in the manuscript.

Field-specific reporting

Please select the one below that is the best fit for your research. If you are not sure, read the appropriate sections before making your selection.

☒ Life sciences ☐ Behavioural & social sciences ☐ Ecological, evolutionary & environmental sciences

For a reference copy of the document with all sections, see nature.com/documents/nr-reporting-summary-flat.pdf

Life sciences study design

All studies must disclose on these points even when the disclosure is negative.

Sample size	Sample sizes (n = 3 for both in vitro assays and in vivo animal experiments) were chosen based on standard practices in similar biomaterial and periodontitis models in previous studies. This approach strictly aligns with the "Reduction" principle of the 3Rs for animal welfare, as these minimal sample sizes have been previously shown to be sufficient for evaluating statistical significance using standard parametric tests.
Data exclusions	No data were excluded from the analyses.
Replication	All biological experiments were performed in at least three independent replicates ($n \geq 3$) to ensure reproducibility. All attempts at replication were successful.
Randomization	For in vivo studies, C57BL/6 mice were randomly allocated into the different experimental and control groups prior to the induction of periodontitis and subsequent treatments. For in vitro studies, cell culture wells and bacterial samples were randomly assigned to different treatment groups.
Blinding	Investigators were not blinded to group allocation during experimental execution and data collection, as knowledge of the specific hydrogel formulations was required for accurate administration. However, subsequent data analyses were performed objectively using standardized software (e.g., Mimics Medical, ImageJ) to minimize bias.

Reporting for specific materials, systems and methods

We require information from authors about some types of materials, experimental systems and methods used in many studies. Here, indicate whether each material, system or method listed is relevant to your study. If you are not sure if a list item applies to your research, read the appropriate section before selecting a response.

Materials & experimental systems

n/a	Involved in the study
☐	☒ Antibodies
☐	☒ Eukaryotic cell lines
☒	☐ Palaeontology and archaeology
☐	☒ Animals and other organisms
☒	☐ Clinical data
☒	☐ Dual use research of concern
☒	☐ Plants

Methods

n/a	Involved in the study
☒	☐ ChIP-seq
☒	☐ Flow cytometry
☒	☐ MRI-based neuroimaging

Antibodies

Antibodies used	Primary antibodies used include: anti-iNOS primary antibody (1:100 dilution, ER1706-89, Huabio, China); anti-CD206 primary antibody (1:400 dilution, ET1702-04, Huabio, China); anti-ALP primary antibody (1:100 dilution, ET1601-21, Huabio, China); anti-TGF-β (1:300 dilution, GB115739, Servicebio, China), and anti-TNF-α primary antibody (1:500 dilution, GB115702, Servicebio, China). Secondary antibodies used include: Alexa Fluor® Plus 488 Goat anti-Rabbit secondary antibody (1:1000 dilution, A32731, Invitrogen, USA).
Validation	All antibodies used in this study are commercially available and have been routinely validated by their respective manufacturers (Huabio, Invitrogen and Servicebio) for the designated applications (immunofluorescence and immunohistochemistry) and species reactivity (mouse). Validation data are available on the manufacturers' websites (Huabio: www.huabio.cn; Invitrogen: www.fishersci.com/us/en/brands/IIAM0WMR/invitrogen; Servicebio: www.servicebio.cn.)

Eukaryotic cell lines

Policy information about cell lines and Sex and Gender in Research

Cell line source(s)	The RAW 264.7 murine macrophage cell line and the MC3T3-E1 murine pre-osteoblastic cell line were both obtained from the American Type Culture Collection (ATCC).
Authentication	The cell lines were authenticated by the supplier (ATCC) prior to distribution using standard authentication procedures. No further authentication was performed in our laboratory.
Mycoplasma contamination	All cell lines were routinely tested and confirmed to be negative for mycoplasma contamination during the experimental period.
Commonly misidentified lines (See ICLAC register)	No commonly misidentified cell lines defined by the ICLAC register were used in this study.

Animals and other research organisms

Policy information about studies involving animals; ARRIVE guidelines recommended for reporting animal research, and Sex and Gender in Research

Laboratory animals	6-week-old male C57BL/6 mice weighing between 18-20 g were used to establish the in vivo periodontitis model. Mice were housed in a specific pathogen free facility at 20-22? and 40-60% humidity on a 12-hour light/dark cycle, with ad libitum access to standard chow and water.
Wild animals	This study did not involve wild animals.
Reporting on sex	Only male mice were used in this study. This was specifically designed to exclude the potential confounding effects of fluctuating estrogen levels and estrous cycles present in female mice, which can significantly influence bone metabolism, immune inflammatory responses, and alveolar bone resorption, thereby ensuring the stability and reproducibility of the periodontitis model.
Field-collected samples	This study did not involve field-collected samples.
Ethics oversight	All animal experiments and surgical procedures were reviewed and approved by the Ethics Committee of West China Hospital of Stomatology, Sichuan University (Registration No. WCHSIRB-D-2025–075).

Note that full information on the approval of the study protocol must also be provided in the manuscript.

Plants

Seed stocks	N/A
Novel plant genotypes	N/A
Authentication	N/A
