# Supplementary material for: Supramolecular strategy for compartment pathogen clearance and immuno-metabolic homeostasis to treat periodontitis
Source: Nat Commun. 2026 Jun 18;17:7714. doi: 10.1038/s41467-026-74544-5 (PMC13434047; doi:10.1038/s41467-026-74544-5)
Supplement: Supplementary file 3 [file 41467_2026_74544_MOESM3_ESM.pdf]

nature portfolio

Peer Review File

Supramolecular Strategy for Compartment Pathogen Clearance and Immuno-Metabolic Homeostasis to Treat Periodontitis

Corresponding Author: Dr Zhenming Wang

This file contains all reviewer reports in order by version, followed by all author rebuttals in order by version.

Version 1:

Reviewer comments:

Reviewer #1

(Remarks to the Author)
The paper entitled “A Supramolecular Strategy Orchestrates Dual-Compartment Pathogen Clearance and Immuno-Metabolic Homeostasis to Reverse Periodontitis” designed an injectable supramolecular hydrogel for treating intracellular infections and restoring tissue homeostasis in periodontitis. This paper provides innovative approaches to enhance intracellular bacterial clearance and host immune homeostasis. I have concerns about the followed specific analyses before publication, and a minor revision is needed:
1. Although FTIR and DSC provide some clues regarding intermolecular interactions, these characterizations are too macroscopic. It is recommended to supplement NMR spectroscopy to observe the chemical shift changes of specific functional groups on PUE, thereby confirming its specific non-covalent binding with COL.
2. The application of this supramolecular complex is in the oral environment of periodontitis. Can these three components form and maintain a stable supramolecular structure in a complex physiological fluid environment? Will PUE precipitate again? It is suggested to supplement assembly experiments in physiological fluids to verify whether PUE dissociates.
3. Expressing the antimicrobial effect as "Antibacterial rate (%)" in Figure 3B is non-standard. In microbiology, a 90% antibacterial rate merely represents a 1-log reduction in CFU, which is considered ineffective in the field of antibacterial materials. A true antibacterial effect requires at least a 3-log (99.9%) reduction. Please change the y-axis of all relevant figures to CFU counts or Log-transformed values.
4. Periodontitis is caused by highly complex polymicrobial biofilms rather than single planktonic bacteria. The authors only tested single P. gingivalis and F. nucleatum models. It is recommended that the authors establish an in vitro dual-species or multi-species infection model to verify the clearance capability of this supramolecular system in a complex infection.
5. Although P. gingivalis is a key pathogen (Gram-negative), periodontal infections are often accompanied by Gram-positive bacteria. The mechanism of PL primarily targets the LPS-rich outer membrane. Since Gram-positive bacteria lack an outer membrane but have a very thick peptidoglycan layer, does this supramolecular hydrogel still possess synergistic bactericidal effects against them?
6. In validating the anti-inflammatory properties, LPS was used to stimulate RAW264.7 cells to evaluate macrophage immune behavior. However, in subsequent experiments, this was switched to live P. gingivalis infection. There is a lack of necessary logical transition; please explain the specific rationale for switching from LPS to P. gingivalis.
7. It is recommended to supplement experiments to demonstrate the restoration of lysosomal activity.
8. Typically, macrophages eradicate engulfed bacteria by producing large amounts of ROS. As a strong antioxidant, while PUE clears intracellular ROS, will it unavoidably weaken the macrophages' defense of using ROS for bactericidal activity? The authors need to provide a more discussion on this complex redox balance.
9. Conditioned medium was used to demonstrate the promotion of osteogenesis following immune modulation. However, do the PUE or PL released from the hydrogel possess direct osteogenic effects themselves? It is recommended to supplement an experiment directly co-culturing the hydrogel extract with osteoblasts.
10. The PL4/COL group exhibits toxicity, whereas the toxicity of PL/PUE/COL is significantly reduced. Is this simply because the PL concentration was halved, or is it because the addition of PUE buffered the cationic toxicity of PL?
11. For the in vivo experiments, the Masson staining and immunohistochemistry results need to be completed by including the PL/COL, PUE/COL, and Mino comparison groups.
12. The "Dual Compartment" concept is a core concept in the title of the article, but it is not adequately reflected in the Discussion section.

13. This work design has significant value in application and translational research, and warrants in-depth and systematic investigation to develop a distinctive framework. Thus, it will be better if the authors discuss its limitation in current study. This will really strengthen their study and will help the readers understand more about their future study of the supramolecular materials.
14. There are inconsistencies in multiple places throughout the text; for example, "MitoSOX" and "Heatmap" appear in different formats in different sections.
15. There is a formatting error in Reference [69]; please correct it.

Reviewer #2

(Remarks to the Author)
This study presents a highly innovative and well-executed investigation on the tharapy of periodontitis by developing an injectable supramolecular hydrogel (PL/PUE/COL) through the co-assembly of collagen, poly-ε-lysine,and puerarin. Authors addresses a critical challenge in periodontitis therapy: the persistence of intracellular bacteria that evade immune clearance and induce lysosomal dysfunction. Overall, the study is comprehensive, spanning materials design, mechanistic elucidation, and in vivo validation. In my opinion, after addressing the following minor concerns on presentation, methodology, discussion and references, it is suitable for publication.

1. In the Abstract and Conclusion, the “dual-compartment” concept is clearly described. However, it would be beneficial to emphasize that the restoration of lysosomal homeostasis serves as the mechanistic bridge between extracellular bacterial reduction and intracellular immune reprogramming. This would further integrate the two compartments into a unified therapeutic axis.
2. Define all abbreviations (e.g., MTG, CEJ-ABC, BV/TV, TCIRG1, LAMP1, CTSD) at first appearance in the main text and figure legends.
3. Add brief explanatory notes for Supplementary Figures 1-18 in the main text.
4. Supplement strain, sex, age, weight of mice used in the in vivo model.
5. Add cell source, species, and passage number for RAW264.7 and MC3T3-E1 cells.
6. In Figure 3H, the 3D surface plots are visually informative. Please supplement this with a quantitative bar graph of fluorescence intensity to allow for direct statistical comparison of membrane depolarization between groups.
7. In Figure 6G (Lysosome-bacteria colocalization), authors are suggested to add a quantitative colocalization analysis (e.g., Pearson’s or Mander’s coefficient) to support the visual observation that PUE/COL and PL/PUE/COL restore phagolysosomal fusion.
8. Please standardize descriptions of anaerobic culture conditions (gas mixture, time, temperature).
9. In the periodontitis model, periodontal ligament stem cells (PDLSCs) play a critical role in tissue remodeling. Could you evaluate the effects of PUE, PUE/COL, and PL/PUE/COL on the osteogenic activity of PDLSCs?
10. Please supplement the data on the in vitro antioxidant capacity of PUE and its composite materials.
11. Please supplement the Masson staining and immunohistochemistry results of the PL/COL, PUE/COL, and Mino comparison groups.
12. PUE and PL demonstrate a significant synergistic antibacterial effect, which is a very interesting phenomenon. I was wondering, does PL exhibit similar effects with other polyphenolic small molecules? Have you considered conducting related research or planning studies in this area?
13. The data in this paper is relatively comprehensive and the logic is clear, but I would like to know the current limitations of this work. I suggest the author add a discussion on the shortcomings of this study and the issues that need to be addressed in the future.
14. Some latest studies for the therapy of the periodontitis are suggested to be compared with current work in discussion section to highlight the advantages of current strategy such as Bioactive Materials 2025,53:72-83, ACS Nano 2025,19,13341-13355, and Journal of the American Chemical Society 2021,143,15427-15439, but not limited to them.

Version 2:

Reviewer comments:

Reviewer #1

(Remarks to the Author)
The authors have addressed all my comments, and this manuscript is recommended to be accepted.

Reviewer #2

(Remarks to the Author)
In the revised paper, authors have revised the paper carefully and addressed all the issues raised by reviewers. It can be published as is.

Open Access This Peer Review File is licensed under a Creative Commons Attribution 4.0 International License, which permits use, sharing, adaptation, distribution and reproduction in any medium or format, as long as you give appropriate credit to the original author(s) and the source, provide a link to the Creative Commons license, and indicate if changes were made.
In cases where reviewers are anonymous, credit should be given to 'Anonymous Referee' and the source.
The images or other third party material in this Peer Review File are included in the article's Creative Commons license, unless indicated otherwise in a credit line to the material. If material is not included in the article's Creative Commons license and your intended use is not permitted by statutory regulation or exceeds the permitted use, you will need to obtain permission directly from the copyright holder.
To view a copy of this license, visit https://creativecommons.org/licenses/by/4.0/

Point-by-Point Response

Manuscript ID number:

NCOMMS-26-004981

Manuscript title:

A Supramolecular Strategy Orchestrates Dual-Compartment Pathogen Clearance and Immuno-Metabolic Homeostasis to Reverse Periodontitis

The revised manuscript is attached and the places being changed have been marked with **yellow highlights.** Comments are shown below in *italics*. Our response is given in normal font and blue text.

Point-by-Point Responses to the Comments on Manuscript:

1. *We notice that some internal controls are missing from testing.*

Response:

We fully agree that a complete set of internal controls is crucial to rigorously isolate the synergistic effects of our ternary supramolecular system. We have now refined our experimental design to include a comprehensive suite of internal controls in both *in vitro* and *in vivo* studies.

After adding the COL group, the final groups for key ***in vitro*** studies include:

- Blank control
- **COL control**
- PL/COL group
- PUE/COL group
- PL/PUE/COL group
- **Minocycline hydrochloride (Positive Control)**

The related content has been added in **Figure 3A-C** and **Figure 5A-D,** and corresponding descriptions are located on **Page 13 (Lines 351-365)** and **Page 20 (Lines 528-556)**. Besides, to emphasize the key results, we have moved the phagocytosis experiment in original Figure 5A to supplementary materials. Additionally, in order to better clarify the experimental methods and logic, we have adjusted the position and layout of the illustrations in Figure 5. Please see below.

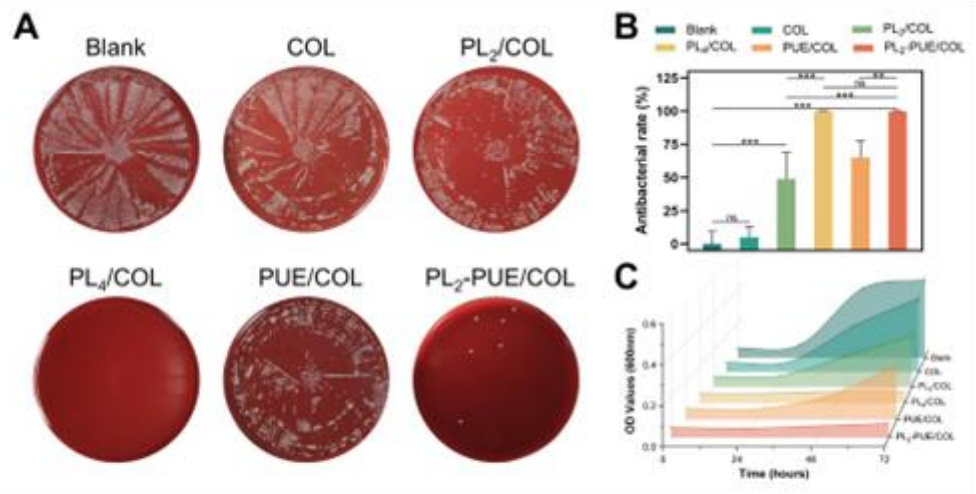

Original Figure 3A-C

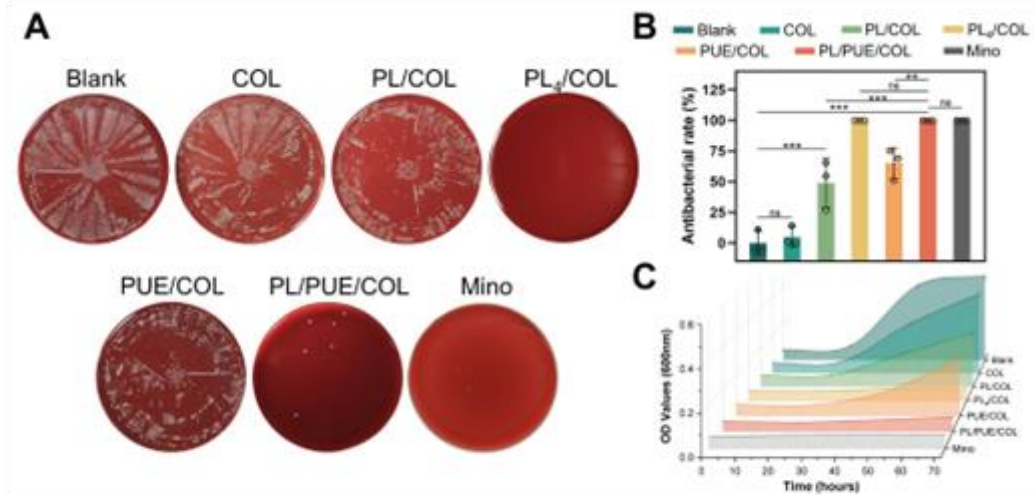

Revised Figure 3A-C

concentration PL (4 mg/mL) exhibited a more significant antibacterial effect compared to the PL/COL group with low concentration of PL (2 mg/mL). PUE/COL hydrogel demonstrated restricted antibacterial effects comparable to PL/COL. Intriguingly, when low-concentration PL was combined with PUE (PL/PUE/COL), a synergistic antibacterial effect was observed, reaching the efficient antibacterial level of PL_4/COL and Mino positive control. The colony count was measured and calculated to reflect the antibacterial efficiency quantitatively (Fig. 3B and Supplementary Fig. 8). The PL_4/COL, PL/PUE/COL and Mino groups achieved an antibacterial rate close to 100%, nearly 2-fold of the PL/COL and PUE/COL groups. To comprehensively assess the effect of supramolecular hydrogel on bacterial growth, we monitored bacterial growth over a 72-hour period (Fig. 3C). In the Blank and COL groups, bacterial growth began rapid proliferation at 24 hours, which slowed down after 48 hours. The PL/COL and PUE/COL groups appeared to slightly inhibit bacterial proliferation, while PL/PUE/COL, PL_4/COL and Mino groups significantly suppressed bacterial proliferation, with no noticeable bacterial growth observed throughout the entire 72-hour period. Live/dead assay was also conducted to visualize the viable (green) and non-viable (red) *P. gingivalis* (Fig. 3D). Blank and COL groups were almost entirely

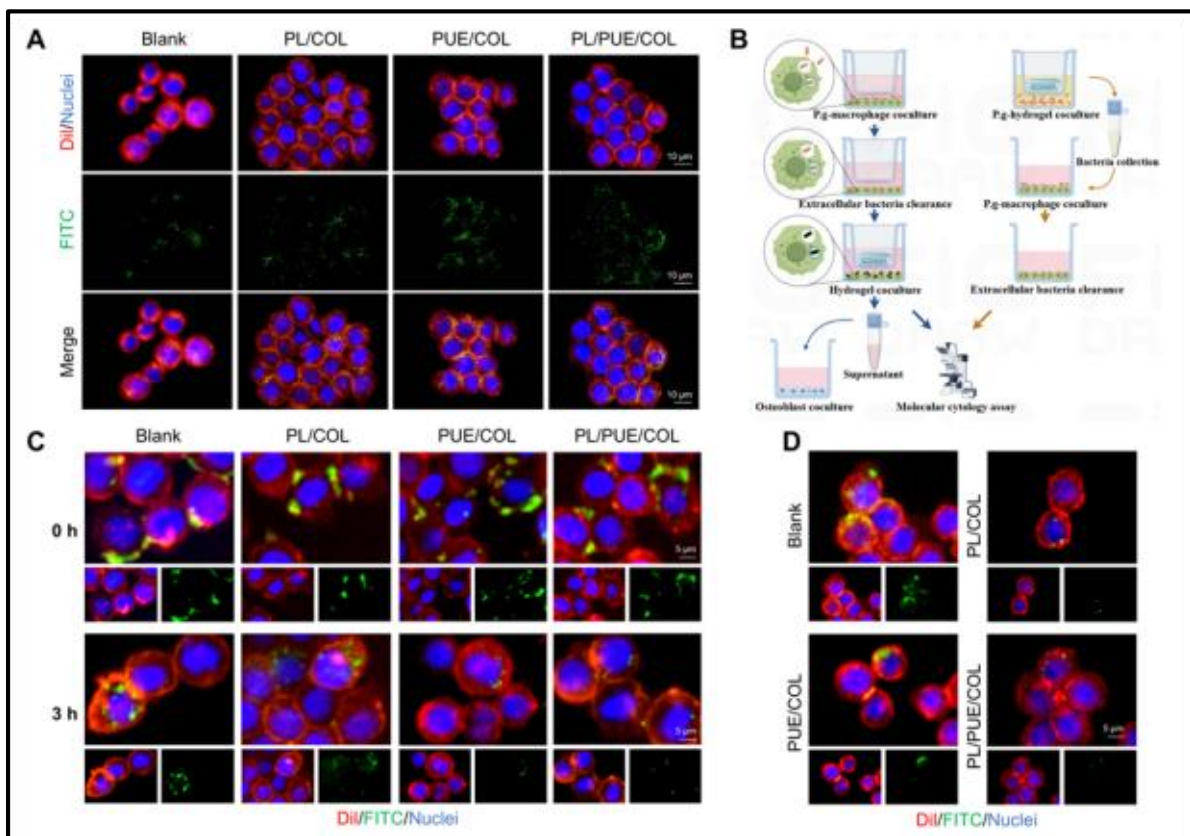

Original Figure 5A-D

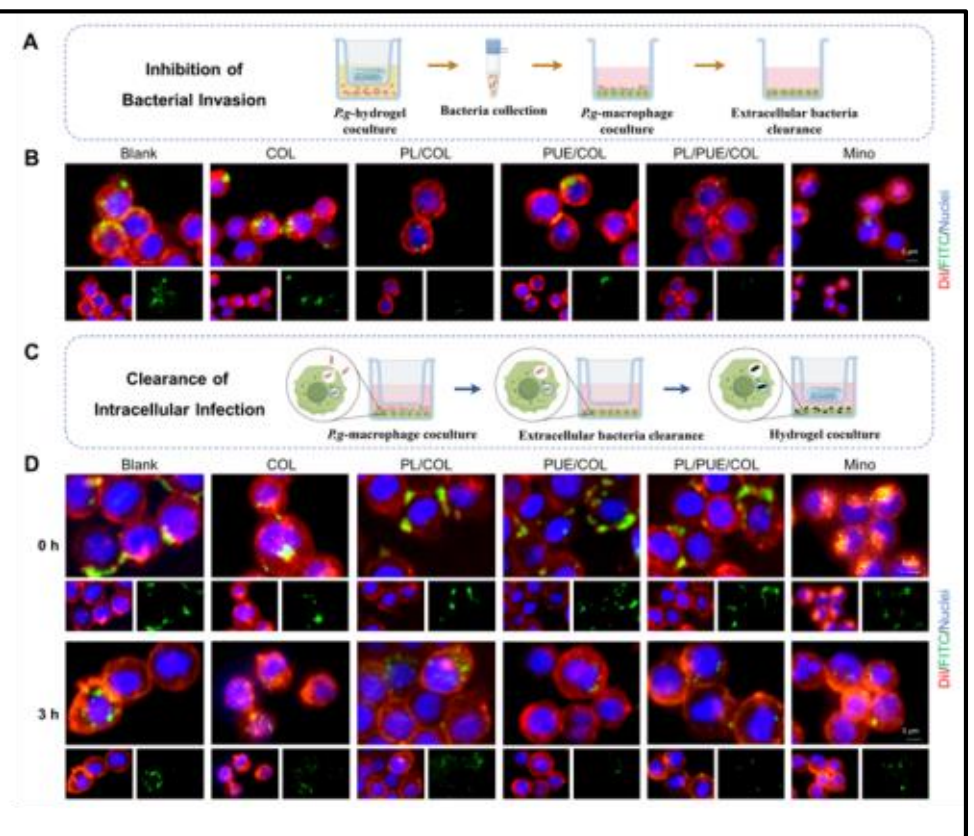

Revised Figure 5A-D

complexes elevated the engulfment ability of macrophage.

However, due to the immune evasion of *P. gingivalis*, robust phagocytosis alone is insufficient. Enhanced intracellular eradication is essential to prevent bacterial persistence. We further evaluated the capacity of the supramolecular hydrogel to both eliminate intracellular bacteria and attenuate bacterial invasion, using Mino as the positive control at the same concentration based on its robust *in vitro* antibacterial performance (Fig. 3A-C). As illustrated in Fig. 5A, to investigate the ability of hydrogels to attenuate bacterial invasion, bacteria were treated with hydrogels before cocultured with macrophages. Compared with the Blank, COL and PUE/COL groups, the PL/COL, PL/PUE/COL and Mino groups significantly reduced bacterial internalization (Fig. 5B). It may be attributed to the PL-induced and antibiotic-induced disruption of the *P. gingivalis* OM, which impairs its intracellular persistence capacity. Furthermore, under the synergistic action of PUE and PL, the disruption of the electron transport chain on IM hindered ATP production, which further reduced bacterial invasive activity (Supplementary Fig. 14).

To investigate the capacity of the supramolecular hydrogel to eliminate intracellular bacteria, as illustrated in Fig. 5C, RAW264.7 cells were infected by *P. gingivalis* before hydrogels treatments. Macrophages were infected with FITC-labeled *P. gingivalis* for 3 hours. After removing extracellular bacteria and staining macrophage membranes with Dil membrane dye, immediate observation confirmed that the initial bacterial internalization was comparable among all groups. Subsequently, the infected macrophages were cocultured with the different hydrogels for another 3 hours. Compared to the Blank group, the other groups all exhibited reduced intracellular bacterial persistence to varying degrees (Fig. 5D). However, despite its superior extracellular bactericidal efficacy, the Mino positive control displayed unsatisfactory intracellular eradication efficiency, which was comparable to the PL/COL group. In contrast, the PUE/COL and PL/PUE/COL groups demonstrated significant clearance, evidenced by sporadic intracellular fluorescence signals. These findings indicated that the PUE-based supramolecular complexes effectively eliminated intracellular *P. gingivalis*, significantly outperforming the clinical gold standard Mino.

The presence of persistent intracellular bacteria induced a sustained pro-

After adding the PL/COL and PUE/COL group to optimize internal control, the final groups for our ***in vivo*** studies include:

- Blank control
- Periodontitis group (Negative Control)
- **PL/COL group**
- **PUE/COL group**
- PL/PUE/COL group
- **Minocycline hydrochloride (Positive Control)**

The related content has been added in **Figure 7 A-E,** and corresponding descriptions are located on **Page 27 (Lines 712-750)**. To ensure clear and focused layout, we have adjusted the arrangement of Figure 7 and moved the Masson staining and immunohistochemical staining results to the supplementary materials. Please see below.

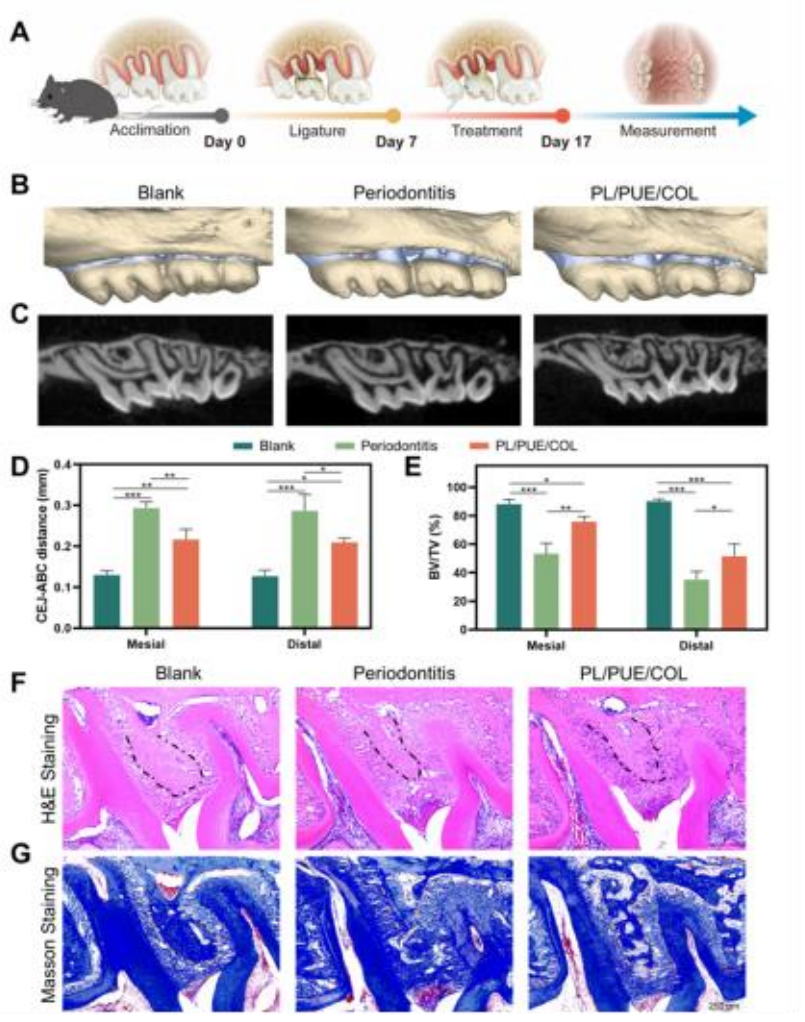

Original Figure 7A-G

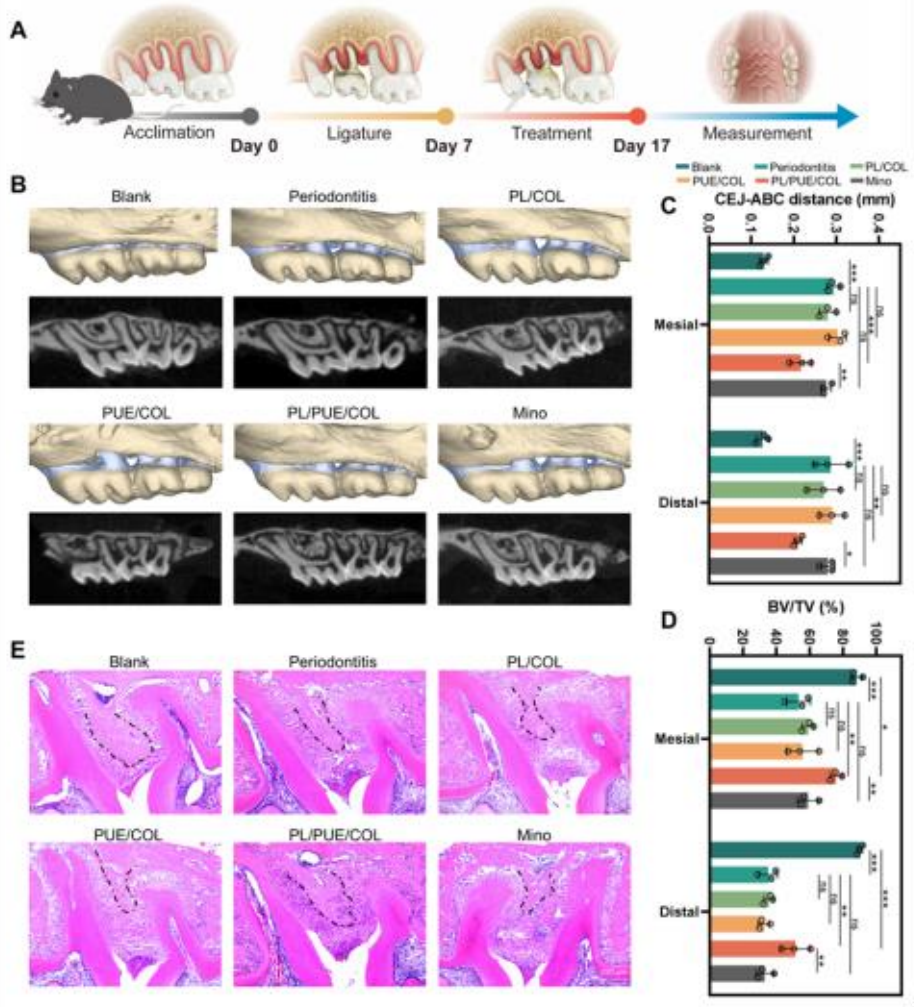

Revised Figure 7A-E

2.7 PL/PUE/COL for *in vivo* periodontitis treatment

To evaluate the *in vivo* therapeutic efficacy of supramolecular hydrogel, a ligature-induced periodontitis model was established in C57BL/6 mice. Building upon the *in vitro* synergy between PL and PUE, we focused on the therapeutic performance of the PL/COL, PUE/COL and PL/PUE/COL supramolecular hydrogel *in vivo*, incorporating the clinical gold standard antibiotic Mino as positive control. Following the process illustrated in Fig. 7A, hydrogels or Mino was locally injected into the periodontal pockets. Micro-computed tomography (Micro-CT) was employed to visualize and quantify the alveolar bone resorption. Compared to the Blank group, the Periodontitis group exhibited significant alveolar bone loss around M2. The distance between the cemento-enamel junction and alveolar bone crest (CEJ-ABC) notably increased, confirming the successful establishment of the periodontitis model. Treatments with the single-component hydrogels (PL/COL and PUE/COL) and the clinical gold standard Mino failed to effectively arrest the bone resorption, exhibiting severe alveolar bone loss comparable to the untreated Periodontitis group. In contrast, mice treated with PL/PUE/COL showed improved alveolar bone recovery, as exhibited in the three-dimensional reconstructed and cross-sectional view (Fig. 7B). Measurements of the CEJ-ABC distance were conducted at both the mesial and distal aspects of M2. While the PL/COL, PUE/COL, and Mino groups maintained large CEJ-ABC distances similar to the Periodontitis group, the PL/PUE/COL treated group consistently showed a reduced distance at these sites, indicating the preservation of alveolar bone height (Fig. 7C). To further characterize bone structure, bone volume fraction (BV/TV) was quantified at both the mesial and distal aspects. The Blank group exhibited the highest BV/TV values, reflecting the most intact alveolar bone structure. The Periodontitis group had the lowest BV/TV values, which indicated a significant reduction in alveolar bone mass following periodontitis. Similarly, the PL/COL, PUE/COL, and Mino groups maintained low BV/TV levels, while PL/PUE/COL group showed an improvement in BV/TV values (Fig. 7D). The quantified values were consistent with the results of three-dimensional reconstruction images, thereby confirming the PL/PUE/COL supramolecular hydrogel had a significant advantage in addressing alveolar bone destruction in periodontitis via its synergistic potent antibacterial activity and immune-metabolic regulation.

Histological evaluation via hematoxylin and eosin (HE) staining revealed that the Periodontitis group exhibited pronounced pathological characteristics, including inflammatory cell infiltration, apical migration of the junctional epithelium, and alveolar ridge resorption. The destructive phenotypes were also observed in the PL/COL, PUE/COL, and Mino treated groups, indicating their limited therapeutic efficacy. In comparison, the PL/PUE/COL group demonstrated effective tissue restoration with reduced inflammation infiltration and the re-establishment of

2. We *also believe that benchmarking against the gold standard therapy is needed to demonstrate an advance.*

Response:

We deeply appreciate this insightful suggestion. To explicitly demonstrate the superiority of our strategy over current clinical therapies, we have added Minocycline hydrochloride, the universally recognized clinical gold standard for periodontitis treatment, as the positive control.

This gold standard group was added to our key experiments in:

- **Antibacterial assays** ***(Figure 3A-C, Line 351 to 365 at Page 13)***
- **Intracellular bacterial eradication assays** ***(Figure 5A-D, Line 528 to 556 at Page 20)***
- ***In vivo* periodontitis model** ***(Figure 7 A-E, Lines 712 to 750 at Page 27)***

The specific details of the changes are already included in the modifications of the first point.

Additional Enhancement

Additionally, we have included Molecular Dynamics (MD) simulations to provide theoretical support for the supramolecular interactions between PL, PUE, and COL, which aligns with our experimental characterizations.

The MD results have been added in **Figure 1D-F** and **Supplementary Figure 2-4.** The related description of results and methodology have been supplied on **Pages 7-8 (Lines 167-203) and Page 31 (Lines 831-854).**

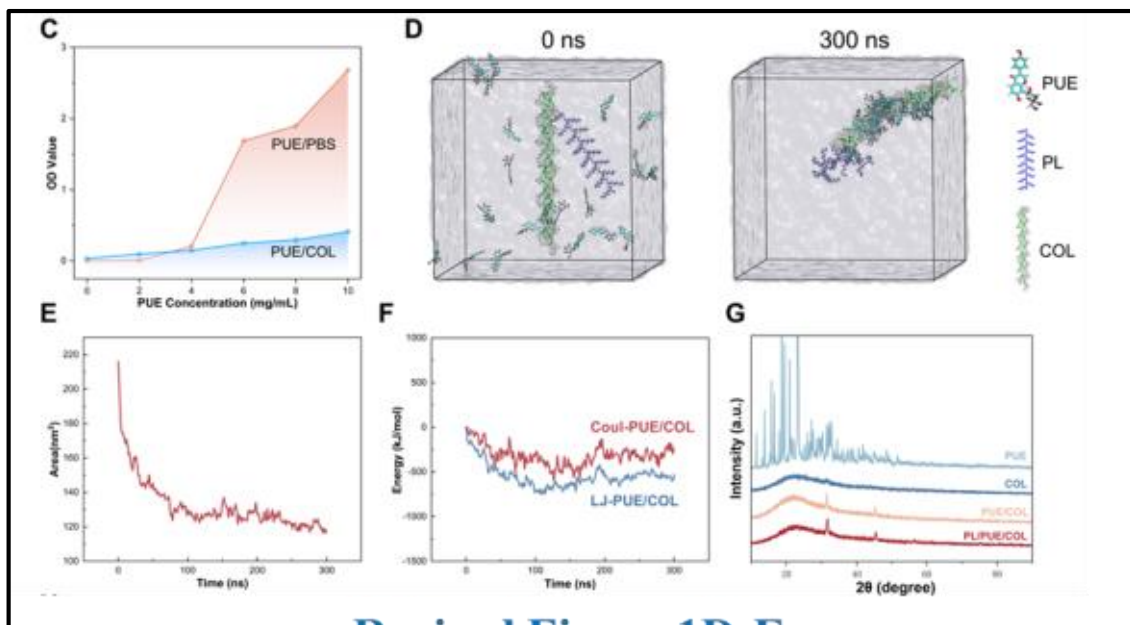

Revised Figure 1D-F

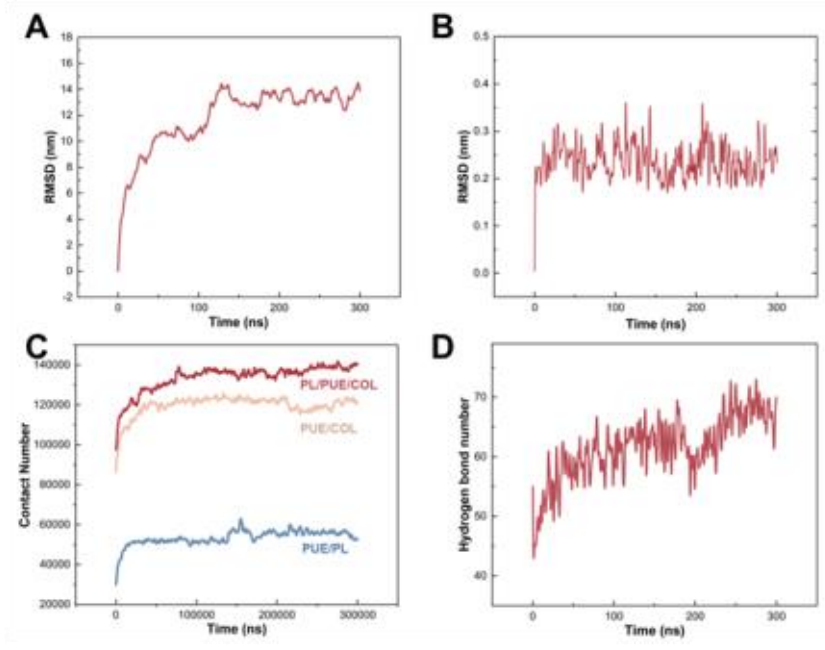

Supplementary Figure 2

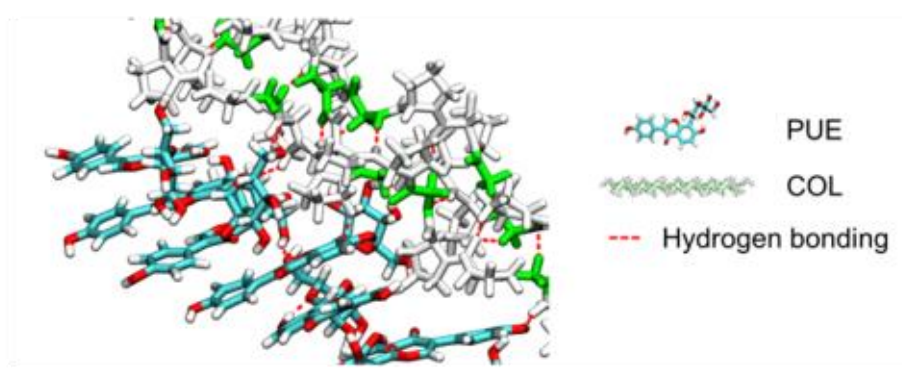

Supplementary Figure 3

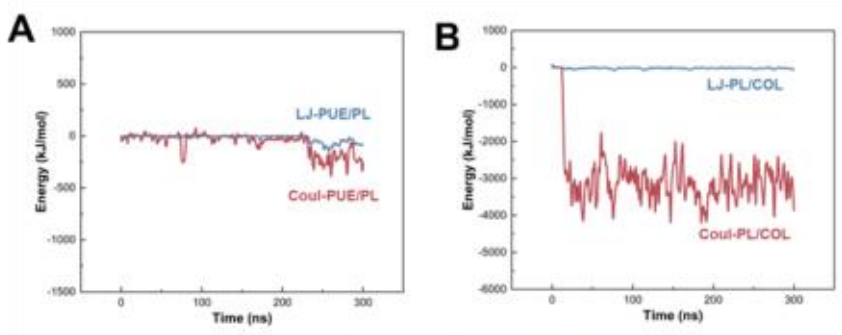

Supplementary Figure 4

supramolecular matrix (Fig. 1C).

To elucidate the underlying molecular mechanisms driving this macroscopic solubilization, we performed molecular dynamics (MD) simulations. As depicted in the simulation snapshots, the initially dispersed PUE, PL, and COL molecules rapidly aggregated and spontaneously reorganized into a highly compact ternary cluster (Fig. 1D). As evidenced by the Root Mean Square Deviation (RMSD) profiles, the entire supramolecular cluster rapidly converged to a stable equilibrium following initial structural fluctuations, indicating the formation of a dynamically stable aggregate (Supplementary Fig. 2A). Importantly, the RMSD of the COL backbone remained consistently low (< 0.3 nm) throughout the assembly process, indicating that the structural integrity of the COL was not compromised (Supplementary Fig. 2B). The structural compaction was quantitatively corroborated by a significant reduction of over 40% in the solvent-accessible surface area (SASA), revealing the burial of hydrophobic isoflavone backbone of PUE to form a dense co-assembly (Fig. 1E). As shown in Supplementary Fig. 2C, the contacts between PUE and COL increased sharply within the first 50 ns and subsequently reached a stable plateau, which indicates a high binding affinity. Following this rapid adsorption, the system underwent continuous spatial adjustments to minimize the total energy, corresponding to the gradual decrease in SASA (Fig. 1E).

Furthermore, quantitative evaluation of Coulomb interactions and Lennard-Jones potentials demonstrated that the interaction energy between PUE and the COL scaffold was predominantly governed by Lennard-Jones potentials (Fig. 1F). Structurally, the proline-rich triple-helical conformation of COL provides abundant hydrophobic regions that effectively accommodate the hydrophobic isoflavone core of PUE[43,45]. The hydrophobic packing was further stabilized by a dense intermolecular hydrogen-bonding network between the abundant amide/hydroxyl groups of COL and the phenolic hydroxyls of PUE (Supplementary Fig. 2D). Specific hydrogen bond anchoring sites were clearly identified in the simulated structural details (Supplementary Fig. 3). Interestingly, the interaction energy between PL and PUE was nearly negligible, while the interaction between the PL and COL was overwhelmingly driven by Coulomb interactions (Supplementary Fig. 4). This strong attraction is attributed to the high-affinity anchoring of PL's abundant amino groups to the anionic domains of COL. Significantly, the assembly mode corroborates the successful supramolecular functional integration, which allows the system to effectively stabilize the amorphous PUE molecules without PUE interfering with or masking the intrinsic cationic antibacterial sites of PL.

The assembly mechanisms predicted by MD simulations were further validated through experimental characterizations. X-ray diffraction (XRD) analysis showed that pure PUE exhibited sharp diffraction peaks characteristic of a crystalline lattice.

3.1.3 Molecular dynamics simulation

The structure of the PUE molecule was optimized at B3LYP-D3(BJ)/6-31G(d,p) level using Gaussian 16 A.03 software, and vibration analyses were carried out to ensure that the structures have no imaginary frequency[70,71]. The RESP charge was calculated using Multiwfn 3.8(dev) software, and the GAFF2 force field parameters were obtained using acpype code[72]. The initial coordinates of COL model were derived from the Protein Data Bank (PDB ID: 1K6F). A 15-mer PL was constructed with fully protonated side chains (-NH3+) to reflect its physiological cationic state. Both the COL and PL models were terminally capped at their N- and C-termini to neutralize unnatural terminal charges. The Amber ff14SB force field parameters were used for the protein and the TIP3P water model was used here. Molecular dynamics simulations were performed using GROMACS 2021.7 software. Long-range electrostatic interactions were treated with the particle mesh Ewald (PME) method with 1.2 nm as the Coulomb cutoff, and the van der Waals (vdW) interactions were treated with the force-switching method, where the forces smoothly decayed to zero between 0.9 and 1.2 nm to reduce the cutoff noise[73]. The LINCS algorithm was used to apply the bond constraint related to hydrogen. All the simulation systems were energy-minimized for 5000 steps using the steepest descent algorithm, and then underwent 1 ns of NPT pre-equilibration run using leap-frog MD integrator and followed by a 300 ns unrestrained production run with a time step of 1 fs. During all the simulations, temperature was maintained at 298.15 K using the velocity rescaling (V-rescale) thermostat (with τ_t = 1.0 ps), and pressure was kept at 1 bar by C-rescale isotropic barostat (with τ_p = 2.0 ps)[74]. The three-dimensional periodic boundary condition (PBC) was used. Results were visualized using VMD 1.9.3 software.

Point-by-Point Response to Reviewers

Reviewer #1：

The paper entitled "A Supramolecular Strategy Orchestrates Dual-Compartment Pathogen Clearance and Immuno-Metabolic Homeostasis to Reverse Periodontitis" designed an injectable supramolecular hydrogel for treating intracellular infections and restoring tissue homeostasis in periodontitis. This paper provides innovative approaches to enhance intracellular bacterial clearance and host immune homeostasis. I have concerns about the followed specific analyses before publication, and a minor revision is needed:

Response to the general comment:

We sincerely thank the reviewer for recognizing the innovation of our work and for the valuable suggestions for the manuscript revision. We have carefully considered all the specific concerns raised by the reviewer, and we will respond to and address each point in detail one by one in the following sections.

1. Although FTIR and DSC provide some clues regarding intermolecular interactions, these characterizations are too macroscopic. It is recommended to supplement NMR spectroscopy to observe the chemical shift changes of specific functional groups on PUE, thereby confirming its specific non-covalent binding with COL.

Response:

We sincerely thank the reviewer for the professional and constructive suggestion. We completely agree that NMR spectroscopy provides essential molecular-level evidence to confirm the specific non-covalent binding within the supramolecular system.

In response to this comment, we have supplemented ^1H NMR spectroscopy characterization. We tested and analyzed the ^1H NMR spectra of pure PUE, pure COL and the PUE/COL supramolecular system. By analyzing the locally enlarged spectra (δ 9.0-10.5 ppm), we clearly observed that the characteristic sharp peak representing PUE's phenolic hydroxyl protons at approximately δ 9.6 ppm was suppressed in the PUE/COL group. This significant signal attenuation provides direct, molecular-level evidence of intermolecular hydrogen bonding between the phenolic

hydroxyl groups of PUE and the COL matrix. The corresponding details can be found in the revised manuscript and supplementary information, as also shown below:

Page 8 in the revised manuscript: "To confirm the specific non-covalent interactions within the supramolecular system, 1H nuclear magnetic resonance (NMR) spectroscopy was performed (Supplementary Fig. 6a). In the locally enlarged spectra (δ 9.0-10.5 ppm), the characteristic sharp peak of PUE's phenolic hydroxyl protons at approximately δ 9.6 ppm was suppressed in the PUE/COL co-assembly system (Supplementary Fig. 6b). This signal attenuation indicates the interactions between the phenolic hydroxyl groups of PUE and the COL matrix."

Page 33 in the revised manuscript: "^{1}H NMR spectra were recorded using a Bruker AVANCE II spectrometer (Bruker, 400 MHz, Germany) to investigate the intermolecular interactions. The freeze-dried samples were completely dissolved in deuterated DMSO. The chemical shifts were reported in parts per million (ppm)."

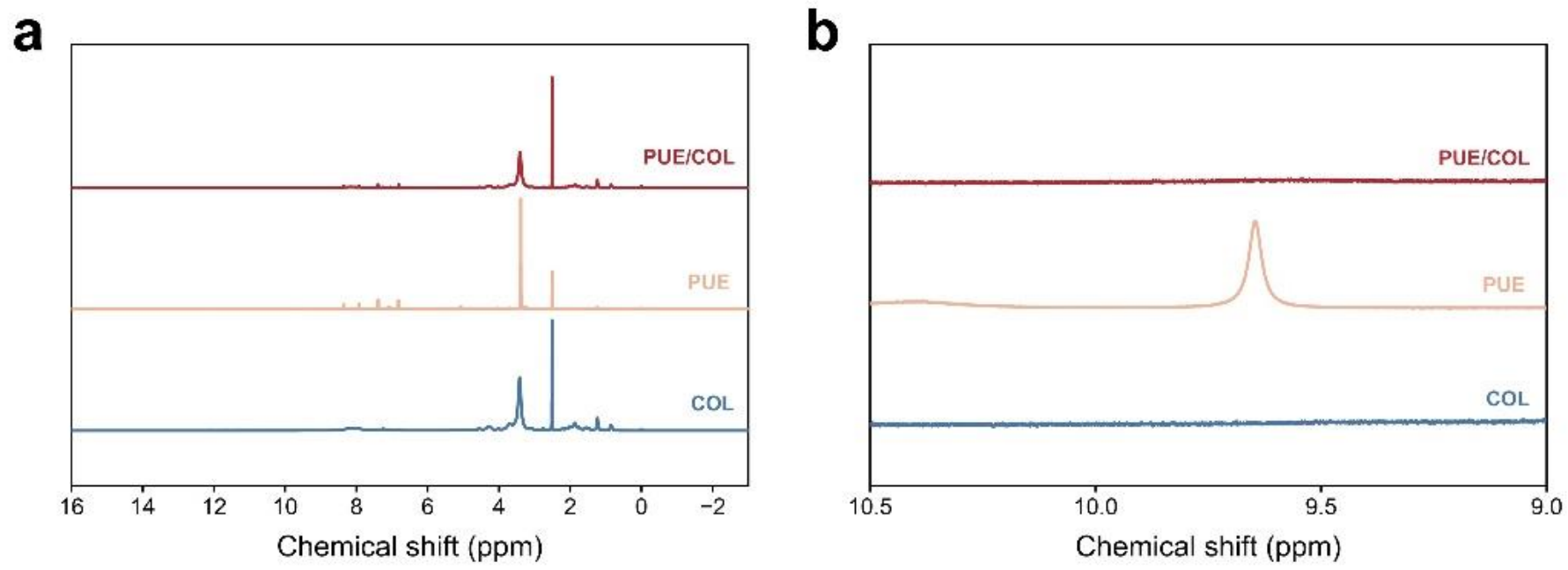

Supplementary Fig. 6. ^{1}H NMR (a) full spectra and (b) corresponding locally enlarged spectra (δ 9.0-10.5 ppm) of COL, PUE, and PUE/COL. COL recombinant human type I collagen, PUE puerarin, NMR nuclear magnetic resonance. Source data are provided as a Source Data file.

2. The application of this supramolecular complex is in the oral environment of periodontitis. Can these three components form and maintain a stable supramolecular structure in a complex physiological fluid environment? Will PUE precipitate again? It is suggested to supplement assembly experiments in physiological fluids to verify whether PUE dissociates.

Response:

We appreciate the reviewer's suggestion regarding the stability of the supramolecular complex in physiological environments.

To address this concern, we further investigated the assembly and stability of the system using artificial saliva (AS) to simulate the complex oral environment. As shown in the newly added **Supplementary Fig. 2**, the addition of PUE into pure AS led to obvious macroscopic turbidity and precipitation. In contrast, the PUE/COL supramolecular system prepared in AS maintained a stable condition without visible sedimentation, though it showed a slight opalescence.

These results confirm that the supramolecular co-assembly can be achieved and maintained in physiological fluids, thereby preventing the re-precipitation of PUE in the oral environment. The corresponding details have been added to the revised manuscript and supplementary information, as also shown below:

Page 7 in the revised manuscript: "Considering the application of the supramolecular complex in the oral environment, we further investigated its assembly stability in artificial saliva (AS). While the direct addition of PUE into AS led to macroscopic turbidity and precipitation, the PUE/COL/AS supramolecular system maintained a stable condition without visible sedimentation, exhibiting a slight opalescence (Supplementary Fig. 2). It confirms that the supramolecular co-assembly can be effectively achieved and maintained in the complex environment of saliva."

Page 33 in the revised manuscript: "To validate the solubilization capacity and supramolecular co-assembly behavior within a physiological oral microenvironment, artificial saliva (AS; Solarbio, China) was utilized as the solvent. COL (100 mg/mL) was first dissolved in AS, followed by the addition of PUE (10 mg/mL), and the co-assembly process was conducted as described above. The control group was prepared by adding PUE (10 mg/mL) directly into pure AS. The mixtures were thoroughly stirred, and macroscopic photographs were captured immediately and after 5 minutes of standing, to evaluate the solubility and sedimentation."

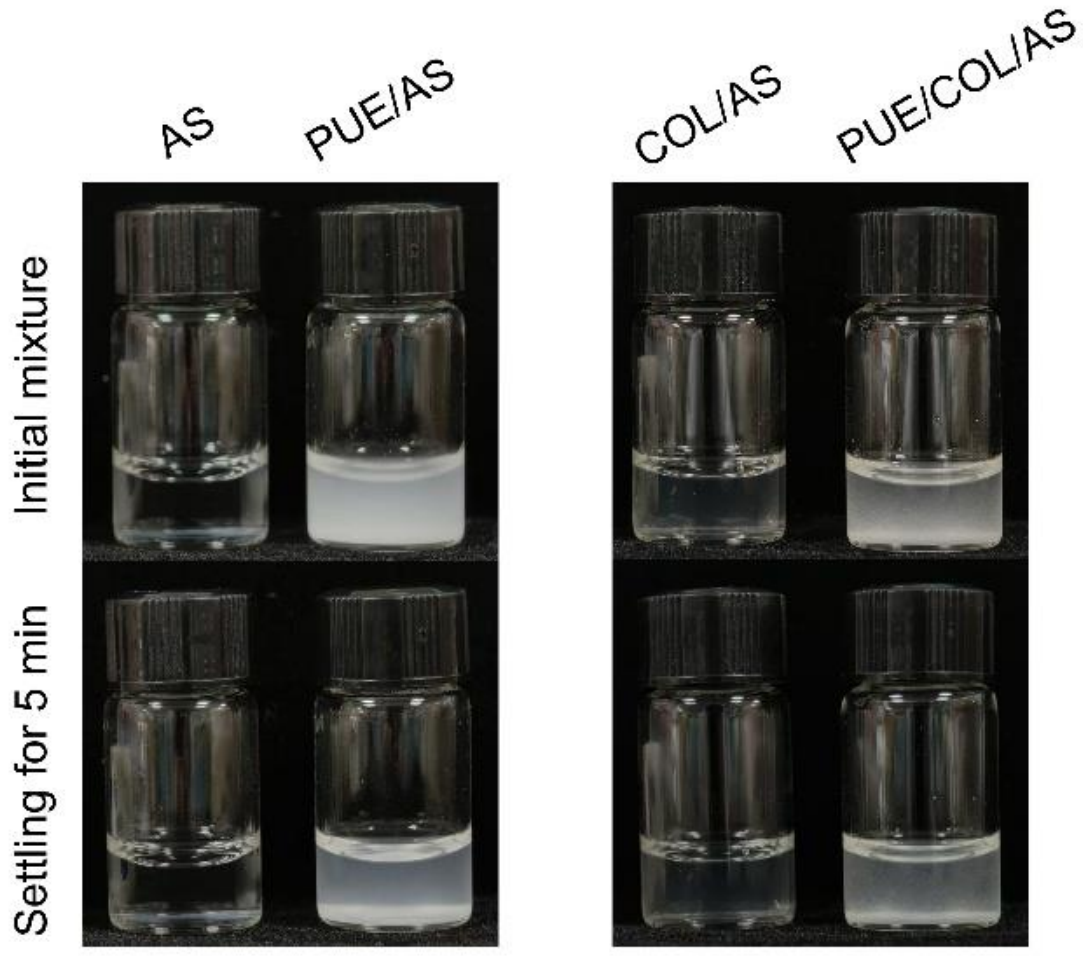

Supplementary Fig. 2. Optical images of the solubility and sedimentation of 10 mg/mL PUE in pure AS and COL-containing AS solution. COL recombinant human type I collagen, PUE puerarin, AS artificial saliva.

3. Expressing the antimicrobial effect as "Antibacterial rate (%)" in Figure 3B is non-standard. In microbiology, a 90% antibacterial rate merely represents a 1-log reduction in CFU, which is considered ineffective in the field of antibacterial materials. A true antibacterial effect requires at least a 3-log (99.9%) reduction. Please change the y-axis of all relevant figures to CFU counts or Log-transformed values.

Response:

We appreciate your rigorous and professional comment regarding the standards for evaluating antibacterial efficacy. We fully agree that CFU counts provide a more standardized and accurate representation of true bactericidal effects.

To address your concern, we have replaced the "Antibacterial rate (%)" graph in the main text with the CFU quantification graph, which is now presented as **Figure 3b**. The percentage-based antibacterial rate graph has been relocated to **Supplementary Fig. 11**. As demonstrated in the updated **Figure 3b**, the PL_4/COL, PL/PUE/COL, and Mino groups resulted in an approximate 1000-fold decrease in CFU counts. This reduction effectively confirms the antibacterial capacity of the supramolecular hydrogel.

The corresponding details have been added to the revised manuscript and supplementary

information, as also shown below:

Page 15 in the revised manuscript: "The colony-forming unit (CFU) counts were quantified to evaluate the antibacterial efficiency (Fig. 3b). While the PL/COL and PUE/COL treatments only partially decreased the viable bacterial counts compared to the Blank and COL groups, the PL_4/COL, PL/PUE/COL, and Mino groups effectively eradicated the bacteria, reducing the CFU counts by approximately 1000-fold. The corresponding calculated antibacterial rates are provided in Supplementary Fig. 11, confirming that the PL_4/COL, PL/PUE/COL and Mino groups achieved an antibacterial rate close to 100%, nearly 2-fold higher than those of the PL/COL and PUE/COL groups."

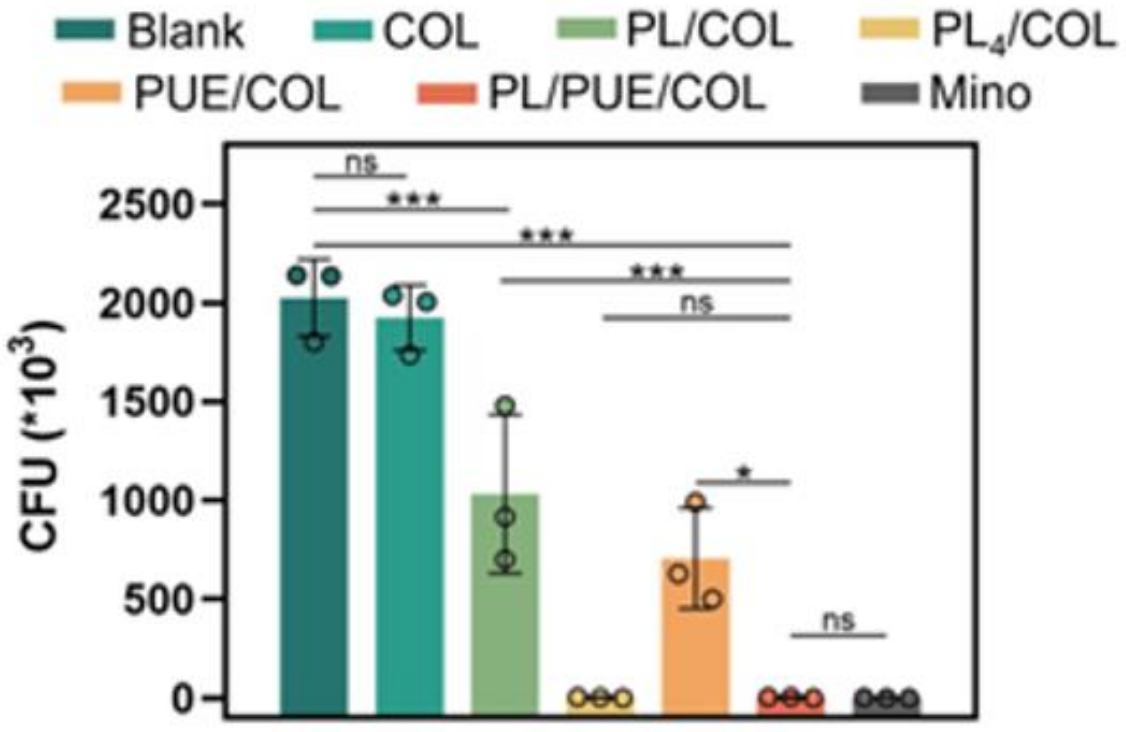

Fig. 3 Evaluation of antibacterial capability and biocompatibility. (b) Quantification of *P. gingivalis* colonies (n=3).

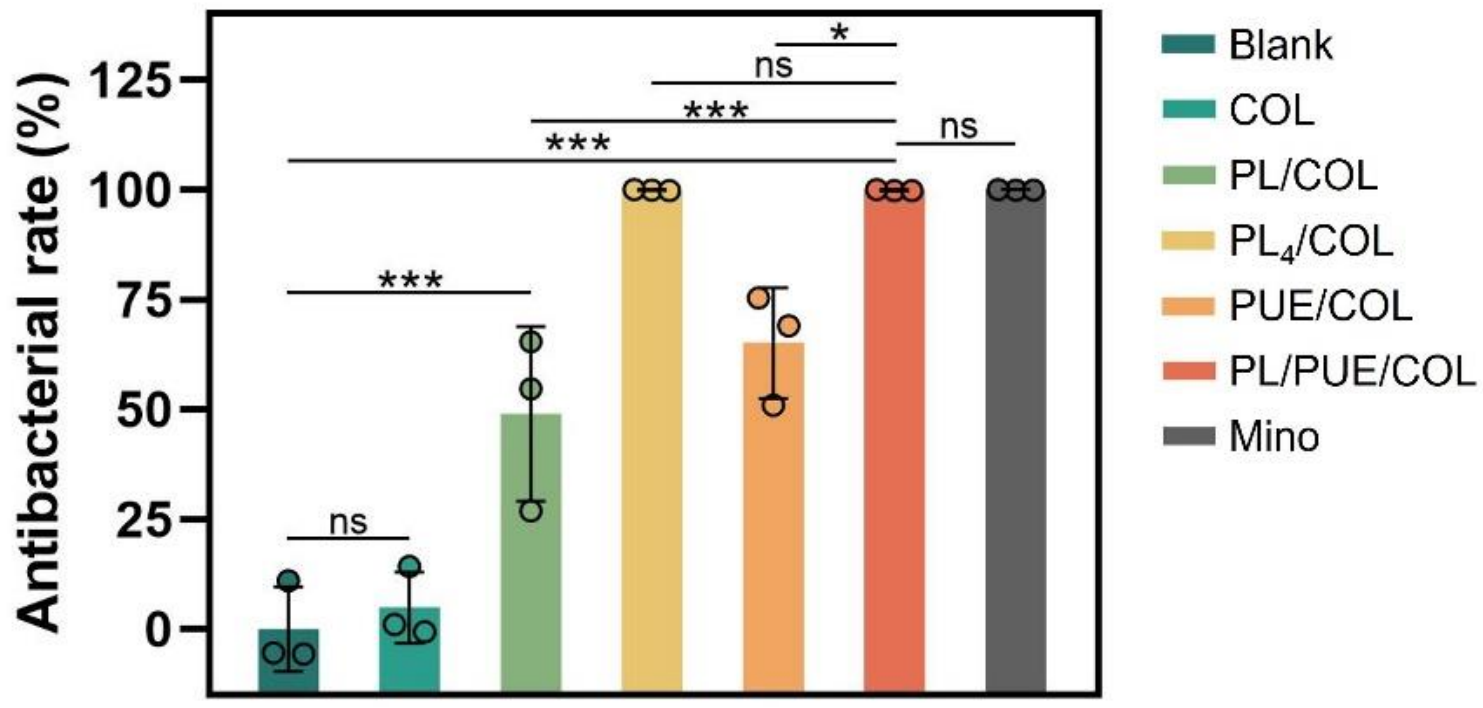

Supplementary Fig. 11. Quantitative analysis of corresponding antibacterial rates in Fig. 3a (n=3). All error bars represent mean ± SD. P values were calculated by one-way ANOVA followed by Tukey's post hoc test (*P < 0.05; **P < 0.01; ***P < 0.001; ns, not significant). Source data are provided as a Source Data file.

4. Periodontitis is caused by highly complex polymicrobial biofilms rather than single planktonic bacteria. The authors only tested single P. gingivalis and F. nucleatum models. It is recommended that the authors establish an in vitro dual-species or multi-species infection model to verify the clearance capability of this supramolecular system in a complex infection.

Response:

We sincerely appreciate this constructive suggestion. We completely agree that periodontitis is driven by complex polymicrobial communities, and evaluating our supramolecular system in a mixed-species model provides a more rigorous validation of its therapeutic potential.

For this purpose, we established an *in vitro* dual-species infection model comprising *P. gingivalis* and *F. nucleatum*, following a previously reported methodology (Tavares et al., *J Periodontal Implant Sci*, 2018). As demonstrated in the newly added **Supplementary Fig. 14**, while the Blank, COL, PL/COL, and PUE/COL groups exhibited limited inhibition against the polymicrobial challenge, the PL/PUE/COL hydrogel effectively suppressed the growth of the mixed bacterial population throughout a 72-hour observation period. The inhibitory efficacy of the PL/PUE/COL hydrogel was comparable to both the PL_4/COL group and the clinical standard minocycline group. It confirms that the synergistic antibacterial capacity of the supramolecular system remains effective even within a complex dual-species infection microenvironment.

The corresponding details have been added to the revised manuscript and supplementary information, as also shown below:

Page 16 in the revised manuscript: "It confirms the bactericidal activity of the PL/PUE/COL supramolecular complex against *F. nucleatum.* Since periodontitis microenvironment is typically characterized by complex polymicrobial communities rather than a single pathogen, we further evaluated the antibacterial ability against an *in vitro* dual-species infection model comprising *P. gingivalis* and *F. nucleatum*. As shown in Supplementary Fig. 14, the Blank and COL groups exhibited rapid and continuous bacterial proliferation. The PL/COL and PUE/COL hydrogels provided only limited inhibition against the polymicrobial challenge. In contrast, the PL/PUE/COL hydrogel effectively suppressed the growth of the mixed bacterial population throughout the 72-hour period. Its efficacy was close to both the high-dose PL_4/COL and the clinical standard Mino group. It indicates the synergistic antibacterial capacity remains satisfactory even in the complex

dual-species infection microenvironment."

Page 36 in the revised manuscript: "A dual-species infection model was established as previously reported[83]. *P. gingivalis* (ATCC 33277) and *F. nucleatum* (ATCC 25586) suspensions (1 × 10^6 CFU/mL) were mixed at a 1:1 volume ratio to acquire dual-species bacterial suspension. Subsequently, 300 μL of the suspension was further incubated with 100 μL of sterilized hydrogels in a 48-well plate at 37°C under anaerobic conditions. The OD values at 600 nm were monitored every 24 hours over a 72-hour period."

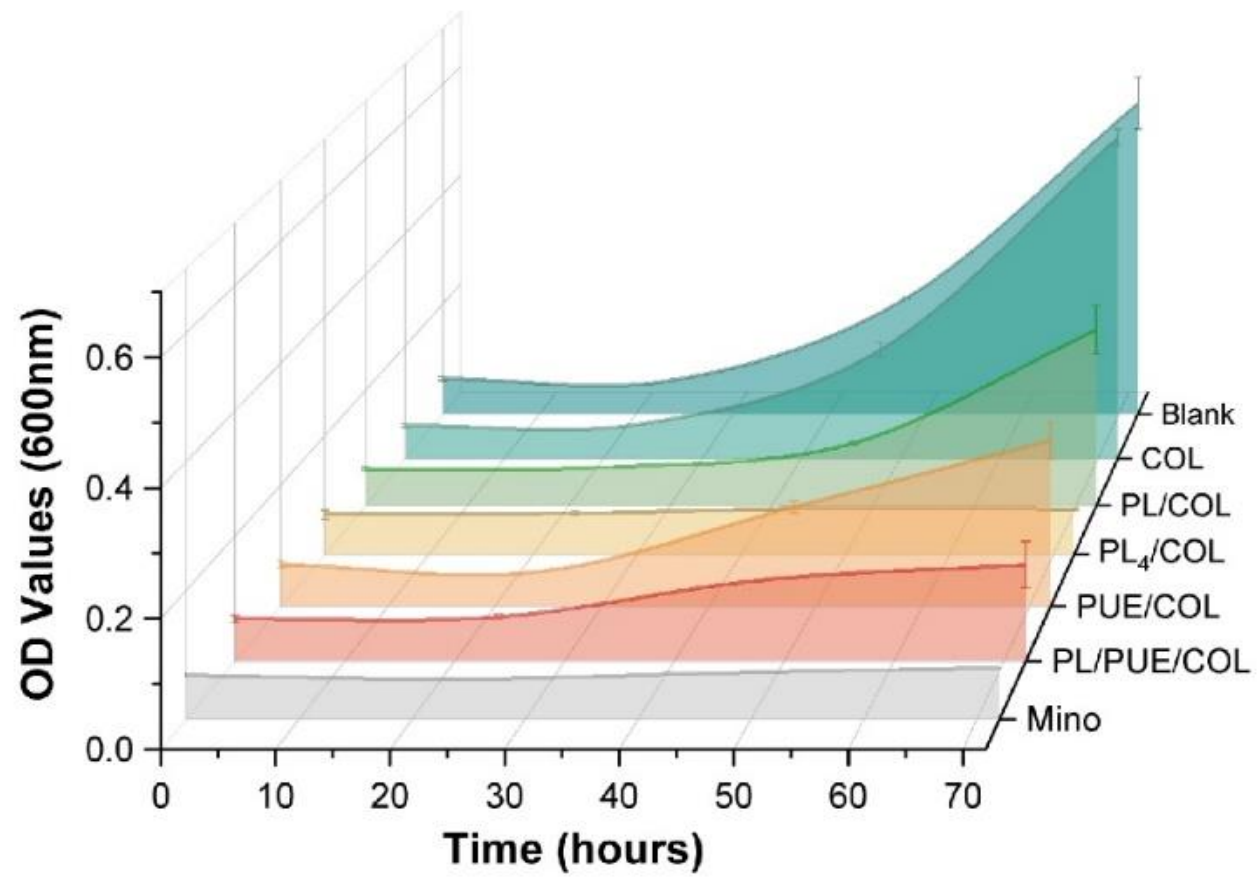

Supplementary Fig. 14. Growth curves of mixed infection model of *F. nucleatum* and *P. gingivalis* cultured with or without different hydrogels over a 72-hour period (n=3). All error bars represent mean ± SD. Source data are provided as a Source Data file.

5. Although P. gingivalis is a key pathogen (Gram-negative), periodontal infections are often accompanied by Gram-positive bacteria. The mechanism of PL primarily targets the LPS-rich outer membrane. Since Gram-positive bacteria lack an outer membrane but have a very thick peptidoglycan layer, does this supramolecular hydrogel still possess synergistic bactericidal effects against them?

Response:

We sincerely appreciate your insightful comment regarding the differences in bacterial structures and their corresponding therapeutic challenges.

In this study, our material design primarily focused on Gram-negative pathogens, such as *P. gingivalis* and *F. nucleatum*. These species are key pathogens in periodontitis and generally exhibit

a greater antimicrobial challenge than Gram-positive bacteria. This difficulty stems from their complex outer membrane (OM), which is an asymmetric lipid bilayer containing lipopolysaccharides (LPS) in its outer leaflet (Nikaido H, *Microbiol Mol Biol Rev*, 2003). The LPS hydrocarbon domain exhibits low fluidity and strong lateral interactions, functioning as a highly restrictive permeability barrier. Furthermore, the OM and the inner membrane (IM) possess orthogonal sieving properties, which limit the intracellular accumulation of antibacterial agents coupled with active efflux systems (Silver LL, *Clin Microbiol Rev*, 2011). The synergistic PL/PUE supramolecular mechanism provides a potential strategy to breach this robust OM barrier and subsequently disrupt the inner membrane.

Regarding Gram-positive bacteria, although they possess a thick peptidoglycan layer, the absence of an outer lipid membrane generally makes their cell envelope more permeable and susceptible to a broader spectrum of antimicrobial agents (Silver LL, *Clin Microbiol Rev*, 2011). As demonstrated in our previous studies, low concentrations of cationic PL alone can effectively achieve reliable bactericidal efficacy against Gram-positive bacteria while maintaining favorable biocompatibility (Zheng T, et al., *J Mater Chem B*, 2026. doi:10.1039/d6tb00100a).

Therefore, while the synergistic design of this supramolecular hydrogel was optimized to overcome the specific challenges caused by the Gram-negative OM, the system inherently retains effective antimicrobial activity against Gram-positive bacteria, primarily driven by the cationic properties of PL. We hope this clarifies our targeted design rationale.

6. In validating the anti-inflammatory properties, LPS was used to stimulate RAW264.7 cells to evaluate macrophage immune behavior. However, in subsequent experiments, this was switched to live P. gingivalis infection. There is a lack of necessary logical transition; please explain the specific rationale for switching from LPS to P. gingivalis.

Response:

We apologize for the abrupt logical transition in the original manuscript and agree that the switching between these two models requires explicit clarification.

The experimental design reflects the distinct pathogenic mechanisms and therapeutic targets in the periodontitis microenvironment. Initially, LPS was utilized to establish an *in vitro* model of the

extracellular inflammatory environment without the complex situation introduced by active bacterial invasion.

However, alleviating extracellular inflammation alone is insufficient for effective periodontitis therapy. As a main pathogen, *P. gingivalis* actively invades macrophages and evades immune clearance, leading to persistent intracellular infection and immuno-metabolic dysregulation. Therefore, to further evaluate the elimination of intracellular bacteria and the restoration of cellular homeostasis after our supramolecular hydrogel treatment, the *P. gingivalis* infection model was established.

To clarify this logical progression for readers, we have rewritten the corresponding section in the revised manuscript. The corresponding details have been modified in the revised manuscript, as also shown below:

Page 22 in the revised manuscript: "While alleviating extracellular LPS-induced inflammation is crucial, periodontitis pathogens such as *P. gingivalis* can actively invade host cells to evade immune clearance, disrupting local immune and metabolic responses. As the first line of innate defense, macrophages play a pivotal role in eradicating these pathogens. Therefore, beyond general immunomodulation, enhancing the capacity of macrophages to clear intracellular bacteria is essential to overcome immune evasion and restore the osteo-immune microenvironment."

7. It is recommended to supplement experiments to demonstrate the restoration of lysosomal activity.

Response:

We sincerely appreciate your valuable suggestion. We agree that demonstrating the restoration of lysosomal activity is crucial for validating our mechanistic hypothesis.

Nevertheless, our existing experimental results have provided evidence regarding the improvement of the lysosomal status. To address your concern and make these indications clearer, we have optimized the data presentation and corresponding expression in the revised manuscript.

1) **Comparison of lysosome-associated gene expression (Fig.6e-6f):** We have highlighted the direct comparison of the transcription levels of LAMP1 and CTSD among the Blank, *P. gingivalis* (*P.g*), and *P.g*-PL/PUE/COL groups. This verification at the mRNA level further substantiates the hypothesis regarding the restoration of lysosomal activity, which

was initially proposed based on our RNA sequencing results.

2) **Spatial fluorescence intensity profile analysis (Fig.6g):** To provide a more intuitive evaluation, we added a spatial fluorescence intensity profile analysis for the colocalization of FITC-labeled bacteria and LysoTracker Red. The synchronized spatial overlap suggests that the internalized bacteria are located within acidic environments, which provides evidence for the recovery of phagolysosomal acidification.

We recognize that these are indirect evaluations, and a more in-depth investigation involving direct lysosomal functional assays will be a focus of our future studies. We have carefully revised the relevant descriptions in the text to ensure our conclusions are objective and strictly grounded in the data. The corresponding details have been updated in the revised manuscript, as also shown below:

Page 26-27 in the revised manuscript: "In this view, we hypothesized that the PL/PUE/COL hydrogel could revive lysosomal function to eradicate intracellular *P. gingivalis* and influence metabolic behaviors. To validate this hypothesis, we further detected the mRNA expression levels of lysosomal associated membrane protein 1 (LAMP1) and cathepsin D (CTSD) via real-time quantitative polymerase chain reaction (PCR), which reflected lysosomal homeostasis and function. As a marker of lysosomal structural integrity, LAMP1 exhibited downregulation following *P. gingivalis* infection, and it was significantly restored after treatment with the PL/PUE/COL supramolecular hydrogels (Fig. 6e). Similarly, CTSD, an important lysosomal enzyme involved in bacterial degradation, was downregulated following *P. gingivalis* infection, and this reduction was significantly restored after PL/PUE/COL treatment (Fig. 6f). These results indicated that the PL/PUE/COL supramolecular hydrogel not only restored the V-ATPase complex, but also promoted the homeostasis and enzymatic capacity of lysosome, enhancing the capacity for effective bacterial clearance.

Subsequently, we investigated whether it translated to effective phagolysosomal fusion at the cellular level. We visualized the spatial relationship between intracellular FITC-labeled *P. gingivalis* and lysosomes stained with LysoTracker Red (Fig. 6g). To validate the colocalization, spatial fluorescence intensity profiles were plotted along the paths indicated by the white dashed arrows. In the untreated *P. gingivalis* infection group (*P.g* group) and the PL/COL group, the fluorescence peaks of bacteria (green) and lysosomes (red) were spatially mismatched. Since LysoTracker

selectively accumulates in acidic compartments, this separation indicated that *P. gingivalis* evaded phagolysosome fusion or impaired the acidification process. In contrast, after treatment with PUE/COL and PL/PUE/COL, the fluorescence intensity profiles of both channels exhibited a highly synchronized spatial overlap, suggesting a restoration of phagolysosome structure and function. Crucially, since LysoTracker selectively accumulates in highly acidic compartments, its spatial colocalization with the bacteria indicated restored lysosomal acidification. Combined with the molecular upregulation of essential lysosomal proteases (CTSD) and structural membrane proteins (LAMP1), these findings confirm that the PUE-based supramolecular hydrogel revives the lysosomal bioactivity."

Page 26-27 in the revised manuscript: "For colocalization analysis, spatial fluorescence intensity profiles were generated using Fiji ImageJ software along the paths indicated by the white dashed arrows to map the spatial distribution of FITC and LysoTracker signals."

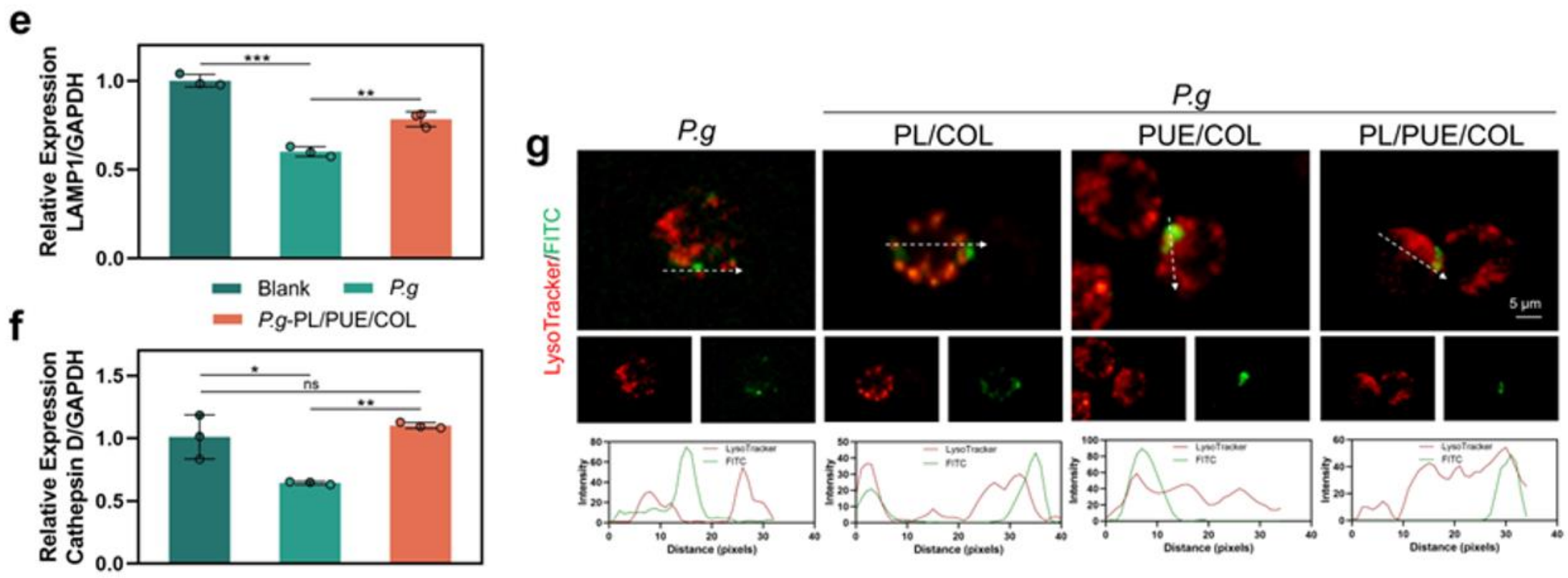

Fig. 6 Mechanism of PL/PUE/COL-mediated immuno-metabolic regulation. Relative mRNA expression of (e) LAMP1 and (f) CTSD in infected RAW264.7 cells with different treatments (n=3). (g) Co-localization images of lysosomes and bacteria.

8. Typically, macrophages eradicate engulfed bacteria by producing large amounts of ROS. As a strong antioxidant, while PUE clears intracellular ROS, will it unavoidably weaken the macrophages' defense of using ROS for bactericidal activity? The authors need to provide a more discussion on this complex redox balance.

Response:

We sincerely appreciate this insightful comment. We have expanded the discussion on the balance between ROS scavenging and bactericidal activity in the revised manuscript. To further

verify the free radical scavenging capacity of our supramolecular complexes, we have included the DPPH assay results in the revised manuscript.

While ROS is a traditional defense mechanism, *P. gingivalis* is known for its ability to evade oxidative killing (Henry LG, et al., *Future Microbiol*, 2012). Our findings suggest that the excessive ROS induced by *P. gingivalis* infection interferes with lysosomal activity, which contributes to bacterial intracellular survival. By scavenging this pathological ROS, PUE helps alleviate oxidative stress and supports the restoration of lysosomal homeostasis and function. Therefore, in this specific infection microenvironment, PUE facilitates a transition from an oxidative burst to more efficient lysosomal degradation for *P. gingivalis* clearance, rather than weakening the overall host defense.

Additionally, to further support these intracellular findings, we have supplemented the DPPH assay to verify the *in vitro* free radical scavenging capacity of the supramolecular system (**Supplementary Fig. 9**). This result provides a functional basis for its intracellular antioxidant performance. We have incorporated the DPPH assay results and the corresponding discussion into the revised manuscript and supplementary information, as also shown below:

Page 27 in the revised manuscript: "Crucially, the supramolecular co-assembly did not compromise the intrinsic bioactivity of PUE. The antioxidant capacity was assessed via the 2,2-diphenyl-1-picrylhydrazyl (DPPH) radical scavenging assay (Supplementary Fig. 9). The radical clearance rate of the PUE/COL groups increased in a concentration-dependent trend, even approaching nearly 100% at the PUE concentrations of 8 and 10 mg/mL. Furthermore, the PL/PUE_{10}/COL supramolecular complex also exhibited an excellent scavenging efficacy comparable to that of PUE_{10}/COL, significantly beyond the PL/COL group and COL group. It demonstrates that PUE retains its antioxidant capability when integrated into the supramolecular complex. More importantly, by overcoming the inherent aqueous insolubility which practically impedes the use of free PUE in physiological fluids, the supramolecular strategy provides an effective formulation for physiological administration, laying the foundation for modulating the local oxidative stress microenvironment."

Page 33 in the revised manuscript: "The antioxidant capacity of the supramolecular complexes was evaluated using the DPPH radical scavenging assay. DPPH powder (Macklin, China) was added into anhydrous ethanol and diluted to 80 μg/mL by ultrasound until it was completely dissolved. Sample solutions (COL, PUE_2/COL, PUE_4/COL, PUE_6/COL, PUE_8/COL, PUE_{10}/COL, PL/COL,

and PL/PUE_{10}/COL) were thoroughly mixed with equivalent volumes of freshly prepared DPPH-ethanol solution. The mixtures were incubated in the dark at room temperature for 60 minutes. The absorbance was then measured at 517 nm. The DPPH radical scavenging rate was calculated using the following formula:

$$\text{DPPH Clearance (\%)} = [1-(A_t-A_b)/A_c] \times 100$$

where A_c is the absorbance of DPPH at 517 nm when no sample was added; A_t is the absorbance at 517 nm after mixing the sample with DPPH for 60 minutes; A_b is the absorbance of the sample without the addition of DPPH at 517 nm."

Page 27 in the revised manuscript: "Based on these findings, we proposed a lysosome-centered immuno-metabolic regulatory mechanism. PUE was internalized via supramolecular-mediated endocytosis and subsequently accumulated within the phagolysosomes. While COL and PL were gradually degraded by enzymes, the C-glycoside structure of PUE conferred exceptional resistance to acid hydrolysis and enzymatic degradation. It allowed PUE to accumulate and persist within the phagolysosomes as an *in-situ* antioxidant component.

Typically, macrophages rely on ROS for bactericidal activity. However, *P. gingivalis* utilizes innate antioxidant defenses to resist the physiological ROS generated by macrophages[69]. Rather than being cleared, intracellular pathogens can actively induce excessive, pathological ROS to promote their intracellular survival[70,71]. This excessive ROS generation directly reduces lysosome membrane stability and triggers lysosomal membrane permeabilization (LMP)[72]. LMP usually causes the loss of bactericidal hydrolases, enabling the pathogen to evade lysosomal degradation and persist intracellularly, while simultaneously serving as a principal driver for macrophage necroptosis[70,71].

By scavenging this pathological ROS burst, PUE directly prevents LMP and ROS-driven necroptosis. Preserving lysosomal membrane integrity supports V-ATPase-mediated maintenance of the acidic pH required for hydrolase activation, while stabilized lysosomal homeostasis further interrupts the LMP-triggered cascade of intracellular metabolic dysregulation[72]. Consequently, the supramolecular functional integration not only facilitated intracellular bacteria elimination, but more importantly, reversed immuno-metabolic dysregulation via the restoration of lysosomal homeostasis, thereby establishing a microenvironment conducive to tissue regeneration."

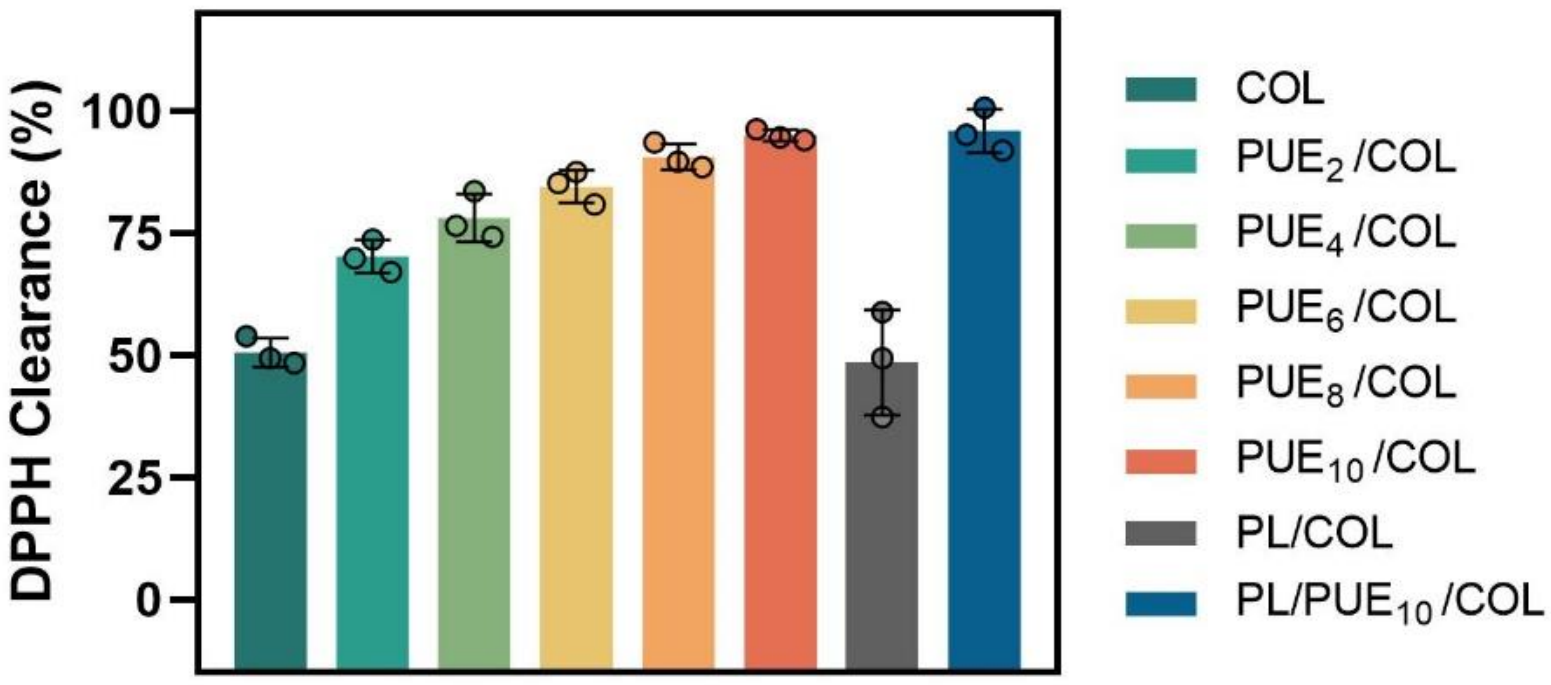

Supplementary Fig. 9. DPPH radical scavenging activity of COL, PUE/COL supramolecular complexes with gradient PUE concentrations (2, 4, 6, 8, 10 mg/mL, denoted as PUE_2/COL to PUE_{10}/COL), PL/COL, and PL/PUE_{10}/COL supramolecular complexes (n=3). All error bars represent mean ± SD. COL recombinant human type I collagen, PL poly-ε-lysine, PUE puerarin, DPPH 2,2-diphenyl-1-picrylhydrazyl. Source data are provided as a Source Data file.

9. Conditioned medium was used to demonstrate the promotion of osteogenesis following immune modulation. However, do the PUE or PL released from the hydrogel possess direct osteogenic effects themselves? It is recommended to supplement an experiment directly co-culturing the hydrogel extract with osteoblasts.

Response:

We sincerely appreciate this constructive suggestion. We agree that clarifying whether the released components possess direct osteogenic properties is essential for a comprehensive understanding of the bone regeneration mechanism.

Following your valuable recommendation, we have supplemented direct co-culture experiments using hydrogel extracts and osteoblasts to evaluate their direct osteogenic effects. As shown in the newly added **Supplementary Fig. 20**, osteoblasts directly co-cultured with extracts from the PUE/COL and PL/PUE/COL hydrogels exhibited significantly enhanced alkaline phosphatase (ALP) staining intensity and protein expression compared to the Blank group. These new results clearly demonstrate that the released PUE indeed possesses direct osteogenic-promoting capabilities. Consequently, the periodontal bone repair observed in our study is driven by a synergistic mechanism including indirect immunomodulatory benefits and direct osteogenic

stimulation from PUE. We have incorporated these new experiments into supplementary information and updated the corresponding discussion in the revised manuscript, as also shown below:

Page 23 in the revised manuscript: "Apart from immune-mediated indirect osteogenesis, we evaluated the direct osteogenic effects of the hydrogels on osteoblasts. As shown in Supplementary Fig. 20, extracts from PUE/COL and PL/PUE/COL enhanced ALP staining intensity and protein expression compared to the Blank group, whereas the PL/COL group showed only a slight improvement. It suggests that in addition to the immunomodulatory role, PUE also directly promotes osteogenic differentiation, thereby facilitating local bone repair through a multidimensional therapeutic approach."

Page 40 in the revised manuscript: "To evaluate the direct osteogenic potential, hydrogel extracts were prepared by incubating the sterilized hydrogels in culture medium at 37 °C for 24 hours, as described above. MC3T3-E1 cells were seeded and cultured for 24 hours, before the culture medium was replaced with osteogenic induction medium supplemented with the respective hydrogel extracts. After culturing for 7 days, the cells were subjected to ALP staining and immunofluorescence staining for ALP, following the protocols described previously."

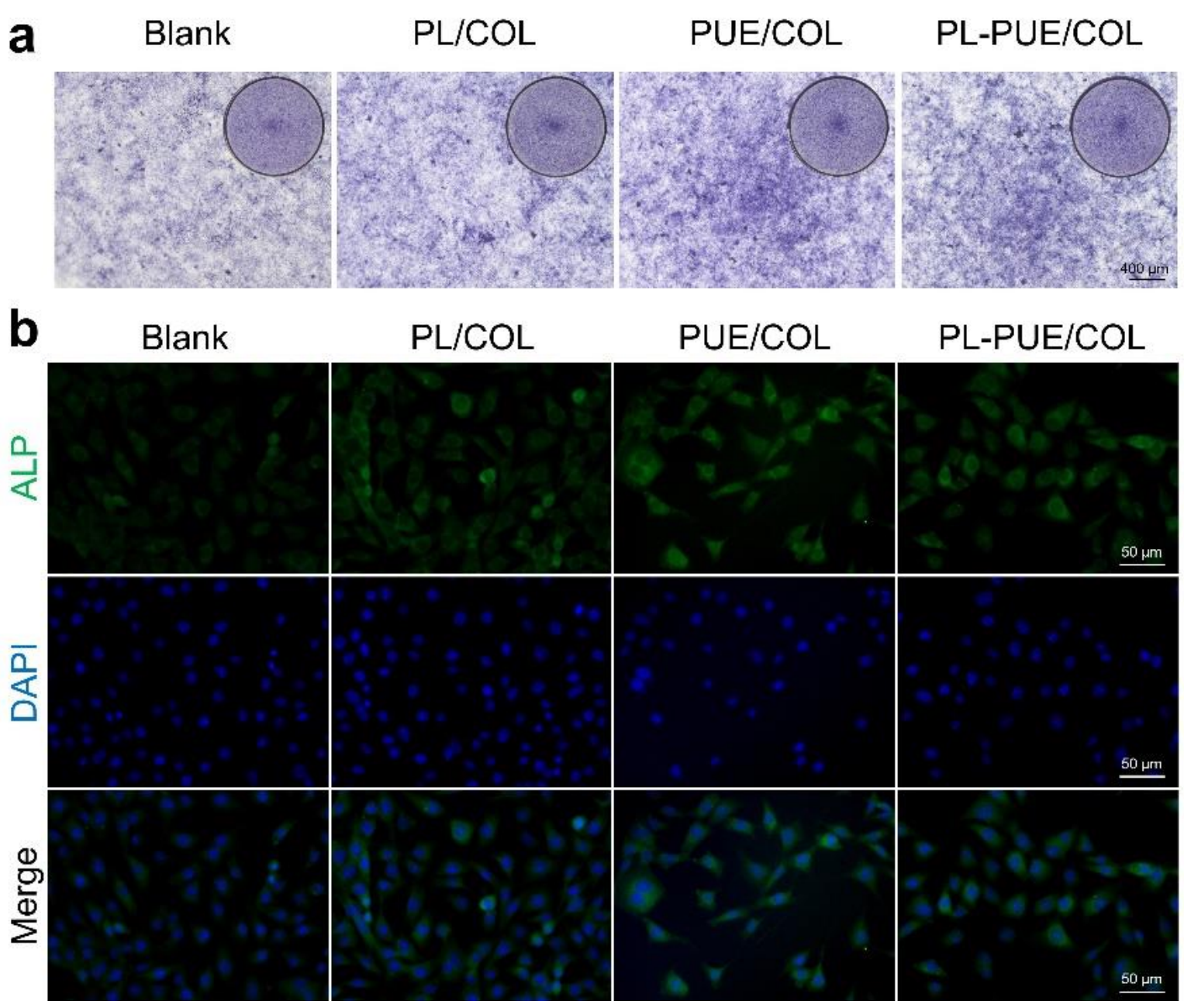

Supplementary Fig. 20. Representative images of alkaline phosphatase (ALP) staining and immunofluorescence staining for ALP in MC3T3-E1 cells, treated with PL/COL, PUE/COL, and PL/PUE/COL hydrogels. COL recombinant human type I collagen, PL poly-ε-lysine, PUE puerarin,

ALP alkaline phosphatase.

10. The PL4/COL group exhibits toxicity, whereas the toxicity of PL/PUE/COL is significantly reduced. Is this simply because the PL concentration was halved, or is it because the addition of PUE buffered the cationic toxicity of PL?

Response:

We sincerely thank the reviewer for the question. Our results indicate that the reduction in toxicity is primarily attributed to the optimized concentration of PL, rather than a buffering effect from PUE.

The biological compatibility assays (Fig. 4i and Supplementary Fig. 16) demonstrate that reducing the PL concentration from 4 mg/mL to 2 mg/mL significantly improved cell viability. Furthermore, no further reduction in toxicity was observed when PUE was added to the PL/COL system, suggesting that the concentration of PL is the main factor for biocompatibility.

At the molecular level, the toxicity of PL is mainly considered from its high density of cationic groups. Our molecular dynamics simulations (Supplementary Fig. 5a) further clarify the relationship between PUE and PL. The results show that PUE does not exhibit strong Coulomb or Lennard-Jones interactions with the cationic groups of PL. This suggests that PUE does not significantly neutralize or shield the cationic charge of PL, thereby preserving the availability of the active sites for antibacterial activity while confirming that PUE does not reduce toxicity through charge buffering. We have added these clarifications to the revised manuscript.

Page 40 in the revised manuscript: "Notably, the cell viability of the PL/COL group was comparable to that of the PL/PUE/COL group, indicating that the reduced toxicity was primarily due to the lower PL concentration, and the incorporation of PUE did not further alter the cytotoxicity. Therefore, as the synergistic supramolecular complex of PL and PUE not only enhances antibacterial efficiency but also ensures biocompatibility, we used the PL with the concentration of 2 mg/mL in the following experiments."

11. For the in vivo experiments, the Masson staining and immunohistochemistry results need to be

completed by including the PL/COL, PUE/COL, and Mino comparison groups.

Response:

Thank you for your invaluable comments and suggestions on how to enhance the quality of our manuscript. We agree that including these comprehensive comparison groups is essential for a thorough evaluation of the *in vivo* therapeutic efficacy.

Following your recommendation, we have supplemented the *in vivo* experiments by adding the PL/COL, PUE/COL, and Minocycline (Mino) groups for both Masson staining and immunohistochemistry analyses (**Supplementary Fig. 22-24**). The updated results further demonstrate the synergistic effect of the PL/PUE/COL supramolecular hydrogel in promoting periodontal collagen deposition and modulating the inflammatory microenvironment, as compared to the single-component controls and the Minocycline treatment.

Furthermore, to ensure the consistency and rigor of the semi-quantitative statistical analysis, all original groups were re-evaluated with the newly added groups to guarantee reliable comparability. All corresponding histological images and semi-quantitative analyses have been completed and incorporated into the revised manuscript and supplementary information.

Page 30 in the revised manuscript: "To further evaluate the periodontal tissues, Masson staining was performed to assess collagen deposition and organization (Supplementary Fig. 22). The Blank group displayed an intact periodontal tissue structure, with collagen fibers arranged in an orderly and dense manner. In the Periodontitis group, collagen fibers exhibited a disordered and irregular structure, indicative of a disrupted collagenous fiber arrangement due to local inflammation. Similar to the Periodontitis group, PL/COL, PUE/COL, and Mino groups exhibited disorganized collagen deposition, suggesting that single-component hydrogels or conventional antibiotics are insufficient to restore the connective tissue structure in an inflammatory milieu. In contrast, following the treatment with PL/PUE/COL, the periodontal tissues displayed denser and more orderly collagenous fibers.

To evaluate the local inflammatory and regenerative microenvironment, immunohistochemical staining of TNF-α and TGF-β around the periodontal microenvironment was performed. As shown in Supplementary Fig. 23a, compared to the elevated TNF-α expression in the Periodontitis group, all treatments attenuated the inflammatory marker to varying degrees. While PL/COL and minocycline provided partial reductions, both PUE-containing hydrogels (PUE/COL and

PL/PUE/COL) more effectively downregulated TNF-α to levels comparable to the Blank group (Supplementary Fig. 23b). We further investigated the local expression levels of TGF-β, a pivotal mediator of tissue repair, to determine the underlying regenerative capacity of the periodontal microenvironment (Supplementary Fig. 24a). Compared to Blank group, TGF-β levels were significantly reduced in the Periodontitis group. While PL/COL, PUE/COL, and minocycline provided only a moderate recovery of TGF-β, the PL/PUE/COL treatment effectively restored its expression, showing no significant difference from the Blank group (Supplementary Fig. 24b). These findings indicate that while PUE attenuates inflammation, the synergistic effect of the supramolecular system is essential to re-establish a microenvironment favorable for tissue regeneration."

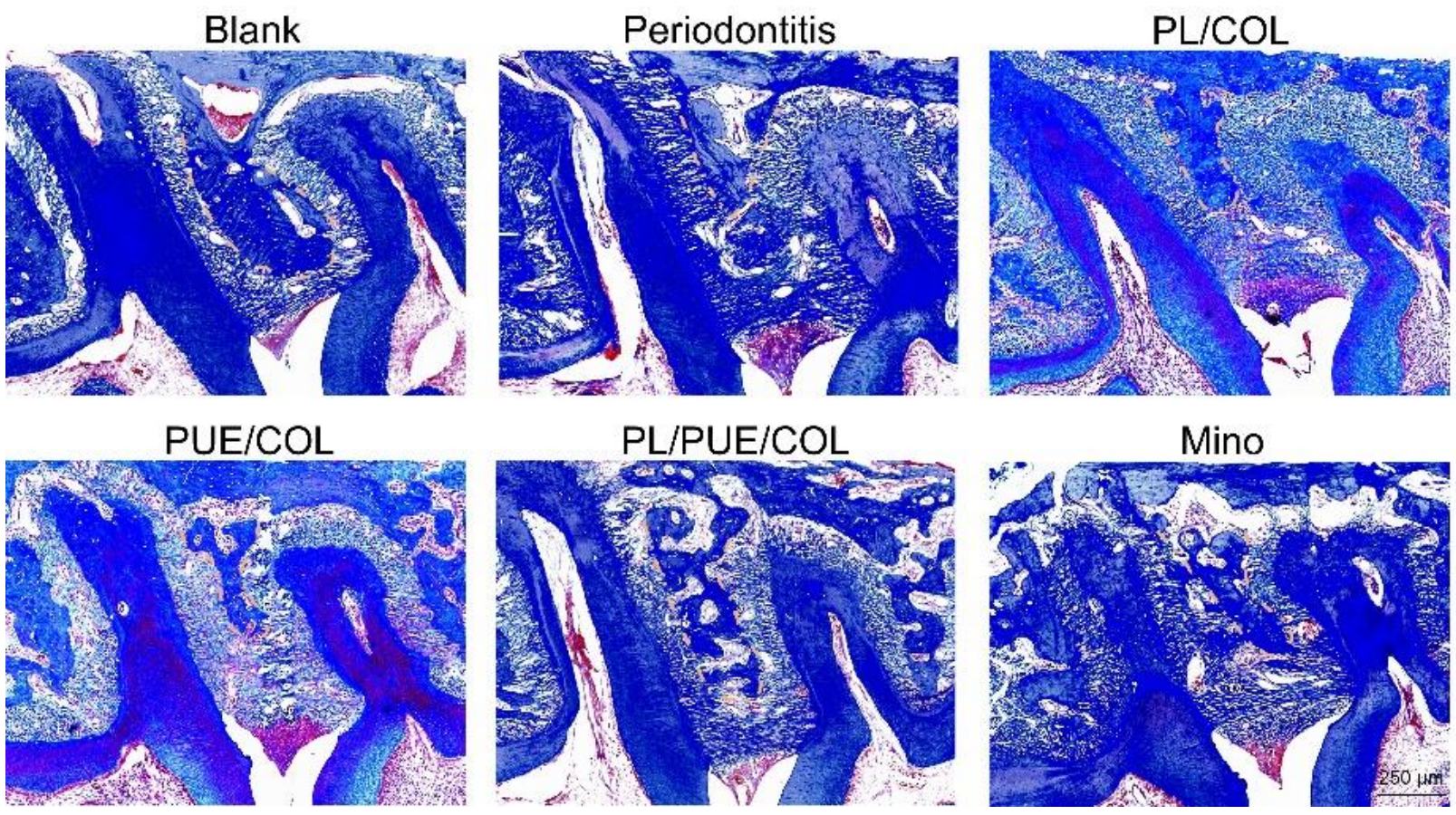

Supplementary Fig. 22. Masson staining of the area between the maxillary first and second molars. Dashed lines indicate the alveolar bone within the lesion area.

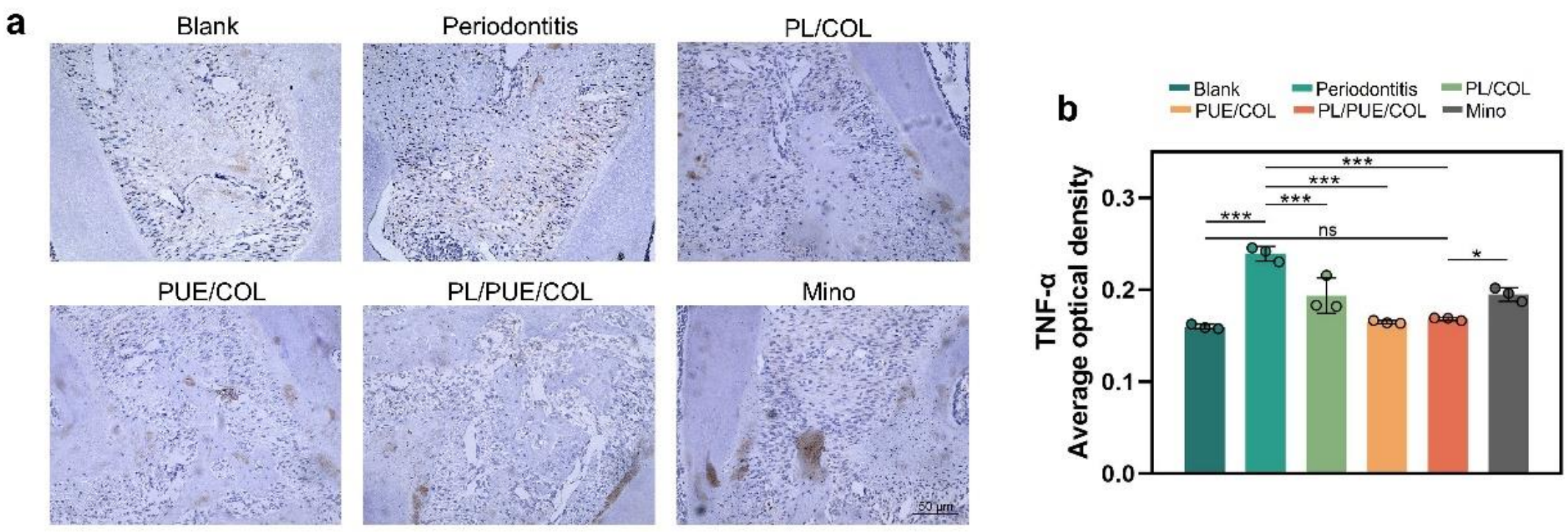

Supplementary Fig. 23. (a) Representative images of immunohistochemical staining for TNF-α in periodontal tissue. (b) Semiquantitative analysis of the average optical density (n=3). All error bars represent mean ± SD. P values were calculated by one-way ANOVA followed by Tukey's post hoc

test (*P < 0.05; ***P < 0.001; ns, not significant). TNF-α tumor necrosis factor-α. Source data are provided as a Source Data file.

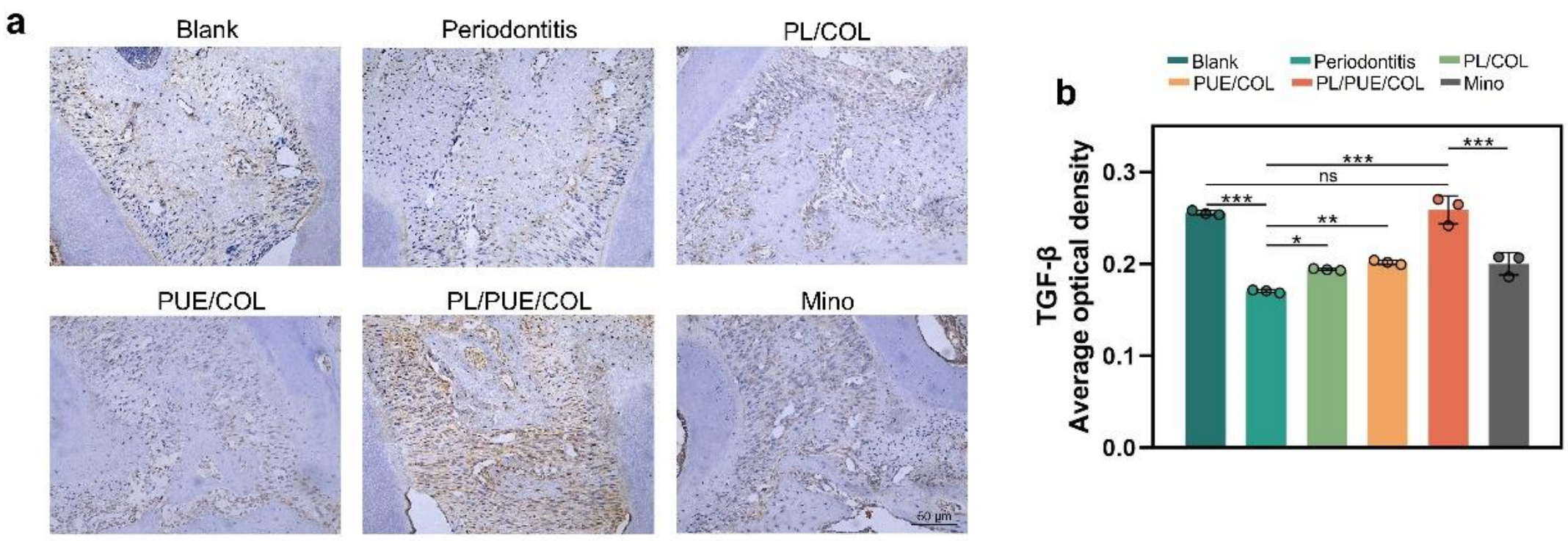

Supplementary Fig. 24. (a) Representative images of immunohistochemical staining for TGF-β in periodontal tissue. (b) Semiquantitative analysis of the average optical density (n=3). All error bars represent mean ± SD. P values were calculated by one-way ANOVA followed by Tukey's post hoc test (*P < 0.05; **P < 0.01; ***P < 0.001; ns, not significant). TGF-β transforming growth factor-β. Source data are provided as a Source Data file.

12. The "Dual Compartment" concept is a core concept in the title of the article, but it is not adequately reflected in the Discussion section.

Response:

We sincerely thank the reviewer for pointing out this critical deficiency. As the core conceptual framework of our study, the Dual Compartment strategy should be explicitly elaborated.

To address your valuable suggestion, we have added a paragraph in the revised Discussion section to systematically summarize this concept. We clarified how the supramolecular system works synergistically both extracellularly and intracellularly, and how to bridge initial pathogen clearance with subsequent osteo-immune remodeling. We believe this addition strengthens the conceptual depth of the manuscript. The detailed elaboration has been incorporated into the revised manuscript as follows:

Page 32 in the revised manuscript: "The PL/PUE/COL supramolecular hydrogel achieves effective pathogen clearance through a dual-compartment strategy. Extracellularly, it eliminates

pathogens through a synergistic mechanism where PL damages the bacterial outer membrane and PUE disrupts the inner membrane, reducing the bacterial burden and impairing pathogen invasiveness. Intracellularly, by protecting the lysosomal membrane from bacteria-induced oxidative damage, it restores the lysosomal homeostasis required for effective bactericidal function. The restoration of lysosomal activity not only helps eradicate intracellular bacteria, but also reverses the bacteria-mediated immune-metabolic dysregulation. Such an integrated system bridges pathogen clearance with osteo-immune remodeling, providing a promising strategy to disrupt the vicious cycle of chronic inflammation and bacterial persistence in periodontitis."

13. This work design has significant value in application and translational research, and warrants in-depth and systematic investigation to develop a distinctive framework. Thus, it will be better if the authors discuss its limitation in current study. This will really strengthen their study and will help the readers understand more about their future study of the supramolecular materials.

Response:

We sincerely appreciate the reviewer's encouraging comments and constructive suggestions about our work. We agree that a clear discussion of the current limitations and future directions will strengthen the manuscript and provide a broader perspective on the development of these supramolecular materials.

We have expanded the Discussion section to clarify the current limitations. In detail, we noted that the regulatory pathways of lysosomal homeostasis, the precise intracellular trafficking kinetics, and subcellular interactions require further elucidation. Furthermore, we have outlined our future plan to integrate advanced nanotechnology for achieving cell- and organelle-specific targeting, thereby pushing this system closer to clinical translation. The detailed discussion has been incorporated into the revised manuscript as follows:

Page 32 in the revised manuscript: "While the supramolecular co-assembly effectively overcomes PUE's inherent constraints of solubility and permeability, certain limitations remain. The specific regulatory pathways of lysosomal homeostasis need to be further elucidated. The precise intracellular trafficking kinetics and the specific interactions of these complexes at the subcellular structural level also warrant deeper mechanistic investigation. Drawing inspiration from advanced

platforms that leverage precise molecular architectures for multi-level biological activities, future studies could integrate our easily fabricated and low-cost supramolecular system with advanced nanotechnology to construct nanomedicines with precisely controlled strategies[73–76]. Building upon this co-assembly mechanism, targeted optimization and functional modification of the constituent components will be essential to achieve cell- and organelle-specific targeting, thereby further advancing the clinical translation of this system."

14. There are inconsistencies in multiple places throughout the text; for example, "MitoSOX" and "Heatmap" appear in different formats in different sections.

Response:

We sincerely apologize for these formatting inconsistencies and are highly grateful to the reviewer for the careful reading of our manuscript.

We have thoroughly checked the entire manuscript, including the main text, figure legends, and the supplementary information. All instances of "MitoSOX" and "heat map" have been corrected to ensure consistency. Furthermore, we have checked and unified other professional terms, abbreviations, and italicizations.

15. There is a formatting error in Reference [69]; please correct it.

Response:

We sincerely apologize for the formatting error in the reference. We have corrected the format of this reference (which is now Reference [72] in the revised manuscript) to strictly follow the journal's guidelines. Additionally, we have performed a comprehensive check of all references in the revised manuscript to ensure formatting correctness.

Reviewer #2：

This study presents a highly innovative and well-executed investigation on the tharapy of periodontitis by developing an injectable supramolecular hydrogel (PL/PUE/COL) through the co-assembly of collagen, poly-ε-lysine,and puerarin. Authors addresses a critical challenge in periodontitis therapy: the persistence of intracellular bacteria that evade immune clearance and induce lysosomal dysfunction. Overall, the study is comprehensive, spanning materials design, mechanistic elucidation, and in vivo validation. In my opinion, after addressing the following minor concerns on presentation, methodology, discussion and references, it is suitable for publication.

Response to the general comment:

We are very grateful for your evaluation of our study. Your constructive feedback has been instrumental in refining our work. Guided by your insights, we have thoroughly revised the manuscript and supplementary materials to address each of your specific concerns. We believe these modifications have strengthened the clarity and depth of our paper, and we sincerely thank you for your time and valuable guidance.

1. In the Abstract and Conclusion, the “dual-compartment” concept is clearly described. However, it would be beneficial to emphasize that the restoration of lysosomal homeostasis serves as the mechanistic bridge between extracellular bacterial reduction and intracellular immune reprogramming. This would further integrate the two compartments into a unified therapeutic axis.

Response:

We sincerely appreciate your insightful suggestion, which significantly strengthens the conceptual framework of our study.

To address your valuable comment, we have added a paragraph in the Discussion section to systematically summarize the dual-compartment strategy. In this revised text, we emphasize how the restoration of lysosomal homeostasis serves as the link between extracellular pathogen killing and intracellular immuno-metabolic reprogramming, thereby disrupting the vicious cycle of chronic inflammation and bacterial persistence in periodontitis. The corresponding revisions have been incorporated into the revised manuscript as follows:

Page 32 in the revised manuscript: "The PL/PUE/COL supramolecular hydrogel achieves effective pathogen clearance through a dual-compartment strategy. Extracellularly, it eliminates pathogens through a synergistic mechanism where PL damages the bacterial outer membrane and PUE disrupts the inner membrane, reducing the bacterial burden and impairing pathogen invasiveness. Intracellularly, by protecting the lysosomal membrane from bacteria-induced oxidative damage, it restores the lysosomal homeostasis required for effective bactericidal function. The restoration of lysosomal activity not only helps eradicate intracellular bacteria, but also reverses the bacteria-mediated immune-metabolic dysregulation. Such an integrated system bridges pathogen clearance with osteo-immune remodeling, providing a promising strategy to disrupt the vicious cycle of chronic inflammation and bacterial persistence in periodontitis."

2. Define all abbreviations (e.g., MTG, CEJ-ABC, BV/TV, TCIRG1, LAMP1, CTSD) at first appearance in the main text and figure legends.

Response:

We appreciate your meticulous review and helpful suggestion regarding the abbreviations. We fully agree that clearly defining all abbreviations is essential for the readability and clarity of the manuscript.

In response to your comment, we have carefully checked the entire manuscript and supporting information. We have ensured that abbreviations (including MTG, CEJ-ABC, BV/TV, TCIRG1, LAMP1, CTSD, among others) are defined at their first appearance in the main text. Furthermore, we have listed and defined all relevant abbreviations at the end of each figure legend in both the main manuscript and the supporting information.

The corresponding revisions have been incorporated throughout the revised manuscript and supplementary information highlighted in yellow.

3. Add brief explanatory notes for Supplementary Figures 1-18 in the main text.

Response:

We thank the reviewer for this helpful suggestion. We agree that providing brief explanatory context for the supplementary data within the main text enhances the readability and clarity of our

results.

We have carefully revised the Results section to include more detailed descriptions of the experimental methods and findings represented by Supplementary Figures at each citation point. Instead of simple mention, we have integrated these explanatory notes directly into the narrative description to make the supplementary information more readable for the readers. The corresponding revisions are distributed throughout the Results section of the revised manuscript and are highlighted in yellow.

4. Supplement strain, sex, age, weight of mice used in the in vivo model.

Response:

We appreciate your valuable suggestion. Providing detailed information about the experimental animals is crucial for the reproducibility of *in vivo* studies.

Following your advice, we have explicitly improved the strain, sex, age, and weight of the mice used in our periodontitis model. The corresponding details have been incorporated into the Methods section of the revised manuscript, as also shown below:

Page 41 in the revised manuscript: "6-week-old C57BL/6 mice (male, 18-20 g) were used to construct *in vivo* periodontitis model."

5. Add cell source, species, and passage number for RAW264.7 and MC3T3-E1 cells.

Response:

We appreciate your careful review and attention to these experimental details. We agree that providing comprehensive information regarding the cell lines is essential for the reproducibility of our *in vitro* studies.

As requested, we have further supplemented the specific cell source, species for both the RAW264.7 and MC3T3-E1 cell lines used in our experiments.

The corresponding additions have been incorporated into the Methods section of the revised manuscript, as also shown below:

Page 36 in the revised manuscript: "Mouse macrophage RAW 264.7 cell line were obtained from the ATCC (Manassas, VA, USA)."

Page 40 in the revised manuscript: "Mouse MC3T3-E1 pre-osteoblastic cells were obtained from the ATCC (Manassas, VA, USA), and were cultured in α-MEM medium supplemented with 10% FBS."

6. In Figure 3H, the 3D surface plots are visually informative. Please supplement this with a quantitative bar graph of fluorescence intensity to allow for direct statistical comparison of membrane depolarization between groups.

Response:

We are grateful for this constructive suggestion, which significantly enhances the presentation of our bacterial membrane depolarization analysis.

Supplementing the 3D surface plots with a quantitative bar graph provides more direct statistical comparison. Accordingly, we have performed the quantitative analysis of the DiBAC4(3) fluorescence intensity. The quantitative results clearly demonstrated a statistically significant increase in fluorescence for the PL/PUE/COL hydrogel group compared to the COL, PL/COL and PUE/COL groups, supporting the bacterial membrane depolarization triggered by the synergetic effect of PL/PUE/COL supramolecular system.

The quantitative bar graph has been added to the supporting information as **Supplementary Fig. 15**. The corresponding revisions have been incorporated into the revised manuscript as follows:

Page 17 in the revised manuscript: "To further validate this hypothesis, we assessed the bacterial transmembrane potential using the voltage-sensitive dye bis-(1,3-dibutylbarbituric acid)-trimethine oxonol (DiBAC4(3)) (Fig. 3h). The PL/PUE/COL group exhibited intense green fluorescence, indicative of severe membrane depolarization. The PUE/COL group and PL/COL group displayed only faint signals, while the Blank group showed negligible fluorescence. Compared to the PL/COL and PUE/COL groups, the PL/PUE/COL group exhibited a significantly higher mean fluorescence intensity (Supplementary Fig. 15). It can be explained by the specific localization of the electron transport chain on the IM. In the process, PL disrupts the OM barrier, thereby enabling PUE to effectively perturb the metabolic activity of the IM."

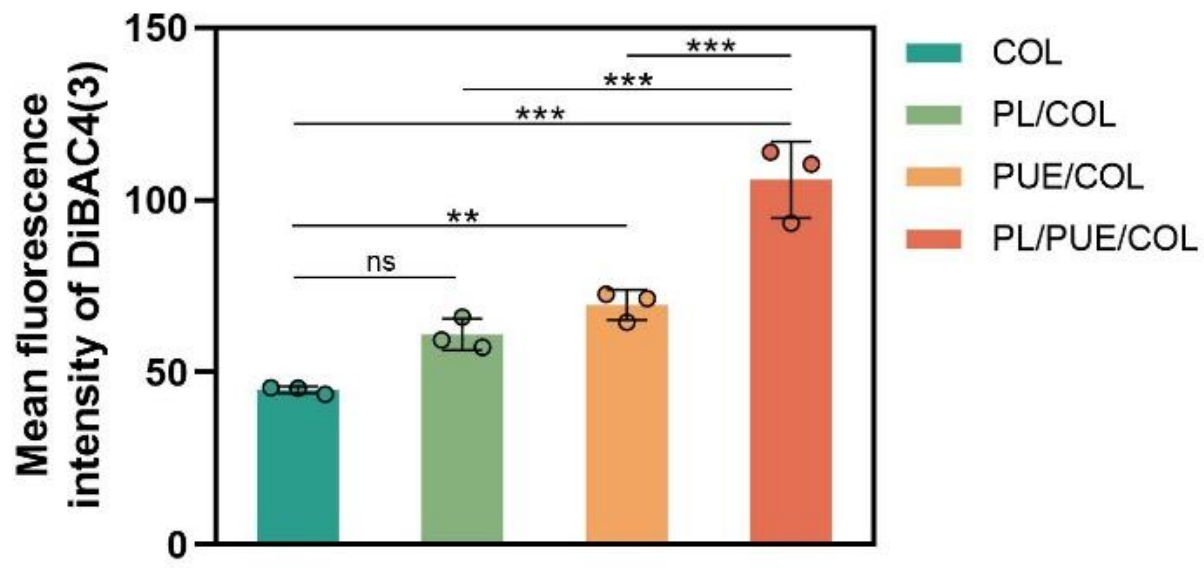

Supplementary Fig. 15. Statistical analysis of the relative fluorescence density in Fig. 3h (n=3). All error bars represent mean ± SD. P values were calculated by one-way ANOVA followed by Tukey's post hoc test (*P < 0.05; **P < 0.01; ***P < 0.001; ns, not significant). Source data are provided as a Source Data file.

7. In Figure 6G (Lysosome-bacteria colocalization), authors are suggested to add a quantitative colocalization analysis (e.g., Pearson's or Mander's coefficient) to support the visual observation that PUE/COL and PL/PUE/COL restore phagolysosomal fusion.

Response:

We sincerely thank you for the constructive suggestion to strengthen the colocalization analysis. However, applying global correlation coefficients to our infection model presents a mathematical limitation. In our system, the host cell lysosomal network (red fluorescence) is extensively distributed throughout the cytoplasm, whereas the intracellular *P. gingivalis* (green fluorescence) exists as highly localized distribution. The substantial disparity in spatial areas introduces extensive uninfected lysosomal pixels into the global calculation. Consequently, mathematical algorithms for global coefficients would severely dilute the correlation values, failing to accurately represent the results.

To provide more suitable and visualized evaluation of the bacteria-lysosome colocalization without the interference of spatial asymmetry, guided by previous studies, we utilized spatial fluorescence intensity profiling (X.Wang, et al., *Adv. Funct. Mater*, 2025). We visualized the spatial relationship between intracellular FITC-labeled *P. gingivalis* and lysosomes stained with LysoTracker Red. To validate the colocalization, spatial fluorescence intensity profiles were plotted along the paths indicated by the white dashed arrows. As shown in the revised **Figure 6g**, after

treatment with PUE/COL and PL/PUE/COL, the fluorescence intensity profiles of both channels exhibited a highly synchronized spatial overlap. The peak-to-peak correspondence serves as direct evidence of colocalization. Crucially, since LysoTracker selectively accumulates in highly acidic compartments, its spatial colocalization with the bacteria indicated restored lysosomal acidification.

We believe this targeted approach accurately addresses the core concern regarding the verification of phagolysosomal colocalization. The corresponding revisions have been incorporated into the revised manuscript as follows:

Page 26-27 in the revised manuscript: "Subsequently, we investigated whether it translated to effective phagolysosomal fusion at the cellular level. We visualized the spatial relationship between intracellular FITC-labeled *P. gingivalis* and lysosomes stained with LysoTracker Red (Fig. 6g). To validate the colocalization, spatial fluorescence intensity profiles were plotted along the paths indicated by the white dashed arrows. In the untreated *P. gingivalis* infection group (*P.g* group) and the PL/COL group, the fluorescence peaks of bacteria (green) and lysosomes (red) were spatially mismatched. Since LysoTracker selectively accumulates in acidic compartments, this separation indicated that *P. gingivalis* evaded phagolysosome fusion or impaired the acidification process. In contrast, after treatment with PUE/COL and PL/PUE/COL, the fluorescence intensity profiles of both channels exhibited a highly synchronized spatial overlap, suggesting a restoration of phagolysosome structure and function. Crucially, since LysoTracker selectively accumulates in highly acidic compartments, its spatial colocalization with the bacteria indicated restored lysosomal acidification. Combined with the molecular upregulation of essential lysosomal proteases (CTSD) and structural membrane proteins (LAMP1), these findings confirm that the PUE-based supramolecular hydrogel revives the lysosomal bioactivity."

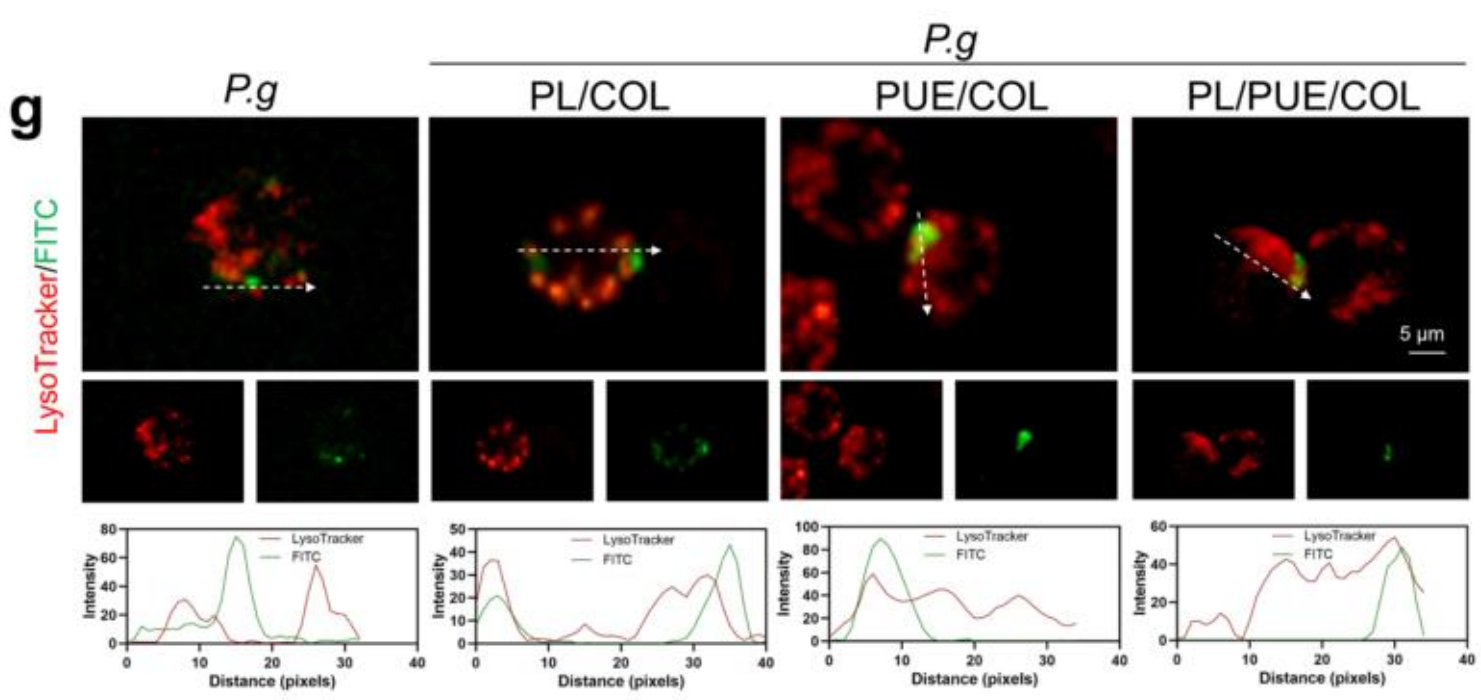

Fig. 6 Mechanism of PL/PUE/COL-mediated immuno-metabolic regulation. (g) Co-localization images of lysosomes and bacteria.

8. Please standardize descriptions of anaerobic culture conditions (gas mixture, time, temperature).

Response:

We thank the reviewer for the careful observation. In accordance with your suggestion, we have now standardized the descriptions of the anaerobic culture conditions throughout the revised manuscript. Specifically, the parameters regarding the gas mixture, and temperature for all anaerobic bacterial cultures have been explicitly detailed and unified, and the incubation time was emphasized at the specific experimental description.

The corresponding revisions have been incorporated into the Methods section of the revised manuscript as follows:

Page 35-36 in the revised manuscript: "To evaluate the antibacterial activity, *P. gingivalis* (ATCC 33277) or *F. nucleatum* (ATCC 25586) suspensions (300 μL, 1 × 10^6 CFU/mL) were incubated with 100 μL of sterilized hydrogels in 48-well plate under anaerobic condition (10% H_2, 10% CO_2, 80% N_2, 37°C). The following seven groups were used: blank control group (untreated bacteria), labeled as "Blank"; COL group (pure collagen hydrogel), labeled as "COL"; collagen loaded with 2 mg/mL PL, marked as "PL/COL"; collagen loaded with 4 mg/mL PL, marked as "PL_4/COL"; collagen loaded with 10 mg/mL PUE, labeled as "PUE/COL"; collagen loaded with 2 mg/mL PL and 10 mg/mL PUE, labeled as "PL/PUE/COL"; treatment of 20 μg/mL minocycline hydrochloride (Apex Bio, USA), labeled as "Mino".

The OD values of the bacterial suspensions were measured at 600 nm every 24 hours. After 72 hours of incubation in an anaerobic environment, the obtained bacteria suspensions were diluted, before 100 μL were taken to spread on brain heart infusion (BHI) blood agar plates. After culturing in an anaerobic environment at 37°C for 96 hours, the bacterial colonies on the plates were photographed, and the antibacterial efficiency was calculated for quantitative analysis by the following formula:

$$\text{Antibacterial ratio (\%)} = (N_0 - N_1)/ N_0 \times 100\%$$

Here, N_0 represented the number of bacteria without any treatment while N_1 represented the number of bacteria cultured with different hydrogels.

For bacterial live/dead assay, samples were collected and treated with live/dead bacterial

staining kit (Apex Bio, USA), according to the manufacturer's instructions. The staining operation proceeded under dark conditions. Live bacteria showed green fluorescence and dead bacteria showed red fluorescence. The stained bacteria were observed using a fluorescence microscope (Leica, DMi8, Germany). To observe the morphology of the bacteria cocultured with hydrogels, the bacterial precipitation was treated with 2.5% Glutaraldehyde EM Grade (Solarbio, China) at 4 °C overnight. Then, the samples were dehydrated in a gradient ethanol solution of 30%, 50%, 75%, 85%, 95% and 100% sequentially. Morphology observation was under SEM (JEOL Ltd., Japan) with the exposure parameters of 20 kV.

A dual-species infection model was established as previously reported[83]. *P. gingivalis* (ATCC 33277) and *F. nucleatum* (ATCC 25586) suspensions (1×10^6 CFU/mL) were mixed at a 1:1 volume ratio to acquire dual-species bacterial suspension. Subsequently, 300 µL of the suspension was further incubated with 100 µL of sterilized hydrogels in a 48-well plate at 37°C under anaerobic conditions. The OD values at 600 nm were monitored every 24 hours over a 72-hour period."

9. In the periodontitis model, periodontal ligament stem cells (PDLSCs) play a critical role in tissue remodeling. Could you evaluate the effects of PUE, PUE/COL, and PL/PUE/COL on the osteogenic activity of PDLSCs?

Response:

We sincerely appreciate this constructive suggestion. We fully agree that clarifying whether the released components possess direct osteogenic properties is essential for a comprehensive understanding of the bone regeneration mechanism during periodontal tissue remodeling.

Following your valuable recommendation, we have supplemented *in vitro* experiments using hydrogel extracts to evaluate their direct osteogenic effects. While we acknowledge the critical role of PDLSCs in periodontal regeneration, we specifically selected the murine pre-osteoblast cell line MC3T3-E1 for this evaluation, since we have utilized the murine macrophage cell line RAW264.7 in our *in vitro* co-culture system. To maintain species homology and prevent any potential cross-species incompatibilities that might confound the immunomodulatory and osteogenic behavior, using a matched murine cell line (MC3T3-E1) was deemed the suitable choice.

As shown in the newly added **Supplementary Fig. 20**, MC3T3-E1 cells directly cultured with extracts from the PUE/COL and PL/PUE/COL hydrogels exhibited significantly enhanced alkaline

phosphatase (ALP) staining intensity and protein expression compared to the Blank group. These new results clearly demonstrate that the released PUE indeed possesses direct osteogenic-promoting capabilities. Consequently, the periodontal bone repair observed in our study is driven by a synergistic mechanism, encompassing both indirect immunomodulatory benefits and direct osteogenic stimulation from PUE.

We have incorporated these new experiments into supplementary information and updated the corresponding discussion in the revised manuscript, as shown below:

Page 23 in the revised manuscript: "Apart from immune-mediated indirect osteogenesis, we evaluated the direct osteogenic effects of the hydrogels on osteoblasts. As shown in Supplementary Fig. 20, extracts from PUE/COL and PL/PUE/COL enhanced ALP staining intensity and protein expression compared to the Blank group, whereas the PL/COL group showed only a slight improvement. It suggests that in addition to the immunomodulatory role, PUE also directly promotes osteogenic differentiation, thereby facilitating local bone repair through a multidimensional therapeutic approach."

Page 40 in the revised manuscript: "To evaluate the direct osteogenic potential, hydrogel extracts were prepared by incubating the sterilized hydrogels in culture medium at 37 °C for 24 hours, as described above. MC3T3-E1 cells were seeded and cultured for 24 hours, before the culture medium was replaced with osteogenic induction medium supplemented with the respective hydrogel extracts. After culturing for 7 days, the cells were subjected to ALP staining and immunofluorescence staining for ALP, following the protocols described previously."

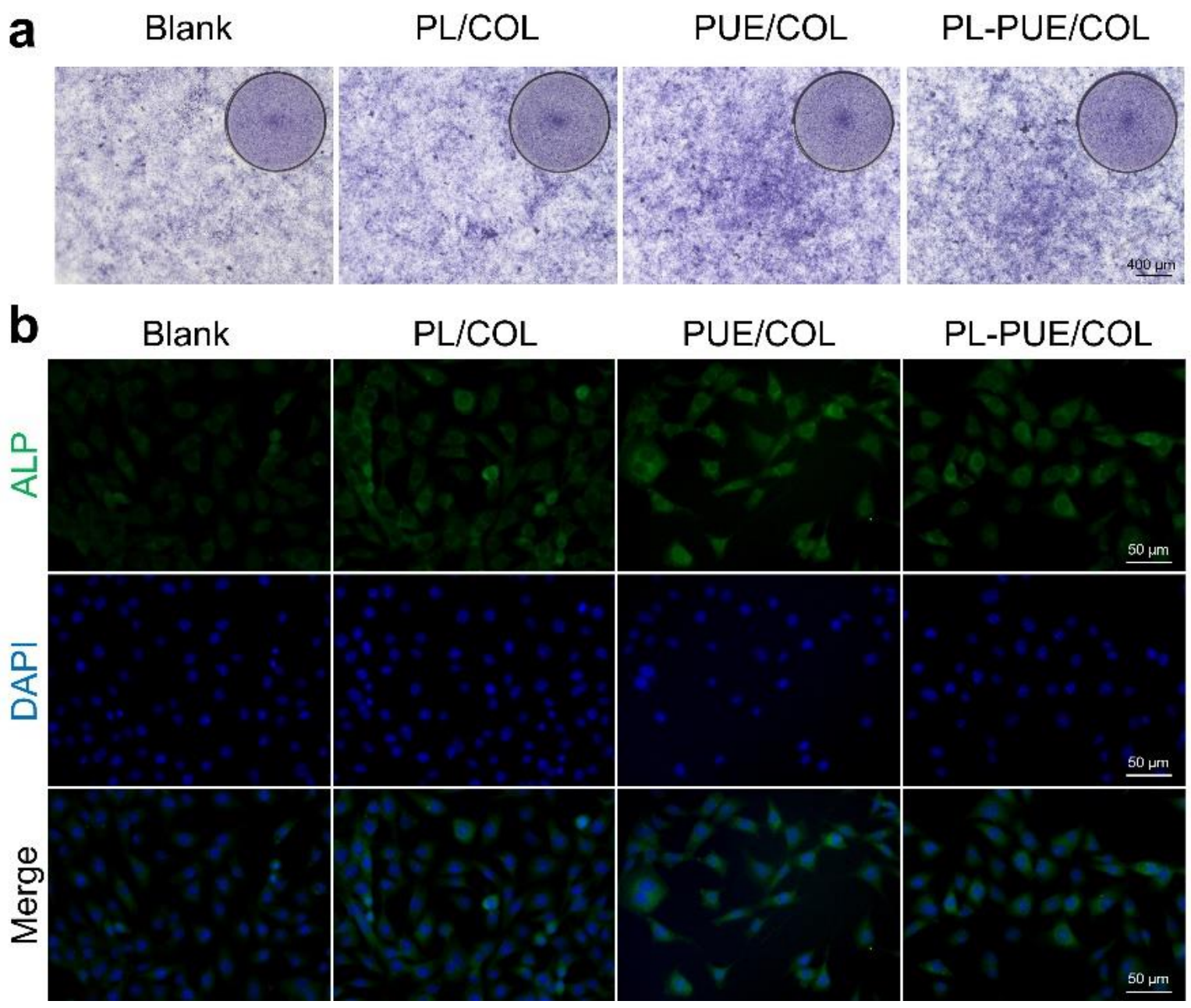

Supplementary Fig. 20. Representative images of alkaline phosphatase (ALP) staining and immunofluorescence staining for ALP in MC3T3-E1 cells, treated with PL/COL, PUE/COL, and PL/PUE/COL hydrogels. COL recombinant human type I collagen, PL poly-ε-lysine, PUE puerarin, ALP alkaline phosphatase.

10. Please supplement the data on the in vitro antioxidant capacity of PUE and its composite materials.

Response:

We appreciate the reviewer's constructive suggestion to further characterize the *in vitro* antioxidant properties of our materials.

We completely agree with the necessity to clarify the antioxidant contribution of PUE. In this study, we did not directly evaluate the antioxidant capacity of free PUE, primarily due to its extremely poor water solubility. Free PUE rapidly undergoes crystalline aggregation and precipitation in aqueous solutions, which disturbs a parallel comparison with our supramolecular complexes (where PUE is uniformly dispersed at the molecular level in aqueous solutions) within an equivalent environment.

To elucidate the core antioxidant role of PUE within the supramolecular complex, we established a gradient of supramolecular complexes with varying PUE concentrations (from

PUE_2/COL to PUE_{10}/COL) for validation. The DPPH radical scavenging assay demonstrated that as the PUE content in the complexes increased, the radical clearance rate exhibited a concentration-dependent enhancement (**Supplementary Fig. 9**). This concentration gradient data verifies that the superior *in vitro* antioxidant capacity of the supramolecular complex is directly attributed to the dose contribution of PUE.

We have incorporated the new data into the supplementary information and updated the corresponding results in the revised manuscript as follows:

Page 27 in the revised manuscript: "Crucially, the supramolecular co-assembly did not compromise the intrinsic bioactivity of PUE. The antioxidant capacity was assessed via the 2,2-diphenyl-1-picrylhydrazyl (DPPH) radical scavenging assay (Supplementary Fig. 9). The radical clearance rate of the PUE/COL groups increased in a concentration-dependent trend, even approaching nearly 100% at the PUE concentrations of 8 and 10 mg/mL. Furthermore, the PL/PUE_{10}/COL supramolecular complex also exhibited an excellent scavenging efficacy comparable to that of PUE_{10}/COL, significantly beyond the PL/COL group and COL group. It demonstrates that PUE retains its antioxidant capability when integrated into the supramolecular complex. More importantly, by overcoming the inherent aqueous insolubility which practically impedes the use of free PUE in physiological fluids, the supramolecular strategy provides an effective formulation for physiological administration, laying the foundation for modulating the local oxidative stress microenvironment."

Page 33 in the revised manuscript: "The antioxidant capacity of the supramolecular complexes was evaluated using the DPPH radical scavenging assay. DPPH powder (Macklin, China) was added into anhydrous ethanol and diluted to 80 μg/mL by ultrasound until it was completely dissolved. Sample solutions (COL, PUE_2/COL, PUE_4/COL, PUE_6/COL, PUE_8/COL, PUE_{10}/COL, PL/COL, and PL/PUE_{10}/COL) were thoroughly mixed with equivalent volumes of freshly prepared DPPH-ethanol solution. The mixtures were incubated in the dark at room temperature for 60 minutes. The absorbance was then measured at 517 nm. The DPPH radical scavenging rate was calculated using the following formula:

$$\text{DPPH Clearance (\%)} = [1-(A_t-A_b)/A_c] \times 100$$

where A_c is the absorbance of DPPH at 517 nm when no sample was added; A_t is the absorbance at 517 nm after mixing the sample with DPPH for 60 minutes; A_b is the absorbance of the sample

without the addition of DPPH at 517 nm.”

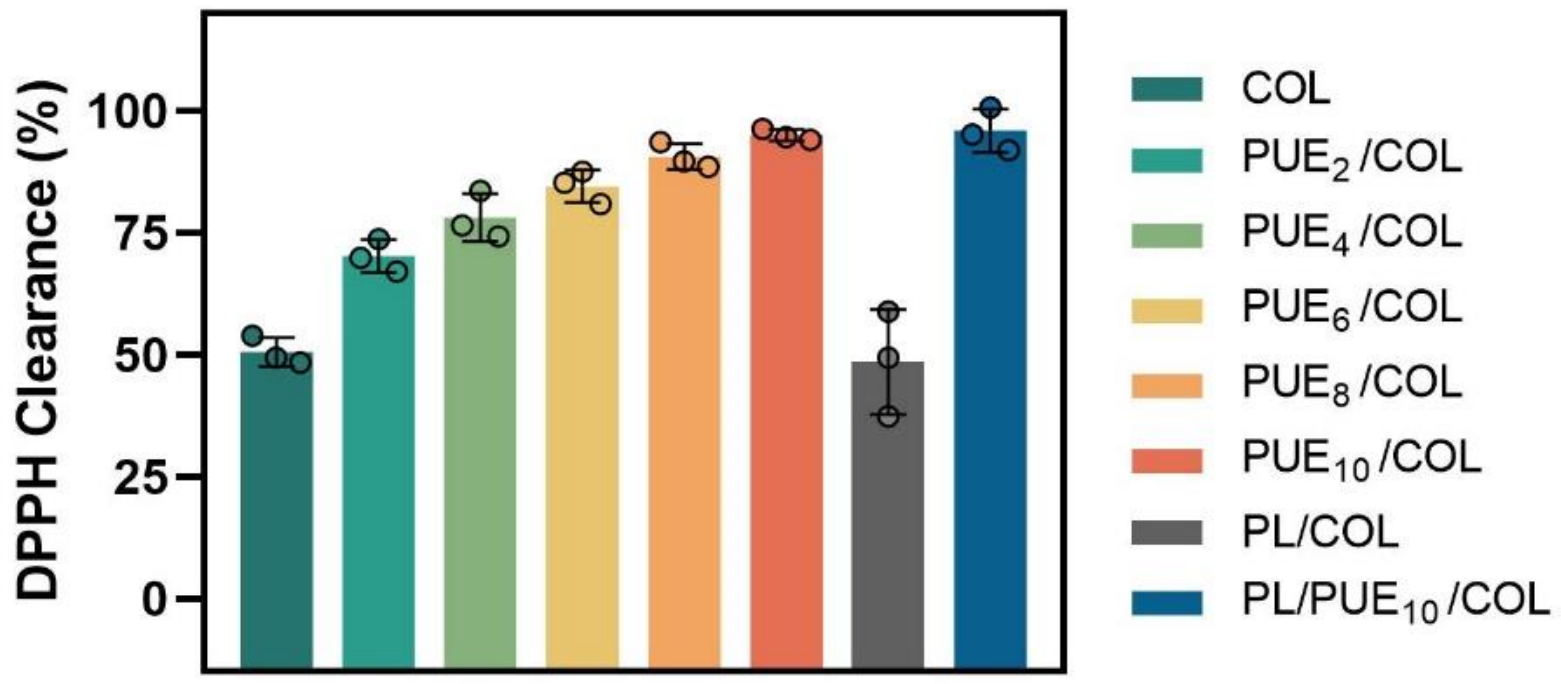

Supplementary Fig. 9. DPPH radical scavenging activity of COL, PUE/COL supramolecular complexes with gradient PUE concentrations (2, 4, 6, 8, 10 mg/mL, denoted as PUE_2/COL to PUE_{10}/COL), PL/COL, and PL/PUE_{10}/COL supramolecular complexes (n=3). All error bars represent mean ± SD. COL recombinant human type I collagen, PL poly-ε-lysine, PUE puerarin, DPPH 2,2-diphenyl-1-picrylhydrazyl. Source data are provided as a Source Data file.

11. Please supplement the Masson staining and immunohistochemistry results of the PL/COL, PUE/COL, and Mino comparison groups.

Response:

Thank you for your suggestions on improving the quality of our manuscript. We agree that including these comprehensive comparison groups is essential for the evaluation of the *in vivo* therapeutic effect.

Following your recommendation, we have supplemented the *in vivo* experiments by adding the PL/COL, PUE/COL, and Minocycline (Mino) groups for both Masson staining and immunohistochemistry analyses (**Supplementary Fig. 22-24**). The updated results further demonstrate the synergistic effect of the PL/PUE/COL supramolecular hydrogel in promoting periodontal collagen deposition and modulating the inflammatory microenvironment, as compared to the single-component controls and the Minocycline treatment.

Furthermore, to ensure the consistency and rigor of the semi-quantitative statistical analysis, all original groups were re-evaluated with the newly added groups to guarantee reliable comparability. All corresponding histological images and semi-quantitative analyses have been

completed and incorporated into the revised manuscript and supplementary information.

Page 30 in the revised manuscript: "To further evaluate the periodontal tissues, Masson staining was performed to assess collagen deposition and organization (Supplementary Fig. 22). The Blank group displayed an intact periodontal tissue structure, with collagen fibers arranged in an orderly and dense manner. In the Periodontitis group, collagen fibers exhibited a disordered and irregular structure, indicative of a disrupted collagenous fiber arrangement due to local inflammation. Similar to the Periodontitis group, PL/COL, PUE/COL, and Mino groups exhibited disorganized collagen deposition, suggesting that single-component hydrogels or conventional antibiotics are insufficient to restore the connective tissue structure in an inflammatory milieu. In contrast, following the treatment with PL/PUE/COL, the periodontal tissues displayed denser and more orderly collagenous fibers.

To evaluate the local inflammatory and regenerative microenvironment, immunohistochemical staining of TNF-α and TGF-β around the periodontal microenvironment was performed. As shown in Supplementary Fig. 23a, compared to the elevated TNF-α expression in the Periodontitis group, all treatments attenuated the inflammatory marker to varying degrees. While PL/COL and minocycline provided partial reductions, both PUE-containing hydrogels (PUE/COL and PL/PUE/COL) more effectively downregulated TNF-α to levels comparable to the Blank group (Supplementary Fig. 23b). We further investigated the local expression levels of TGF-β, a pivotal mediator of tissue repair, to determine the underlying regenerative capacity of the periodontal microenvironment (Supplementary Fig. 24a). Compared to Blank group, TGF-β levels were significantly reduced in the Periodontitis group. While PL/COL, PUE/COL, and minocycline provided only a moderate recovery of TGF-β, the PL/PUE/COL treatment effectively restored its expression, showing no significant difference from the Blank group (Supplementary Fig. 24b). These findings indicate that while PUE attenuates inflammation, the synergistic effect of the supramolecular system is essential to re-establish a microenvironment favorable for tissue regeneration."

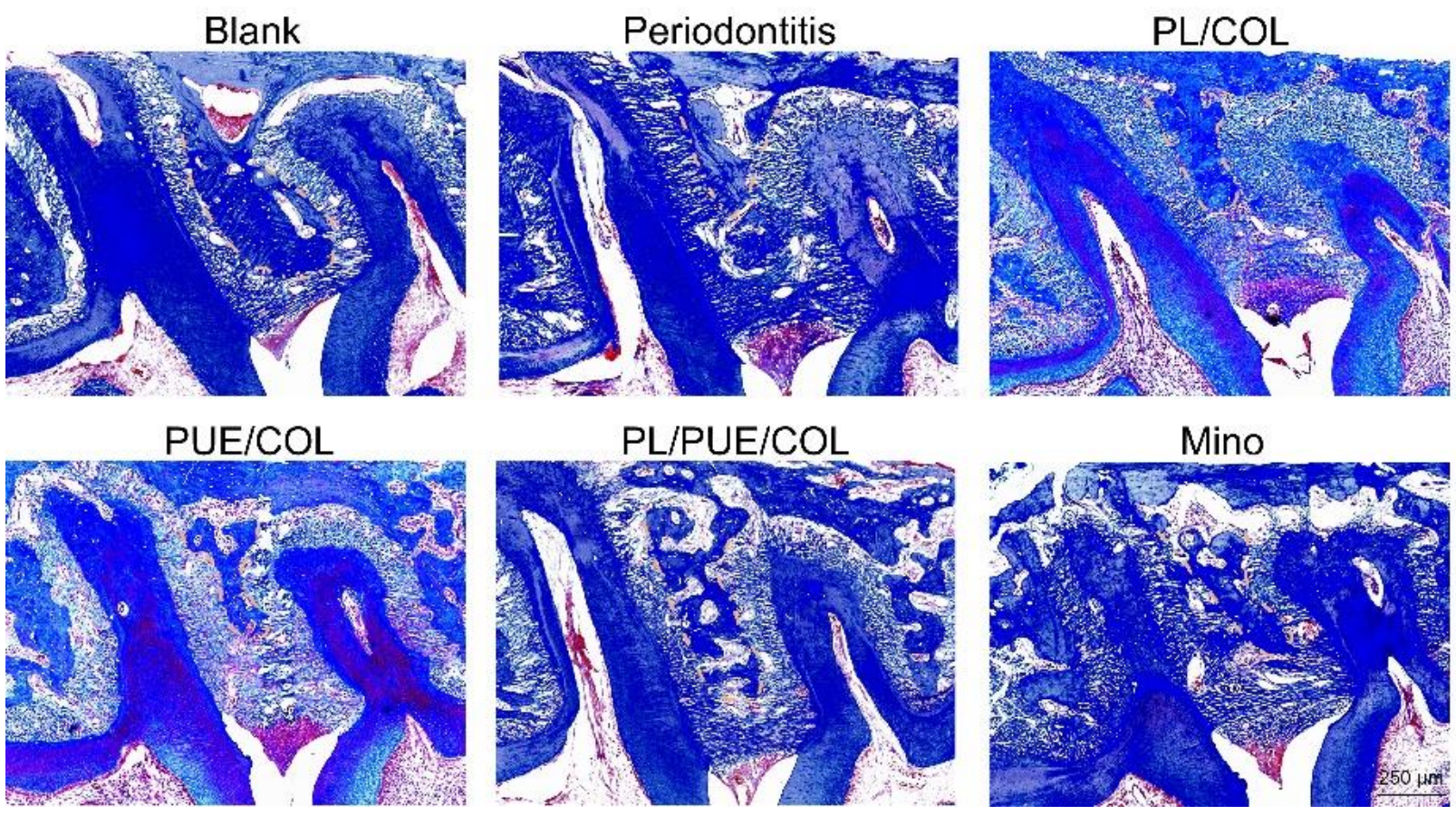

Supplementary Fig. 22. Masson staining of the area between the maxillary first and second molars. Dashed lines indicate the alveolar bone within the lesion area.

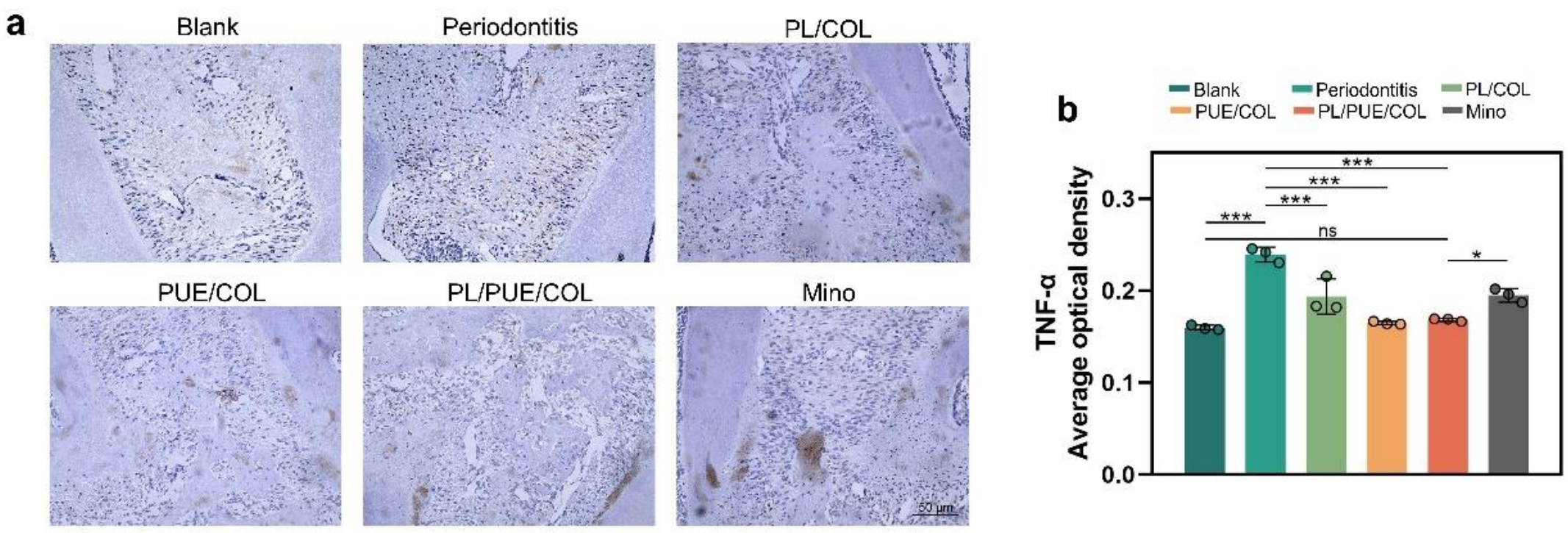

Supplementary Fig. 23. (a) Representative images of immunohistochemical staining for TNF-α in periodontal tissue. (b) Semiquantitative analysis of the average optical density (n=3). All error bars represent mean ± SD. P values were calculated by one-way ANOVA followed by Tukey's post hoc test (*P < 0.05; ***P < 0.001; ns, not significant). TNF-α tumor necrosis factor-α. Source data are provided as a Source Data file.

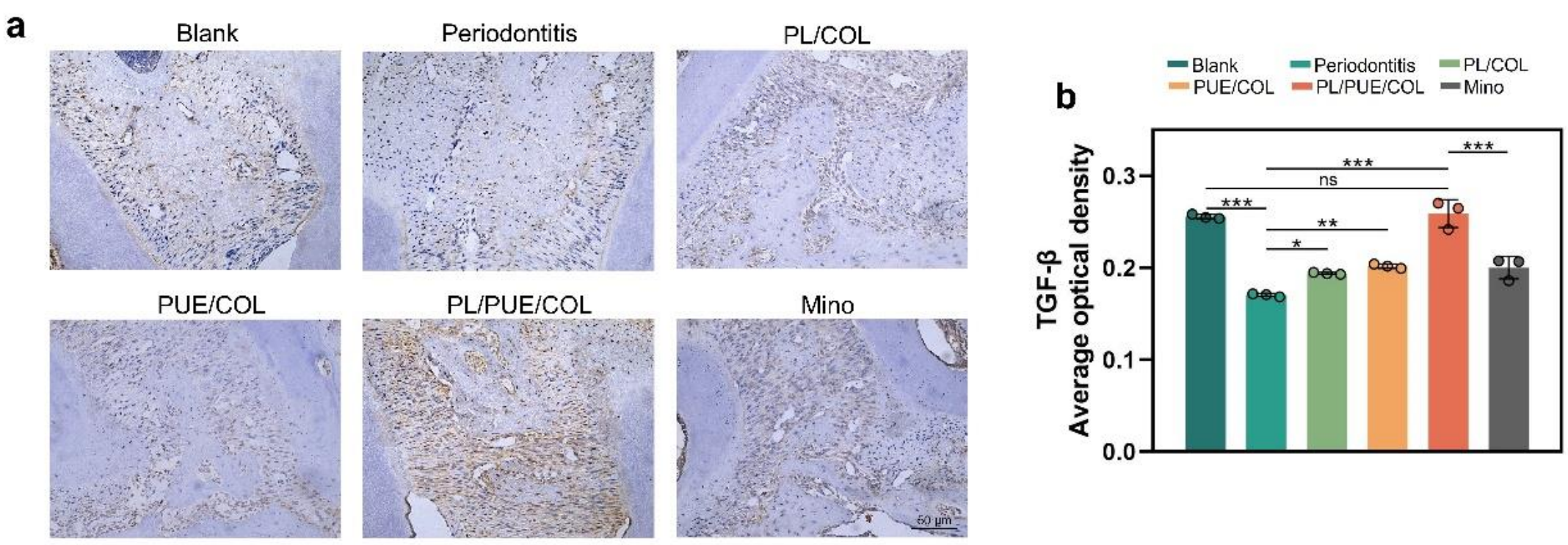

Supplementary Fig. 24. (a) Representative images of immunohistochemical staining for TGF-β in periodontal tissue. (b) Semiquantitative analysis of the average optical density (n=3). All error bars represent mean ± SD. P values were calculated by one-way ANOVA followed by Tukey's post hoc test (*P < 0.05; **P < 0.01; ***P < 0.001; ns, not significant). TGF-β transforming growth factor-β. Source data are provided as a Source Data file.

12. PUE and PL demonstrate a significant synergistic antibacterial effect, which is a very interesting phenomenon. I was wondering, does PL exhibit similar effects with other polyphenolic small molecules? Have you considered conducting related research or planning studies in this area?

Response:

We deeply appreciate the reviewer's insightful question regarding the broader applicability of our supramolecular strategy and the synergistic effects of PL with other small molecules.

Regarding the application to other polyphenols, as many natural polyphenols exhibit poor aqueous solubility which limits their clinical translation, our supramolecular assembly strategy offers a promising platform to overcome this application bottleneck. In the future, we expect to expand this strategy to a broader scope of insoluble polyphenols to systematically validate its universal applicability. Furthermore, investigating whether soluble polyphenols (such as tannic acid) can achieve enhanced tissue penetration ability via similar supramolecular assembling also seems a fascinating and translational direction for our ongoing research.

We will continue to systematically explore these supramolecular systems in our future studies. By deeply investigating their synergistic mechanisms, antibacterial efficacy, and biocompatibility, we aim to establish a theoretical foundation for the efficient screening of supramolecular drug combinations.

13. The data in this paper is relatively comprehensive and the logic is clear, but I would like to know the current limitations of this work. I suggest the author add a discussion on the shortcomings of this study and the issues that need to be addressed in the future.

Response:

We deeply appreciate the reviewer's positive evaluation, as well as the constructive suggestion

to address the limitations of this study.

Following your valuable advice, we have expanded the Discussion section to explicitly address the current limitations. Specifically, while our study demonstrates the efficacy of the supramolecular system, the regulatory pathways governing lysosomal homeostasis, the precise kinetics of intracellular trafficking, and the subcellular interactions require further in-depth exploration.

Furthermore, we plan to integrate advanced nanotechnological strategies to achieve more precise cell- and organelle-specific targeting, which will serve as a crucial step in advancing the supramolecular strategy toward clinical application.

The detailed discussion regarding these limitations and future directions has been incorporated into the revised manuscript as follows:

Page 32 in the revised manuscript: "While the supramolecular co-assembly effectively overcomes PUE's inherent constraints of solubility and permeability, certain limitations remain. The specific regulatory pathways of lysosomal homeostasis need to be further elucidated. The precise intracellular trafficking kinetics and the specific interactions of these complexes at the subcellular structural level also warrant deeper mechanistic investigation. Drawing inspiration from advanced platforms that leverage precise molecular architectures for multi-level biological activities, future studies could integrate our easily fabricated and low-cost supramolecular system with advanced nanotechnology to construct nanomedicines with precisely controlled strategies[73–76]. Building upon this co-assembly mechanism, targeted optimization and functional modification of the constituent components will be essential to achieve cell- and organelle-specific targeting, thereby further advancing the clinical translation of this system."

14. Some latest studies for the therapy of the periodontitis are suggested to be compared with current work in discussion section to highlight the advantages of current strategy such as Bioactive Materials 2025,53:72-83, ACS Nano 2025,19,13341-13355, and Journal of the American Chemical Society 2021,143,15427-15439, but not limited to them.

Response:

We are very grateful to the reviewer for highlighting these excellent studies. These works represent the advanced platforms for periodontitis therapy and have provided inspiration for our

future studies.

We have carefully studied the recommended literature along with other recent related papers. Inspired by these advanced platforms for multi-level biological activities, we expanded our Discussion section. By comparing our current strategy with these nanomedicines, we hope to integrate our easily fabricated and low-cost supramolecular system with advanced nanotechnology to construct nanomedicines with precisely controlled strategies in our future studies. More importantly, it reminds us that to overcome the current limitations, such as the lack of precise intracellular trafficking control, our future studies must integrate with the advanced nanotechnological concepts to achieve cell- and organelle-specific targeting.

The corresponding discussion and the newly added references (Refs. 73-76) have been incorporated into the revised manuscript as follows:

Page 32 in the revised manuscript: "While the supramolecular co-assembly effectively overcomes PUE's inherent constraints of solubility and permeability, certain limitations remain. The specific regulatory pathways of lysosomal homeostasis need to be further elucidated. The precise intracellular trafficking kinetics and the specific interactions of these complexes at the subcellular structural level also warrant deeper mechanistic investigation. Drawing inspiration from advanced platforms that leverage precise molecular architectures for multi-level biological activities, future studies could integrate our easily fabricated and low-cost supramolecular system with advanced nanotechnology to construct nanomedicines with precisely controlled strategies[73–76]. Building upon this co-assembly mechanism, targeted optimization and functional modification of the constituent components will be essential to achieve cell- and organelle-specific targeting, thereby further advancing the clinical translation of this system."

We thank all referees again for their helpful comments and important suggestions, and we hope that the significantly revised manuscript is now acceptable for publication in ***Nature Communications***.

Sincerely,

Zhenming Wang

State Key Laboratory of Oral Diseases & National Center for Stomatology & National Clinical Research Center for Oral Diseases,

West China Hospital of Stomatology,

Sichuan University,

Chengdu, 610041, China.

E-mail: zmwang@scu.edu.cn

Point-by-Point Response to Reviewers

Reviewer #1:

The authors have addressed all my comments, and this manuscript is recommended to be accepted.

Response:

We would like to express our gratitude to you for your time, effort, and highly constructive feedback throughout the review process. Your insightful comments have significantly improved the quality and clarity of our manuscript.

Reviewer #2:

In the revised paper, authors have revised the paper carefully and addressed all the issues raised by reviewers. It can be published as is.

Response:

We sincerely thank you for your thorough evaluation of our manuscript. Your expert suggestions during the review were highly helpful in refining our study. We are deeply grateful for your positive appraisal and your recommendation to publish the manuscript.
